# Association of *leptin receptor* polymorphisms with susceptibility of non‐small cell lung cancer: Evidence from 2249 subjects

**DOI:** 10.1002/cam4.7178

**Published:** 2024-04-25

**Authors:** Weifeng Tang, Jian Wang, Ting Dai, Hao Qiu, Chao Liu, Shuchen Chen, Zhendong Hu

**Affiliations:** ^1^ Departments of Esophageal Surgery and Thoracic Surgery Nanjing Drum Tower Hospital, The Affiliated Hospital of Nanjing University Medical School Nanjing 210008 Jiangsu Province China; ^2^ Department of Cardiothoracic Surgery Affiliated Yixing People's Hospital of Jiangsu University Yixing Jiangsu Province China; ^3^ Department of Pharmacy Affiliated Yixing People's Hospital of Jiangsu University Yixing Jiangsu Province China; ^4^ Department of Laboratory Medicine, School of Medicine Jiangsu University Zhenjiang Jiangsu Province China; ^5^ Department of Cardiothoracic Surgery Affiliated People's Hospital of Jiangsu University Zhenjiang Jiangsu Province China; ^6^ Department of Thoracic Surgery Fujian Medical University Union Hospital Fuzhou Fujian Province China

**Keywords:** LEPR, non‐small cell lung cancer, obesity, overweight, polymorphism

## Abstract

Non‐small cell lung cancer (NSCLC) is increasing dramatically. It is believed that energy metabolism‐related genes could play an important role in etiology of NSCLC. In this study, we sought to assess the correlation between three *LEPR* single nucleotide polymorphisms (rs1137101, rs1137100 and rs6588147) with NSCLS susceptibility. In total, 1193 NSCLC cases and 1056 controls were included. SNPscan™ genotyping method was used to analyze the genotypes of *LEPR* polymorphisms. Compared to rs6588147 GG in *LEPR* gene, this study identified a protective role of *LEPR* rs6588147 GA and GA/AA for the occurrence of NSCLC (GA vs. GG [*p* = 0.021] and GA/AA vs. GG [*p* = 0.030]). As well, we found that a protective role of *LEPR* rs6588147 for the occurrence of non‐SCC subgroup (*p* < 0.05). By logistic regression analysis, we found that the rs6588147 A allele related genotypes might play a protective role for the occurrence of NSCLC in drinking, BMI ≥24 kg/m^2^, smoking and male subgroups. We also found that the rs1137101 A allele related genotypes played a protective role for the occurrence of NSCLC in male, younger participants (under 59 years) and overweight/obesity (BMI ≥24 kg/m^2^) subgroups. We found that *LEPR* A_rs1037100_A_rs1037101_A_rs6588147_ haplotype might play a protective role for the occurrence of NSCLC (*p* = 0.013). In addition, our findings indicated that *LEPR* rs1137100 G>A SNP might increase the risk of lymph node metastases (*p* = 0.038). This study highlights that *LEPR* rs6588147, rs1137101 genotypes and *LEPR* A_rs1037100_A_rs1037101_A_rs6588147_ haplotype are correlated with the occurrence of NSCLC. *LEPR* rs1137100 G>A SNP increases the risk of lymph node metastases.

## 1 INTRODUCTION

With a promoting lung cancer (LC) incidence worldwide,^1^ LC is the leading cause of cancer‐related death. Non‐small cell lung cancer (NSCLC), the most common subtype of lung cancer (LC), is also increasing dramatically in China.^2^ The increasing occurrence of LC might be correlated with some environmental factors (e.g., exposure to tobacco, indoor radon level, air pollution, etc.). Additionally, inactive lifestyle, central obesity and type 2 diabetes mellitus (T2DM) may be implicated in the development of LC.^3^, ^4^, ^5^, ^6^, ^7^, ^8^, ^9^ Over the past half‐century in China, significant lifestyle and daily diet have occurred, leading to a continuous increase in the rates of overweight and obesity.^10^ The role of genes and polymorphisms in the progression and susceptibility of malignancies has been reported in many investigations.^11^, ^12^, ^13^, ^14^ It is believed that metabolism‐related genes play an important role in the etiology of LC.

Leptin (LEP) is a hormone, which is produced by the OB gene. It may be involved in regulating food intake and energy spending. A higher LEP level has been found in overweight and obesity individuals.^15^ LEP receptor (LEPR), a transmembrane cytokine receptor, interacts with LEP and influences the food intake and energy metabolism. Some previous investigations reported that LEPR expression was associated with the proliferation, immune, inflammatory and neoangiogenesis.^16^, ^17^ It was believed that dietary factors were answerable for about a third of overall malignancies.^18^ A number of publications suggested that diet and obesity might contribute to the susceptibility of LC.^19^, ^20^, ^21^, ^22^ Li et al. reported that the LEP and LEPR were more highly expressed in NSCLC cells than in normal lung tissue, which could promote NSCLC by activating the MAPK/ERK and PI3K/AKT pathways.^23^ The level of LEPR could be a useful biomarker for NSCLC prognosis. Xu et al. also suggested that LEPR expression increased in the tissues of NSCLC than in normal lung tissue.^24^ Thus, the LEPR could play a vital role in the etiology and development of NSCLC.

Single nucleotide polymorphism (SNP) is the most common type of variations. Several functional SNPs of *LEPR* have so far been explored for their roles in contributing to the occurrence of cancer. The rs1137100 and rs1137101 SNPs in *LEPR* gene, G → A mutation, were two common missense SNPs. It is well known that missense SNP may lead to an amino acid change in LEPR. Li et al. first studied the association of rs1137100 and rs1137101 SNPs in *LEPR* with NSCLC susceptibility. Compared with AA, they found that *LEPR* rs1137101 GG, increased 3.12‐fold risk of NSCLC in Chinese populations.^25^ And they also observed that *LEPR* rs1137101 GG carriers of NSCLC patients might have a poor survival than rs1137101 AG and AA carriers.^25^ Subsequently, in Caucasians, a case–control study explored the relationship of rs1137101 with LC risk and reported null association.^26^ Rs6588147 G>A in *LEPR*, an intron variant, was found to be correlated with a susceptibility of colon carcinoma in Caucasians.^27^ Recently, in Asian populations, the relationship of rs6588147 in *LEPR* with cancer risk was reported with inconsistent findings. Qiu et al. found that *LEPR* rs6588147 was associated with the increased risk of esophageal squamous cell carcinoma.^28^ However, Lin et al. and Zhang et al. reported that *LEPR* rs6588147 decreased the risk of colorectal cancer and hepatocellular carcinoma, respectively.^29^, ^30^ Thus, the correlation of *LEPR* rs6588147 with the risk of cancer was conflicting. Here, the rs1137101, rs1137100 and rs6588147 loci in *LEPR* gene were selected based on the previous studies mentioned above. We performed an investigation to assess the correlation of *LEPR* SNPs with NSCLC susceptibility.

## 2 MATERIALS AND METHODS

### 2.1 Statement of ethics

Ethics Committee of Fujian Medical University Union Hospital approved this investigation. Each participation was informed the aim of this study. The research has been carried out in accordance with the World Medical Association Declaration of Helsinki, and that all subjects provided informed consent.

### 2.2 Study populations

The NSCLC patients were recruited between January 2014 and June 2018 at Fujian Medical University Union Hospital (Fujian Province, China) and Nanjing Medical University Zhenjiang Medical College (Jiangsu Province, China). All NSCLC cases were residents of Fujian, Jiangsu Provinces and the surrounding areas. When enrollment was carried out, the following criteria were used: (1) without history of other malignancy, (2) before any treatment for NSCLC, and (3) Chinese Han ethnicity. The following criteria were used for exclusion: (1) with a second primary malignancy, (2) after radiotherapy and chemotherapy treatment, and (3) diagnosed as small cell lung cancer cases. NSCLC stage was classified according to AJCC 8.0 version (2017) of LC. Of the 1193 NSCLC patients, 182 (15.26%) were squamous cell carcinoma (SCC), and 1011 (84.74%) were non‐SCC. In addition, at the same time, 1056 healthy controls were included from the institutions mentioned above. Controls were full‐matched with NSCLC patients on age and sex (*p* = 0.330 and 0.425, respectively). Healthy control subjects were Chinese Han populations who were unrelated to NSCLC patients. In this study, body mass index (BMI) was assessed by height and weight, which were collected by two authors. BMI ≥24 kg/m^2^ was considered as the criteria of overweight and obesity.^31^, ^32^ Subjects who consumed one cigarette per day for more than 1 year were considered as “smokers”.^33^ Individuals who drank more than three times a week for longer than 6 months were considered to be “alcohol users”.^33^ Each participant completed a questionnaire and donated 2 mL blood sample. The information including smoking, drinking, age, sex, ethnicity, height, weight and results of pathology was collected.

### 2.3 Genotyping

The blood sample was stored with EDTA anticoagulant. A “Salting‐out” method was used to extract DNA by using the DNA Purification Kit (Promega, Madison, USA). A 260/280 ratio checking was used to confirm the purity of each DNA sample. The rs1137101, rs1137100 and rs6588147 genotypes were determined by SNPscan™ method.^34^, ^35^ In ABI 2720 thermal cycler, the ligation reaction was conducted. Then a 48‐plex fluorescence PCR was carried out. The PCR products were analyzed by capillary electrophoresis in an ABI 3730XL sequencer. The information of *LEPR* genotypes was read by GeneMapper 4.1 software (Applied Biosystems, Foster City, CA, USA). For each of *LEPR* SNPs, 112 DNA samples were chosen randomly and assayed the *LEPR* genotypes to confirm the veracity of our results. And consistent *LEPR* genotypes were found.

### 2.4 Statistical analysis

Student's *t*‐test was harnessed to treat continuous variable comparison. Intergroup difference of *LEPR* genotypes, BMI, alcohol consumption, sex, smoking and age was determined by chi‐square test. By logistic regression analysis, crude and adjusted odds ratios (ORs) and 95% confidence intervals (CIs) were calculated to assess the relationships in homozygous, dominant, heterozygous and recessive models in overall or subgroup analysis. All statistical analyses were performed by using SAS 9.4 software (SAS Institute, Inc.). The value of Hardy–Weinberg equilibrium (HWE) was obtained by online goodness‐of‐fit chi‐square test.^36^, ^37^, ^38^, ^39^, ^40^, ^41^ An internet haplotype construction method, SHESIS software (http://analysis.bio‐x.cn/myAnalysis.php), was harnessed to get the haplotypes of LEPR gene.^38^, ^42^, ^43^, ^44^, ^45^ A *p* < 0.05 was used as the criterion of the significance level. Additionally, Bonferroni correction was applied to adjust for multiple testing.^46^

## 3 RESULTS

### 3.1 Characteristics

Table 1 shows the information and baseline characteristic of 1193 NSCLC cases and 1056 controls. Age and sex were well matched (*p* = 0.330 and *p* = 0.425). Of the NSCLC patients, 182 were squamous cell carcinoma (SCC), and 1011 were non‐SCC. The stage of NSCLC patients was classified according to AJCC 8.0 version (2017). Among them, 703 were stage I, 87 were stage II, 222 were stage III and 181 were stage IV. We summarized the chromosomal location, region, and minor allele frequency (MAF) information in Table 2. For *LEPR* SNPs, success rate of genotyping was more than 99%. The value of HWE was also calculated and shown in Table 2 (all *p* > 0.05). MAFs of *LEPR* rs1137101, rs1137100 and rs6588147 (0.124, 0.159 and 0.151) could be obtained in Table 2, which were analogous to the data of the Chinese populations. Raw data of genotypes and characteristics were summarized in Table S1

**TABLE 1:** Distribution of selected demographic variables and risk factors in NSCLC cases and controls.

Variable	NSCLC Cases (*n* = 1193)		Controls (*n* = 1056)		*p* ^a^
	*n*	%	*n*	%	
Age (years)					
<59	535	44.84	452	42.80	0.330
≥59	658	55.16	604	57.20	
Sex					
Male	642	53.81	586	55.65	0.425
Female	551	46.19	470	44.35	
Smoking status					
Never	757	63.45	857	81.16	**<0.001**
Ever	436	36.55	199	18.84	
Alcohol use					
Never	946	79.30	967	91.83	**<0.001**
Ever	247	20.70	89	8.17	
BMI (kg/m^2^)					
<24	801	67.14	571	54.07	**<0.001**
≥24	392	32.86	485	45.93	
Type of NSCLC					
SCC	182	15.26			
Non‐SCC	1011	84.74			
Stage					
I	703	58.93			
II	87	7.29			
III	222	18.61			
IV	181	15.17			

*Note*: Bold values are statistically significant (*p* < 0.05).

Abbreviations: BMI, body mass index; NSCLC, non‐small‐cell lung cancer; SCC, squamous cell carcinoma.

^a^ Two‐sided *χ* ^2^ test and student's *t*‐test.

**TABLE 2:** Primary information for *LEPR* rs1137100, rs1137101 and rs6588147 polymorphisms.

Genotyped polymorphisms	*LEPR* rs1137100 G>A	*LEPR* rs1137101 G>A	*LEPR* rs6588147 G>A
Chromosome	1	1	1
Position_37	66,036,441	66,058,513	65,935,494
Region	nonsynon_exon4	nonsynon_exon6	Intron 2
Regulome DB Score^a^	5	5	4
Function	p.Lys109Arg	p.Gln223Arg	/
MAF for Chinese in database	0.169	0.111	0.150
MAF in our controls (*n* = 1056)	0.159	0.124	0.151
*P* value for HWE test in our controls	0.205	0.736	0.149
Genotyping method	SNPscan	SNPscan	SNPscan
Genotyping value (%)	99.33	99.42	99.33

^a^ http://www.regulomedb.org/.

Abbreviations: HWE, Hardy–Weinberg equilibrium; MAF, minor allele frequency.

### 3.2 Association of LEPR polymorphisms with NSCLC

The *LEPR* rs1137101, rs1137100 and rs6588147 genotypes in overall NSCLC cases and different pathological subtype are shown in Table 3. The *LEPR* genotypes in control group are also summarized in Table 3.

**TABLE 3:** The frequencies of *LEPR* rs1137100, rs1137101 and rs6588147 polymorphisms in NSCLC patients and controls.

Genotype	Overall NSCLC cases (*n* = 1193)		SCC cases (*n* = 182)		Non‐SCC cases (*n* = 1011)		Controls (*n* = 1056)	
	*n*	%	*n*	%	*n*	%	*n*	%
*LEPR* rs1137100 G>A								
GG	847	71.72	133	73.89	714	71.33	740	70.28
GA	311	26.33	45	25.00	266	26.57	292	27.73
AA	23	1.95	2	1.11	21	2.10	21	1.99
A allele	357	15.11	49	13.61	308	15.38	334	15.86
*LEPR* rs1137101 G>A								
233	940	79.39	147	81.22	793	79.06	806	76.62
233	233	19.68	33	18.23	200	19.94	231	21.96
AA	11	0.93	1	0.55	10	1.00	15	1.43
A allele	255	10.77	35	9.67	220	10.97	261	12.40
*LEPR* rs6588147 G>A								
GG	892	75.53	142	78.89	750	74.93	753	71.51
GA	265	22.44	36	20.00	229	22.88	282	26.78
AA	24	2.03	2	1.11	22	2.20	18	1.71
A allele	313	13.25	40	11.11	273	13.64	318	15.10

Abbreviations: NSCLC, non‐small‐cell lung cancer; SCC, squamous cell carcinoma.

Compared to rs6588147 GG in *LEPR* gene, we identified a protective role of *LEPR* rs6588147 GA and GA/AA for the occurrence of NSCLC in two genetic models (GA vs. GG: OR = 0.79, 95% CI = 0.65–0.96, *p* = 0.019 and AA/GA vs. GG: OR = 0.81, 95% CI = 0.61–0.98, *p* = 0.032, Table 4). By logistic regression analysis, the protective role of rs6588147 GA and GA/AA genotypes was also confirmed (GA vs. GG: adjusted OR = 0.79, 95% CI = 0.64–0.97, *p* = 0.021 and AA/GA vs. GG: adjusted OR = 0.80, 95% CI = 0.66–0.98, *p* = 0.030, Table 4).

**TABLE 4:** Overall and stratified analyses of *LEPR* rs1137100, rs1137101 and rs6588147 polymorphisms with NSCLC.

Genotype	Overall NSCLC cases (*n* = 1193) vs. Controls (1056)				Non‐SCC cases (*n* = 1011) vs. Controls (1056)				SCC cases (*n* = 182) vs. Controls (1056)			
	Crude OR (95% CI)	*p*	Adjusted OR^a^ (95% CI)	*p*	Crude OR (95% CI)	*p*	Adjusted OR^a^ (95% CI)	*p*	Crude OR (95% CI)	*p*	Adjusted OR^a^ (95% CI)	*p*
*LEPR* rs1137100 G>A												
GA vs. GG	0.93 (0.77–1.12)	0.452	0.96 (0.79–1.17)	0.685	0.94 (0.78–1.15)	0.564	0.95 (0.77–1.16)	0.593	0.86 (0.60–1.23)	0.408	0.99 (0.65–1.51)	0.962
AA vs. GG	0.96 (0.53–1.74)	0.886	0.99 (0.53–1.86)	0.980	1.04 (0.56–1.91)	0.909	1.03 (0.55–1.95)	0.919	0.53 (0.12–2.29)	0.395	0.80 (0.15–4.22)	0.791
AA/GA vs. GG	0.93 (0.78–1.12)	0.453	0.96 (0.80–1.17)	0.694	0.95 (0.79–1.15)	0.600	0.95 (0.78–1.16)	0.626	0.84 (0.58–1.20)	0.325	0.98 (0.65–1.48)	0.922
AA vs. GG/GA	0.98 (0.54–1.77)	0.937	1.00 (0.54–1.88)	0.991	1.05 (0.57–1.94)	0.868	1.05 (0.56–1.98)	0.880	0.55 (0.13–2.38)	0.425	0.80 (0.15–4.21)	0.793
*LEPR* rs1137101 G>A												
GA vs. GG	0.87 (0.71–1.06)	0.165	0.87 (0.70–1.07)	0.186	0.88 (0.71–1.09)	0.240	0.86 (0.69–1.08)	0.195	0.78 (0.52–1.17)	0.237	0.88 (0.55–1.40)	0.578
AA vs. GG	0.63 (0.29–1.38)	0.246	0.65 (0.29–1.46)	0.296	0.69 (0.30–1.52)	0.344	0.67 (0.29–1.54)	0.347	0.37 (0.05–2.79)	0.332	0.82 (0.09–7.32)	0.857
AA/GA vs. GG	0.85 (0.70–1.04)	0.113	0.85 (0.69–1.05)	0.135	0.87 (0.70–1.07)	0.183	0.85 (0.69–1.06)	0.147	0.76 (0.51–1.13)	0.174	0.87 (0.55–1.38)	0.565
AA vs. GG/GA	0.65 (0.30–1.42)	0.279	0.67 (0.30–1.50)	0.330	0.70 (0.31–1.56)	0.378	0.69 (0.30–1.58)	0.385	0.38 (0.05–2.93)	0.356	0.84 (0.09–7.50)	0.875
*LEPR* rs6588147 G>A												
GA vs. GG	**0.79 (0.65–0.96)**	**0.019**	**0.79 (0.64–0.97)**	**0.021**	**0.82 (0.67–1.00)**	**0.047**	**0.79 (0.64–0.97)**	**0.025**	0.68 (0.46–1.00)	0.050	0.73 (0.47–1.14)	0.166
AA vs. GG	1.13 (0.61–2.09)	0.708	1.04 (0.55–1.99)	0.898	1.23 (0.65–2.31)	0.525	1.15 (0.60–2.20)	0.679	0.59 (0.14–2.57)	0.481	0.77 (0.14–4.36)	0.772
AA/GA vs. GG	**0.81 (0.67–0.98)**	**0.032**	**0.80 (0.66–0.98)**	**0.030**	0.84 (0.69–1.02)	0.081	**0.81 (0.66–0.99)**	**0.042**	**0.67 (0.46–0.99)**	**0.041**	0.73 (0.47–1.13)	0.162
AA vs. GG/GA	1.19 (0.64–2.21)	0.576	1.11 (0.58–2.11)	0.757	1.29 (0.69–2.42)	0.426	1.22 (0.64–2.33)	0.551	0.65 (0.15–2.81)	0.561	0.83 (0.15–4.68)	0.835

*Note*: Bold values are statistically significant (*p* < 0.05).

Abbreviations: NSCLC, non‐small‐cell lung cancer; SCC, squamous cell carcinoma.

^a^ Adjusted for age, sex, smoking, drinking and body mass index.

In overall comparison, we did not find any relationship of *LEPR* rs1137101 and rs1137100 loci with the risk of NSCLC.

### 3.3 Association of LEPR polymorphisms with NSCLC in subgroup analysis

By logistic regression analysis, compared to rs6588147 GG, a protective role of *LEPR* rs6588147 GA and GA/AA for the occurrence of non‐SCC subgroup was found (GA vs. GG: adjusted OR = 0.79, 95% CI = 0.64–0.97, *p* = 0.025 and AA/GA vs. GG: adjusted OR = 0.81, 95% CI = 0.66–0.99, *p* = 0.042, Table 4).

In subgroup analysis, the genotypes of rs1137101 in *LEPR* gene are summarized in Table 5. We found that the rs1137101 A allele played a protective role for the occurrence of NSCLC in <59 years (GA vs. GG: adjusted OR = 0.71, 95% CI = 0.51–0.98, *p* = 0.040 and AA/GA vs. GG: adjusted OR = 0.72, 95% CI = 0.52–0.99, *p* = 0.044), BMI ≥24 kg/m^2^ (GA vs. GG: adjusted OR = 0.69, 95% CI = 0.49–0.97, *p* = 0.035 and AA/GA vs. GG: adjusted OR = 0.67, 95% CI = 0.47–0.94, *p* = 0.019) and male subgroups (GA vs. GG: adjusted OR = 0.68, 95% CI = 0.50–0.92, *p* = 0.014 and AA/GA vs. GG: adjusted OR = 0.70, 95% CI = 0.57–0.95, *p* = 0.020).

**TABLE 5:** Stratified analyses between *LEPR* rs1137101 G>A polymorphism and NSCLC risk by sex, age, BMI, smoking status and alcohol consumption.

Variable	*LEPR* rs1137101 G>A (case/control)^a^			Adjusted OR^b^ (95% CI); *p*				
	GG	GA	AA	GG	GA	AA	GA/AA	AA vs. (GA/GG)
Sex								
Male	521/444	111/134	6/5	1.00	**0.68 (0.50–0.92); *p*: 0.014**	1.30 (0.36–4.66); *p*: 0.688	**0.70 (0.57–0.95); *p*: 0.020**	1.40 (0.39–5.02); *p*: 0.604
Female	419/362	122/97	5/10	1.00	1.11 (0.82–1.51); *p*: 0.506	0.43 (0.14–1.29); *p*: 0.132	1.05 (0.78–1.41); *p*: 0.770	0.42 (0.14–1.26); *p*: 0.121
Age								
<59	429/338	95/105	6/6	1.00	**0.71 (0.51–0.98); *p*: 0.040**	0.92 (0.27–3.06); *p*: 0.887	**0.72 (0.52–0.99); *p*: 0.044**	0.98 (0.29–3.27); *p*: 0.975
≥59	511/468	138/126	5/9	1.00	1.01 (0.76–1.34); *p*: 0.937	0.53 (0.17–1.65); *p*: 0.275	0.98 (0.74–1.29); *p*: 0.885	0.53 (0.17–1.64); *p*: 0.272
Smoking status								
Never	589/652	155/187	7/14	1.00	0.91 (0.71–1.16); *p*: 0.435	0.51 (0.20–1.30); *p*: 0.159	0.88 (0.69–1.12); *p*: 0.290	0.52 (0.21–1.33); *p*: 0.172
Ever	351/154	78/44	4/1	1.00	0.76 (0.50–1.16); *p*: 0.203	1.99 (0.22–18.23); *p*: 0.545	0.79 (0.52–1.19); *p*: 0.258	2.10 (0.23–19.20); *p*: 0.513
Alcohol consumption								
Never	744/743	187/206	9/15	1.00	0.91 (0.72–1.14); *p*: 0.403	0.56 (0.24–1.33); *p*: 0.190	0.88 (0.70–1.11); *p*: 0.276	0.58 (0.25–1.36); *p*: 0.206
Ever	196/63	46/25	2/0	1.00	0.61 (0.35–1.08); *p*: 0.092	–	0.63 (0.36–1.12); *p*: 0.116	–
BMI (kg/m^2^)								
<24	625/446	161/116	8/7	1.00	1.00 (0.76–1.32); *p*: 0.986	0.87 (0.31–2.47); *p*: 0.792	0.99 (0.76–1.30); *p*: 0.943	0.87 (0.31–2.46); *p*: 0.792
≥24	315/360	72/115	3/8	1.00	**0.69 (0.49–0.97); *p*: 0.035**	0.36 (0.09–1.48); *p*: 0.155	**0.67 (0.47–0.94); *p*: 0.019**	0.39 (0.09–1.60); *p*: 0.189

^a^ For *LEPR* rs1137101 G>A, the genotyping was successful in 1184 (99.25%) NSCLC cases, and 1052 (99.62%) controls.

^b^ Adjusted for multiple comparisons [age, sex, smoking status, BMI and alcohol consumption (besides stratified factors accordingly)] in a logistic regression model.

*LEPR* rs6588147 genotypes in different subgroup are shown in Table 6. Among drinking, BMI ≥24 kg/m^2^, smoking and male subgroups, we found that the rs6588147 A allele might play a protective role for the occurrence of NSCLC in GA vs. GG and GA/AA vs. GG genetic models (GA vs. GG: adjusted *p* = 0.003 and AA/GA vs. GG: adjusted *p* = 0.004 for male subgroup, GA vs. GG: adjusted *p* = 0.007 and AA/GA vs. GG: adjusted *p* = 0.019 for smoking subgroup, GA vs. GG: adjusted *p* = 0.006 and AA/GA vs. GG: adjusted *p* = 0.016 for drinking subgroup and GA vs. GG: adjusted *p* = 0.036 and AA/GA vs. GG: adjusted *p* = 0.039 for BMI ≥24 kg/m^2^ subgroup). When Bonferroni correction was applied to adjust for multiple testing, we found that the rs6588147 A allele might play a protective role for the occurrence of NSCLC among drinking, smoking and male subgroups.

**TABLE 6:** Stratified analyses between *LEPR* rs6588147 G>A polymorphism and NSCLC risk by sex, age, BMI, smoking status and alcohol consumption.

Variable	*LEPR* rs6588147 G>A (case/control)^a^			Adjusted OR^b^ (95% CI); *p*				
	GG	GA	AA	GG	GA	AA	GA/AA	AA vs. (GA/GG)
Sex								
Male	493/410	131/165	12/9	1.00	**0.65 (0.49–0.86); *p*: 0.003**	0.92 (0.35–2.40); *p*: 0.865	**0.66 (0.50–0.88); *p*: 0.004**	1.02 (0.39–2.65); *p*: 0.965
Female	399/343	134/117	12/9	1.00	0.94 (0.70–1.27); *p*: 0.698	1.09 (0.45–2.68); *p*: 0.844	0.95 (0.72–1.27); *p*: 0.748	1.11 (0.46–2.71); *p*: 0.818
Age								
<59	398/319	122/123	7/8	1.00	0.80 (0.59–1.09); *p*: 0.149	0.73 (0.25–2.16); *p*: 0.568	0.79 (0.59–1.07); *p*: 0.132	0.77 (0.26–2.28); *p*: 0.641
≥59	494/434	143/159	17/10	1.00	0.78 (0.59–1.02); *p*: 0.072	1.25 (0.55–2.85); *p*: 0.601	0.81 (0.62–1.05); *p*: 0.114	1.33 (0.58–3.02); *p*: 0.503
Smoking status								
Never	554/613	182/225	13/16	1.00	0.87 (0.69–1.10); *p*: 0.246	0.83 (0.39–1.78); *p*: 0.635	0.87 (0.69–1.09); *p*: 0.223	0.86 (0.40–1.84); *p*: 0.700
Ever	338/140	83/57	11/2	1.00	**0.58 (0.39–0.86); *p*: 0.007**	2.08 (0.45–9.67); *p*: 0.349	**0.63 (0.43–0.93); *p*: 0.019**	2.38 (0.52–11.01); *p*: 0.267
Alcohol consumption								
Never	706/698	213/249	18/18	1.00	0.84 (0.68–1.05); *p*: 0.129	0.90 (0.45–1.79); *p*: 0.765	0.85 (0.69–1.05); *p*: 0.129	0.94 (0.48–1.86); *p*: 0.858
Ever	186/55	52/33	6/0	1.00	**0.47 (0.28–0.81); *p*: 0.006**	–	**0.52 (0.31–0.89); *p*: 0.016**	–
BMI (kg/m^2^)								
<24	589/405	184/154	18/10	1.00	0.83 (0.64–1.08); *p*: 0.164	1.14 (0.51–2.56); *p*: 0.744	0.85 (0.66–1.10); *p*: 0.231	1.20 (0.54–2.67); *p*: 0.659
≥24	303/348	81/128	6/8	1.00	**0.70 (0.50–0.98); *p*: 0.036**	0.85 (0.28–2.62); *p*: 0.778	**0.71 (0.51–0.98); *p*: 0.039**	0.93 (0.30–2.84); *p*: 0.893

^a^ For *LEPR* rs6588147 G>A, the genotyping was successful in 1181 (98.99%) NSCLC cases, and 1053 (99.72%) controls.

^b^ Adjusted for multiple comparisons (age, sex, smoking status, BMI and alcohol consumption [besides stratified factors accordingly]) in a logistic regression model.

As summarized in Table 7, we found that the rs1137100 A allele did not influence the occurrence of NSCLC in different subgroup.

**TABLE 7:** Stratified analyses between *LEPR* rs1137100 G>A polymorphism and NSCLC risk by sex, age, BMI, smoking status and alcohol consumption.

Variable	*LEPR* rs1137100 G>A (case/control)^a^			Adjusted OR^b^ (95% CI); *p*				
	GG	GA	AA	GG	GA	AA	GA/AA	AA vs. (GA/GG)
Sex								
Male	480/409	149/166	7/9	1.00	0.77 (0.58–1.01); *p*: 0.063	0.65 (0.21–2.01); *p*: 0.458	0.76 (0.58–1.00); *p*: 0.052	0.70 (0.23–2.16); *p*: 0.537
Female	367/331	162/126	16/12	1.00	1.20 (0.90–1.59); *p*: 0.207	1.28 (0.58–2.81); *p*: 0.536	1.21 (0.92–1.59); *p*: 0.179	1.22 (0.56–2.65); *p*: 0.625
Age								
<59	379/320	142/124	6/6	1.00	0.93 (0.69–1.26); *p*: 0.650	1.13 (0.33–3.90); *p*: 0.843	0.94 (0.70–1.26); *p*: 0.684	1.15 (0.34–3.96); *p*: 0.820
≥59	468/420	169/168	17/15	1.00	0.98 (0.75–1.27); *p*: 0.847	1.01 (0.48–2.11); *p*: 0.980	0.98 (0.76–1.26); *p*: 0.860	1.02 (0.49–2.12); *p*: 0.965
Smoking status								
Never	519/600	212/235	18/19	1.00	1.03 (0.82–1.30); *p*: 0.772	1.05 (0.53–2.06); *p*: 0.892	1.04 (0.83–1.29); *p*: 0.759	1.04 (0.53–2.03); *p*: 0.914
Ever	328/140	99/57	5/2	1.00	0.76 (0.52–1.13); *p*: 0.174	0.84 (0.15–4.54); *p*: 0.837	0.77 (0.52–1.12); *p*: 0.173	0.89 (0.17–4.82); *p*: 0.894
Alcohol consumption								
Never	665/678	253/269	19/18	1.00	0.96 (0.78–1.18); *p*: 0.670	1.12 (0.57–2.22); *p*: 0.738	0.97 (0.79–1.19); *p*: 0.737	1.14 (0.58–2.24); *p*: 0.709
Ever	182/62	58/23	4/3	1.00	0.90 (0.50–1.60); *p*: 0.715	0.39 (0.08–1.88); *p*: 0.241	0.84 (0.48–1.46); *p*: 0.527	0.40 (0.09–1.91); *p*: 0.253
BMI (kg/m^2^)								
<24	572/411	202/151	17/7	1.00	1.00 (0.77–1.28); *p*: 0.973	1.76 (0.71–4.39); *p*: 0.223	1.03 (0.80–1.32); *p*: 0.817	1.77 (0.71–4.38); *p*: 0.220
≥24	275/329	109/141	6/14	1.00	0.89 (0.65–1.22); *p*: 0.467	0.44 (0.16–1.26); *p*: 0.125	0.85 (0.63–1.15); *p*: 0.298	0.46 (0.16–1.29); *p*: 0.140

^a^ For *LEPR* rs1137100 G>A, the genotyping was successful in 1181 (98.99%) NSCLC cases, and 1053 (99.72%) controls.

^b^ Adjusted for multiple comparisons (age, sex, smoking status, BMI and alcohol consumption [besides stratified factors accordingly]) in a logistic regression model.

### 3.4 SNP haplotypes

After using the SHESIS software, we obtained eight *LEPR* haplotypes (Table 8). We found that *LEPR* A_rs1037100_A_rs1037101_A_rs6588147_ haplotype might play a protective role for the occurrence of NSCLC (OR = 0.73, 95% CI = 0.57–0.94, *p* = 0.013).

**TABLE 8:** *LEPR* haplotype frequencies (%) in cases and controls and risk of NSCLC.

Haplotypes	case (*n* = 2386)		control (*n* = 2112)		Crude OR (95% CI)	*p*
	*n*	%	*n*	%		
G_rs1037100_G_rs1037101_G_rs6588147_	1828	77.39	1604	76.24	Reference	
G_rs1037100_G_rs1037101_A_rs6588147_	131	5.55	124	5.89	0.93 (0.72–1.20)	0.559
A_rs1037100_A_rs1037101_A_rs6588147_	127	5.38	152	7.22	**0.73 (0.57–0.94)**	**0.013**
A_rs1037100_ **G**_rs1037101_G_rs6588147_	94	3.98	80	3.80	1.03 (0.76–1.40)	0.845
A_rs1037100_A_rs1037101_G_rs6588147_	82	3.47	67	3.18	1.07 (0.77–1.49)	0.672
A_rs1037100_G_rs1037101_A_rs6588147_	54	2.29	35	1.66	1.35 (0.88–2.08)	0.166
G_rs1037100_A_rs1037101_G_rs6588147_	45	1.91	35	1.66	1.13 (0.72–1.76)	0.597
G_rs1037100_A_rs1037101_A_rs6588147_	1	0.04	7	0.33	0.13 (0.02–1.02)	0.051

*Note*: With the order of *LEPR* rs1137100 G>A, rs1137101 G>A and rs6588147 G>A in gene position. Compared with LEPR G_rs1037100_G_rs1037101_G_rs6588147_ haplotype, LEPR A_rs1037100_A_rs1037101_A_rs6588147_ haplotype might decreased the risk of NSCLC.

### 3.5 Association of LEPR polymorphisms with lymph node metastases and stage

We analyzed the association of *LEPR* polymorphisms with lymph node metastases and stage. We found that *LEPR* rs1137100 G>A SNP might increase the risk of lymph node metastases of NSCLC (OR = 1.35, 95% CI = 1.02–1.79, *p* = 0.038, Table 9).

**TABLE 9:** Analyses of *LEPR* rs1137100, rs1137101 and rs6588147 polymorphisms with lymph node metastases and stage.

Genotype	Lymph node metastases (*n* = 381) vs. non‐metastases (*n* = 812)				Staging III/IV (*n* = 403) vs. Staging I/II (*n* = 790)			
	Crude OR (95% CI)	*p*	Adjusted OR^a^ (95% CI)	*p*	Crude OR (95% CI)	*p*	Adjusted OR^a^ (95% CI)	*p*
*LEPR* rs1137100 G>A								
GA vs. GG	1.16 (0.88–1.53)	0.294	1.30 (0.98–1.74)	0.072	1.05 (0.80–1.37)	0.751	1.16 (0.87–1.54)	0.318
AA vs. GG	1.72 (0.75–3.98)	0.202	2.16 (0.91–5.13)	0.080	1.28 (0.55–3.00)	0.567	1.55 (0.65–3.72)	0.323
AA/GA vs. GG	1.19 (0.91–1.55)	0.208	**1.35 (1.02–1.79)**	**0.038**	1.06 (0.81–1.38)	0.694	1.17 (0.89–1.55)	0.258
AA vs. GG/GA	1.65 (0.72–3.80)	0.238	1.99 (0.84–4.69)	0.118	1.26 (0.54–2.94)	0.591	1.48 (0.62–3.52)	0.381
*LEPR* rs1137101 G>A								
GA vs. GG	1.00 (0.74–1.36)	0.990	1.04 (0.76–1.44)	0.795	0.88 (0.65–1.20)	0.416	0.91 (0.66–1.24)	0.538
AA vs. GG	2.58 (0.78–8.53)	0.120	2.81 (0.82–9.61)	0.099	2.31 (0.70–7.64)	0.169	2.45 (0.72–8.32)	0.151
AA/GA vs. GG	1.04 (0.77–1.41)	0.794	1.09 (0.80–1.49)	0.598	0.92 (0.68–1.24)	0.560	0.94 (0.69–1.29)	0.708
AA vs. GG/GA	2.56 (0.78–8.45)	0.122	2.78 (0.81–9.48)	0.103	2.35 (0.71–7.76)	0.160	2.49 (0.73–8.44)	0.144
*LEPR* rs6588147 G>A								
GA vs. GG	1.00 (0.74–1.34)	0.988	1.09 (0.80–1.48)	0.591	0.88 (0.66–1.18)	0.386	0.94 (0.70–1.27)	0.692
AA vs. GG	1.54 (0.67–3.50)	0.307	1.43 (0.60–3.38)	0.420	1.63 (0.72–3.68)	0.240	1.54 (0.66–3.60)	0.317
AA/GA vs. GG	1.03 (0.78–1.37)	0.827	1.11 (0.83–1.49)	0.486	0.92 (0.70–1.22)	0.579	0.98 (0.73–1.31)	0.883
AA vs. GG/GA	1.53 (0.67–3.48)	0.309	1.39 (0.59–3.28)	0.456	1.67 (0.74–3.76)	0.216	1.55 (0.66–3.61)	0.311

*Note*: Bold values are statistically significant (*p* < 0.05).

^a^ Adjusted for age, sex, smoking, drinking and body mass index.

## 4 DISCUSSION

*LEPR* is a transmembrane cytokine receptor, which combines to LEP and regulates the food intake and energy metabolism. It was believed that the expression of *LEPR* was associated with the proliferation, immune, inflammatory and neoangiogenesis.^16^, ^17^, ^47^, ^48^, ^49^ In this study, we conducted a case–control study and explored the role of *LEPR* SNPs in the development of NSCLC.

Rs6588147, an intron SNP, located on Chromosome 1: 65469811 (GRCh38).^27^ The function of *LEPR* rs6588147 was unknown. Our investigation showed that the A allele of rs6588147 could decrease the susceptibility of NSCLC in overall comparison (GA vs. GG: adjusted *p* = 0.021 and AA/GA vs. GG: adjusted *p* = 0.030). Additionally, the role of decreasing the susceptibility of NSCLC was also confirmed among drinking, smoking and male subgroups, even a Bonferroni correction applied to adjust for multiple testing. Lin et al. found that this SNP decreased colorectal cancer risk in the male subgroup,^29^ which was similar to our findings. Slettery and colleagues first explored the relationship of *LEPR* rs6588147 with the occurrence of colon cancer and indicated that A allele of rs6588147 had a tendency to reduce the occurrence of colon cancer in Caucasians.^27^ Additionally, in Chinese populations, previous studies reported that rs6588147 locus in *LEPR* gene was associate with a lower susceptibility of colorectal cancer and hepatocellular carcinoma.^29^, ^30^ Our observations were similar to them. However, several investigations suggested that A allele of rs6588147 had a higher susceptibility of esophageal squamous cell carcinoma and breast cancer.^28^, ^50^ Due to the limited sample sizes, the role of rs6588147 on cancer susceptibility may be controversial. In the future, more investigations focusing on environmental factors are needed.

*LEPR* Arg223Gln (rs1137101), a G → A variation on exon 6, is a missense SNP.^51^ The role of *LEPR* rs1137101 in the development of cancer has been studied.^52^, ^53^ In the extracellular region, this missense polymorphism results in an Arg→Gln substitution.^51^ Boothby‐Shoemaker et al. reported that the lower FGFR1 and LEPR expression could delay cancer progression via JAK2 signaling.^54^ Stefan et al. suggested that A allele of *LEPR* rs1137101 might reduce the binding of LEP with LEPR.^55^ A recent investigation also identified that the AA genotype of *LEPR* rs1137101 with a lower binding ability to its ligand could decrease the activity of Ras/ERK1/2 and JAK2/STAT3 the signaling pathways and played a protective role in carcinogenesis.^56^ Two case–control studies had been performed to investigate the relationship of *LEPR* Arg223Gln with the susceptibility of NSCLC in different ethnicities. Li et al. reported that A allele (223Gln) of *LEPR* rs1137101 reduced the susceptibility of NSCLC in Chinese population.^25^ In addition, in Caucasians, Unsal et al. found that combined genotypes of *LEP* 2548G>A and *LEPR* rs1137101 SNPs might have a protective effect against the occurrence of LC.^26^ In the present investigation, the relationship between rs1137101 and a decreased susceptibility of NSCLC was found in male, younger (<59 years) and overweight/obesity (BMI ≥24 kg/m^2^) subgroups, which was similar to previous studies.

The association of *LEPR* rs1137100 SNP with risk of cancer were inconsistent. In this study, we found that there was null association between *LEPR* rs1137100 SNP with risk of NSCLC. However, we found that *LEPR* rs1137100 G>A SNP might increase the risk of lymph node metastases (*p* = 0.038). To the best of our knowledge, this study was the first investigation in which the relationship of *LEPR* rs1137100 G>A SNP with lymph node metastases was found. In the future, more studies should be conducted to explore the potential association of *LEPR* rs1137100 SNP with lymph node metastases.

In the future, more studies should be conducted to explore the potential association between *LEPR* rs1137100 and the risk of NSCLC.

As listed in Table 7, compared to *LEPR* G_rs1037100_G_rs1037101_G_rs6588147_ haplotype, we found that the frequency of *LEPR* A_rs1037100_A_rs1037101_A_rs6588147_ haplotype was significantly low in NSCLC group (*p* = 0.013), which is similar to the previous findings.^57^ To the best of knowledge, we first focused on the correlation of *LEPR* haplotypes with NSCLC susceptibility. These findings suggested that *LEPR* A_rs1037100_A_rs1037101_A_rs6588147_ haplotype could be used in screening high‐risk populations for NSCLC.

There were some limitations in the present study. Firstly, for lack of data on lifestyle and dietary factors (low intake of fruit and veggies, poor diet, physical inactivity, high outdoor ambient air pollution, and cooking oil fume, etc.), we could not explore an interaction of *LEPR* rs1137101, rs1137100 and rs6588147 polymorphisms with these conditions. Secondly, although the sample sizes were relatively large, the number of the participants in some subgroups was limited. Thirdly, given the hospital‐based design, the selection bias might have happened. Fourthly, only three functional SNPs of *LEPR* gene were studied in this investigation. Other vital *LEPR* SNPs should not been ignored. Fifthly, diabetes mellitus and family history of participants were not collected. In future studies, these vital risk factors should not be overlooked. Sixthly, when designed this case–control study, we did not consider the association between *LEPR* SNPs and EGFR status of NSCLC. Finally, this investigation was carried out in China. In the future, more well‐designed studies should be performed to explore the association of *LEPR* SNPs with the risk of NSCLC in other ethnicities.

In conclusion, the present study highlights that LEPR rs6588147 and rs1137101 genotypes and *LEPR* Ars1037100Ars1037101Ars6588147 haplotype are correlated with the occurrence of NSCLC. *LEPR* rs1137100 G>A SNP increases the risk of lymph node metastases. Additionally, rs6588147 A allele could decrease the susceptibility of NSCLC was also confirmed among drinking, smoking and male subgroups, even a Bonferroni correction applied to adjust for multiple testing. The current investigation attempted to point out the potential contribution of *LEPR* SNPs to NSCLC etiology and also for screening high susceptibility individuals for prevention and early detection of NSCLC. To further explore *LEPR* SNPs and NSCLC risk, more case–control studies in diverse ethnicities along with functional and expressional characterizations of the *LEPR* SNPs are needed.

## AUTHOR CONTRIBUTIONS

**Weifeng Tang:** Data curation (equal); formal analysis (lead); funding acquisition (equal); writing – original draft (lead). **Jian Wang:** Formal analysis (equal); funding acquisition (equal); writing – original draft (equal). **Ting Dai:** Writing – original draft (equal). **Hao Qiu:** Data curation (equal). **Chao Liu:** Formal analysis (equal). **Shuchen Chen:** Conceptualization (equal); software (equal). **Zhendong Hu:** Conceptualization (equal); software (equal).

## FUNDING INFORMATION

This study was supported in part by Scientific Research Project of Wuxi Health Commission (Grant number: M202153) and Initial Research Project for Talents of Nanjing Drum Tower Hospital, The Affiliated Hospital of Nanjing University Medical School (Grant number: RC2021‐009).

## CONFLICT OF INTEREST STATEMENT

The authors have no potential conflicts of interest.

## ETHICS STATEMENT

The experimental protocol was established, according to the ethical guidelines of the Helsinki Declaration and approved by Ethics Committee of Fujian Medical University Union Hospital.

## CONSENT TO PARTICIPATE

Written informed consent was obtained from participants. This information could be obtained from “Statement of Ethics” section.

## CONSENT FOR PUBLICATION

All authors declare that they consent for publication.

## Supporting information


Table S1.

## Data Availability

Full data are available via an online supplementary material. Raw data of genotypes and characteristics were summarized in Table S1.
