# Supplementary material for: Association of leptin receptor polymorphisms with susceptibility of non‐small cell lung cancer: Evidence from 2249 subjects
Source: Cancer Med. 2024 Apr 25;13(8):e7178. doi: 10.1002/cam4.7178 (PMC11043686; doi:10.1002/cam4.7178)
Supplement: Supplementary file 1 — Table S1. [file CAM4-13-e7178-s001.docx]

**Table S1.**Raw data of *LEPR* genotypes and characteristics

| Participants | Sex (male: 1; female: 2) | Age (year) | Smoking (Yes: 1, No: 0) | Drinking (Yes: 1, No: 0) | BMI (≥24: 1, <24: 0) | Pathology | rs1137100 | rs1137101 | rs6588147 |
| --- | --- | --- | --- | --- | --- | --- | --- | --- | --- |
| NSCLC-0001 | 1 | 67 | 1 | 0 | 0 | SCC | G/G | G/G | G/G |
| NSCLC-0002 | 2 | 82 | 0 | 0 | 0 | SCC | G/G | G/G | G/G |
| NSCLC-0003 | 1 | 53 | 0 | 0 | 0 | SCC | G/G | G/G | G/G |
| NSCLC-0004 | 1 | 65 | 1 | 0 | 0 | SCC | G/G | G/G | G/G |
| NSCLC-0005 | 1 | 77 | 0 | 0 | 0 | SCC | G/A | G/A | G/G |
| NSCLC-0006 | 2 | 43 | 0 | 0 | 0 | SCC | G/G | G/G | G/G |
| NSCLC-0007 | 1 | 71 | 1 | 1 | 0 | SCC | A/A | G/A | A/A |
| NSCLC-0008 | 1 | 59 | 1 | 1 | 0 | SCC | G/G | G/G | G/G |
| NSCLC-0009 | 2 | 64 | 1 | 1 | 0 | SCC | G/G | G/G | G/G |
| NSCLC-0010 | 1 | 71 | 1 | 0 | 0 | SCC | G/A | G/G | G/G |
| NSCLC-0011 | 1 | 53 | 1 | 1 | 0 | SCC | G/A | G/A | G/G |
| NSCLC-0012 | 1 | 50 | 1 | 1 | 0 | SCC | G/G | G/G | G/G |
| NSCLC-0013 | 1 | 58 | 1 | 0 | 1 | SCC | G/G | G/G | G/G |
| NSCLC-0014 | 1 | 56 | 1 | 1 | 1 | SCC | G/G | G/G | G/G |
| NSCLC-0015 | 1 | 75 | 1 | 0 | 1 | SCC | G/G | G/G | G/G |
| NSCLC-0016 | 1 | 65 | 1 | 0 | 0 | SCC | G/G | G/G | G/G |
| NSCLC-0017 | 1 | 61 | 1 | 0 | 0 | SCC | G/G | G/G | G/G |
| NSCLC-0018 | 1 | 67 | 1 | 0 | 0 | SCC | G/G | G/G | G/G |
| NSCLC-0019 | 1 | 65 | 1 | 0 | 0 | SCC | G/G | G/G | G/G |
| NSCLC-0020 | 1 | 68 | 1 | 0 | 0 | SCC | G/A | G/A | G/A |
| NSCLC-0021 | 1 | 56 | 1 | 0 | 0 | SCC | G/G | G/G | G/G |
| NSCLC-0022 | 1 | 57 | 1 | 0 | 1 | SCC | G/A | G/A | G/G |
| NSCLC-0023 | 1 | 64 | 1 | 0 | 1 | SCC | G/A | G/A | G/A |
| NSCLC-0024 | 1 | 71 | 1 | 0 | 0 | SCC | G/G | G/G | G/G |
| NSCLC-0025 | 1 | 78 | 1 | 0 | 0 | SCC | G/G | G/G | G/G |
| NSCLC-0026 | 1 | 63 | 1 | 0 | 0 | SCC | G/G | G/G | G/G |
| NSCLC-0027 | 1 | 57 | 1 | 0 | 0 | SCC | G/A | G/G | G/G |
| NSCLC-0028 | 1 | 46 | 1 | 0 | 0 | SCC | G/G | G/G | G/G |
| NSCLC-0029 | 1 | 67 | 1 | 0 | 0 | SCC | G/A | G/A | G/G |
| NSCLC-0030 | 1 | 52 | 1 | 0 | 1 | SCC | G/G | G/G | G/G |
| NSCLC-0031 | 1 | 63 | 1 | 0 | 0 | SCC | G/G | G/G | G/G |
| NSCLC-0032 | 1 | 54 | 1 | 0 | 0 | SCC | G/G | G/G | G/G |
| NSCLC-0033 | 1 | 51 | 1 | 0 | 0 | SCC | G/G | G/G | G/G |
| NSCLC-0034 | 1 | 63 | 1 | 0 | 0 | SCC | G/G | G/G | G/G |
| NSCLC-0035 | 1 | 58 | 1 | 0 | 1 | SCC | G/G | G/G | G/G |
| NSCLC-0036 | 1 | 67 | 1 | 1 | 0 | SCC | G/G | G/G | G/A |
| NSCLC-0037 | 1 | 62 | 1 | 0 | 0 | SCC | G/A | G/G | G/G |
| NSCLC-0038 | 1 | 66 | 1 | 0 | 0 | SCC | G/G | G/G | G/G |
| NSCLC-0039 | 1 | 53 | 1 | 0 | 1 | SCC | G/G | G/A | G/G |
| NSCLC-0040 | 1 | 53 | 1 | 0 | 1 | SCC | G/G | G/G | G/G |
| NSCLC-0041 | 2 | 72 | 0 | 0 | 0 | SCC | G/A | G/A | A/A |
| NSCLC-0042 | 1 | 53 | 1 | 0 | 0 | SCC | G/G | G/G | G/G |
| NSCLC-0043 | 1 | 64 | 1 | 0 | 0 | SCC | G/A | G/A | G/A |
| NSCLC-0044 | 1 | 76 | 0 | 0 | 0 | SCC | G/G | G/G | G/G |
| NSCLC-0045 | 1 | 46 | 1 | 0 | 0 | SCC | G/G | G/G | G/G |
| NSCLC-0046 | 1 | 64 | 0 | 1 | 0 | SCC | G/A | G/G | G/A |
| NSCLC-0047 | 1 | 77 | 1 | 0 | 0 | SCC | G/G | G/G | G/G |
| NSCLC-0048 | 1 | 62 | 1 | 1 | 1 | SCC | G/A | G/A | G/G |
| NSCLC-0049 | 1 | 87 | 1 | 1 | 1 | SCC | G/G | G/G | G/G |
| NSCLC-0050 | 1 | 65 | 1 | 0 | 0 | SCC | G/G | G/G | G/G |
| NSCLC-0051 | 1 | 54 | 1 | 0 | 1 | SCC | G/A | G/G | G/A |
| NSCLC-0052 | 1 | 67 | 1 | 1 | 0 | SCC | G/G | G/G | G/G |
| NSCLC-0053 | 2 | 69 | 0 | 0 | 1 | SCC | G/A | G/A | G/A |
| NSCLC-0054 | 1 | 55 | 1 | 1 | 0 | SCC | G/G | G/G | G/G |
| NSCLC-0055 | 1 | 68 | 1 | 1 | 1 | SCC | G/A | G/G | G/G |
| NSCLC-0056 | 1 | 55 | 0 | 0 | 0 | SCC | G/G | G/G | G/G |
| NSCLC-0057 | 1 | 62 | 1 | 0 | 0 | SCC | G/G | G/G | G/G |
| NSCLC-0058 | 2 | 67 | 0 | 0 | 1 | SCC | G/G | G/G | G/G |
| NSCLC-0059 | 1 | 69 | 1 | 1 | 0 | SCC | G/G | G/G | G/G |
| NSCLC-0060 | 1 | 51 | 1 | 0 | 0 | SCC | G/G | G/G | G/G |
| NSCLC-0061 | 1 | 62 | 1 | 0 | 1 | SCC | G/A | G/G | G/G |
| NSCLC-0062 | 1 | 55 | 1 | 1 | 1 | SCC | G/G | G/G | G/A |
| NSCLC-0063 | 1 | 43 | 1 | 1 | 0 | SCC | G/A | G/G | G/A |
| NSCLC-0064 | 1 | 62 | 1 | 0 | 0 | SCC | G/A | G/G | G/G |
| NSCLC-0065 | 1 | 61 | 1 | 0 | 0 | SCC | G/A | G/A | G/A |
| NSCLC-0066 | 1 | 61 | 1 | 1 | 0 | SCC | G/G | G/G | G/G |
| NSCLC-0067 | 2 | 64 | 0 | 0 | 1 | SCC | G/A | G/A | G/A |
| NSCLC-0068 | 1 | 76 | 0 | 0 | 0 | SCC | G/G | G/G | G/G |
| NSCLC-0069 | 1 | 71 | 1 | 1 | 0 | SCC | G/G | G/G | G/G |
| NSCLC-0070 | 1 | 80 | 1 | 0 | 0 | SCC | G/G | G/G | G/G |
| NSCLC-0071 | 1 | 64 | 1 | 1 | 1 | SCC | G/G | G/G | G/G |
| NSCLC-0072 | 1 | 65 | 1 | 1 | 0 | SCC | G/G | G/G | G/G |
| NSCLC-0073 | 1 | 64 | 1 | 0 | 0 | SCC | G/A | G/G | G/A |
| NSCLC-0074 | 1 | 77 | 1 | 0 | 0 | SCC | G/G | G/G | G/G |
| NSCLC-0075 | 1 | 60 | 1 | 0 | 0 | SCC | G/G | G/G | G/G |
| NSCLC-0076 | 2 | 55 | 0 | 0 | 0 | SCC | G/G | G/G | G/G |
| NSCLC-0077 | 1 | 77 | 0 | 0 | 0 | SCC | G/A | G/A | G/A |
| NSCLC-0078 | 1 | 78 | 1 | 0 | 0 | SCC | G/G | G/G | G/G |
| NSCLC-0079 | 1 | 70 | 1 | 0 | 0 | SCC | G/G | G/G | G/G |
| NSCLC-0080 | 1 | 81 | 0 | 0 | 0 | SCC | G/A | G/G | G/G |
| NSCLC-0081 | 1 | 58 | 1 | 0 | 0 | SCC | G/A | G/G | G/A |
| NSCLC-0082 | 1 | 68 | 0 | 0 | 0 | SCC | G/G | G/G | G/G |
| NSCLC-0083 | 1 | 70 | 1 | 1 | 0 | SCC | G/A | G/A | G/G |
| NSCLC-0084 | 2 | 56 | 0 | 0 | 0 | SCC | G/G | G/G | G/G |
| NSCLC-0085 | 1 | 51 | 1 | 0 | 1 | SCC | G/G | G/G | G/G |
| NSCLC-0086 | 1 | 48 | 1 | 0 | 0 | SCC | G/G | G/A | G/G |
| NSCLC-0087 | 1 | 57 | 1 | 0 | 0 | SCC | G/G | G/G | G/G |
| NSCLC-0088 | 1 | 66 | 0 | 0 | 0 | SCC | G/G | G/G | G/G |
| NSCLC-0089 | 1 | 62 | 0 | 0 | 0 | SCC | G/G | G/G | G/A |
| NSCLC-0090 | 1 | 44 | 1 | 1 | 0 | SCC | G/A | G/G | G/A |
| NSCLC-0091 | 1 | 55 | 1 | 0 | 0 | SCC | G/G | G/G | G/G |
| NSCLC-0092 | 1 | 64 | 1 | 1 | 0 | SCC | G/G | G/G | G/G |
| NSCLC-0093 | 2 | 65 | 0 | 0 | 0 | SCC | G/A | G/A | G/A |
| NSCLC-0094 | 1 | 62 | 0 | 0 | 0 | SCC | G/G | G/G | G/G |
| NSCLC-0095 | 1 | 70 | 1 | 1 | 0 | SCC | G/G | G/G | G/G |
| NSCLC-0096 | 1 | 62 | 1 | 0 | 0 | SCC | G/G | G/G | G/G |
| NSCLC-0097 | 1 | 53 | 1 | 0 | 0 | SCC | G/G | G/G | G/G |
| NSCLC-0098 | 1 | 75 | 1 | 1 | 1 | SCC | G/A | G/A | G/G |
| NSCLC-0099 | 1 | 59 | 1 | 1 | 1 | SCC | G/A | G/A | G/A |
| NSCLC-0100 | 1 | 80 | 1 | 0 | 0 | SCC | G/G | G/G | G/G |
| NSCLC-0101 | 1 | 50 | 1 | 1 | 1 | SCC | G/G | G/G | G/G |
| NSCLC-0102 | 1 | 71 | 1 | 1 | 0 | SCC | G/G | G/G | G/G |
| NSCLC-0103 | 1 | 64 | 1 | 1 | 1 | SCC | G/G | G/G | G/G |
| NSCLC-0104 | 1 | 48 | 1 | 1 | 0 | SCC | G/G | G/G | G/G |
| NSCLC-0105 | 2 | 46 | 0 | 0 | 1 | SCC | G/G | G/G | G/G |
| NSCLC-0106 | 1 | 59 | 1 | 1 | 1 | SCC | G/G | G/G | G/G |
| NSCLC-0107 | 1 | 65 | 1 | 1 | 0 | SCC | G/G | G/G | G/G |
| NSCLC-0108 | 1 | 55 | 1 | 0 | 0 | SCC | G/G | G/G | G/A |
| NSCLC-0109 | 1 | 60 | 1 | 0 | 0 | SCC | G/G | G/G | G/G |
| NSCLC-0110 | 2 | 53 | 0 | 0 | 0 | SCC | G/G | G/G | G/A |
| NSCLC-0111 | 1 | 72 | 1 | 1 | 0 | SCC | G/G | G/G | G/G |
| NSCLC-0112 | 1 | 59 | 1 | 1 | 0 | SCC | G/G | G/G | G/G |
| NSCLC-0113 | 1 | 62 | 1 | 1 | 0 | SCC | G/A | G/G | G/A |
| NSCLC-0114 | 1 | 57 | 1 | 1 | 0 | SCC | G/G | G/G | G/G |
| NSCLC-0115 | 1 | 51 | 1 | 0 | 0 | SCC | G/A | G/G | G/G |
| NSCLC-0116 | 2 | 61 | 0 | 0 | 0 | SCC | G/G | G/G | G/A |
| NSCLC-0117 | 1 | 74 | 1 | 0 | 1 | SCC | G/A | G/G | G/G |
| NSCLC-0118 | 1 | 52 | 1 | 1 | 1 | SCC | G/G | G/G | G/G |
| NSCLC-0119 | 1 | 53 | 1 | 1 | 0 | SCC | G/G | G/G | G/G |
| NSCLC-0120 | 1 | 76 | 1 | 0 | 0 | SCC | G/A | G/A | G/A |
| NSCLC-0121 | 1 | 73 | 1 | 0 | 0 | SCC | G/G | G/G | G/G |
| NSCLC-0122 | 1 | 59 | 1 | 0 | 0 | SCC | G/G | G/A | G/G |
| NSCLC-0123 | 1 | 62 | 1 | 0 | 1 | SCC | G/G | G/G | G/G |
| NSCLC-0124 | 1 | 49 | 1 | 1 | 0 | SCC | G/G | G/G | G/G |
| NSCLC-0125 | 1 | 52 | 1 | 0 | 0 | SCC | G/A | G/A | G/A |
| NSCLC-0126 | 1 | 57 | 1 | 0 | 0 | SCC | G/G | G/A | G/G |
| NSCLC-0127 | 1 | 69 | 0 | 0 | 0 | SCC | G/G | G/G | G/G |
| NSCLC-0128 | 1 | 57 | 1 | 1 | 0 | SCC | G/G | G/G | G/G |
| NSCLC-0129 | 1 | 56 | 0 | 0 | 0 | SCC | G/A | G/A | G/A |
| NSCLC-0130 | 1 | 55 | 1 | 1 | 0 | SCC | G/G | G/A | G/G |
| NSCLC-0131 | 1 | 62 | 1 | 1 | 0 | SCC | G/G | G/G | G/G |
| NSCLC-0132 | 1 | 63 | 0 | 0 | 1 | SCC | G/G | G/G | G/G |
| NSCLC-0133 | 1 | 67 | 1 | 1 | 0 | SCC | G/G | G/G | G/G |
| NSCLC-0134 | 1 | 66 | 1 | 0 | 0 | SCC | G/G | G/G | G/G |
| NSCLC-0135 | 1 | 72 | 1 | 1 | 0 | SCC | G/G | G/G | G/G |
| NSCLC-0136 | 1 | 66 | 1 | 1 | 0 | SCC | G/G | G/G | G/G |
| NSCLC-0137 | 1 | 57 | 1 | 1 | 0 | SCC | G/G | G/G | G/G |
| NSCLC-0138 | 1 | 65 | 1 | 1 | 0 | SCC | G/G | G/G | G/G |
| NSCLC-0139 | 1 | 73 | 1 | 0 | 0 | SCC | G/G | G/G | G/A |
| NSCLC-0140 | 1 | 77 | 1 | 0 | 0 | SCC | G/G | G/G | G/G |
| NSCLC-0141 | 2 | 52 | 0 | 0 | 0 | SCC | G/G | G/G | G/G |
| NSCLC-0142 | 1 | 59 | 1 | 1 | 0 | SCC | G/G | G/G | G/G |
| NSCLC-0143 | 1 | 64 | 0 | 0 | 0 | SCC | G/A | G/A | G/A |
| NSCLC-0144 | 1 | 74 | 1 | 1 | 1 | SCC | G/G | G/G | G/G |
| NSCLC-0145 | 1 | 58 | 1 | 1 | 0 | SCC | G/G | G/G | G/G |
| NSCLC-0146 | 1 | 65 | 0 | 1 | 0 | SCC | G/A | G/A | G/A |
| NSCLC-0147 | 1 | 66 | 1 | 1 | 0 | SCC | G/G | G/G | G/G |
| NSCLC-0148 | 1 | 60 | 1 | 0 | 1 | SCC | G/G | G/G | G/G |
| NSCLC-0149 | 1 | 56 | 1 | 0 | 0 | SCC | G/A | A/A | G/A |
| NSCLC-0150 | 1 | 66 | 1 | 1 | 0 | SCC | ? | ? | ? |
| NSCLC-0151 | 1 | 59 | 1 | 1 | 0 | SCC | G/G | G/G | G/G |
| NSCLC-0152 | 1 | 57 | 1 | 0 | 1 | SCC | G/A | G/G | G/A |
| NSCLC-0153 | 1 | 68 | 0 | 0 | 0 | SCC | G/G | G/G | G/G |
| NSCLC-0154 | 1 | 64 | 1 | 1 | 0 | SCC | G/G | G/G | G/G |
| NSCLC-0155 | 1 | 48 | 0 | 0 | 0 | SCC | G/G | G/G | G/G |
| NSCLC-0156 | 1 | 64 | 0 | 0 | 0 | SCC | G/G | G/G | G/G |
| NSCLC-0157 | 1 | 56 | 1 | 0 | 0 | SCC | G/A | G/G | G/G |
| NSCLC-0158 | 1 | 62 | 1 | 1 | 0 | SCC | G/G | G/G | G/A |
| NSCLC-0159 | 1 | 60 | 1 | 0 | 0 | SCC | G/G | G/G | G/G |
| NSCLC-0160 | 1 | 57 | 1 | 1 | 0 | SCC | G/G | G/A | G/G |
| NSCLC-0161 | 1 | 61 | 0 | 0 | 1 | SCC | G/G | G/G | G/A |
| NSCLC-0162 | 1 | 74 | 0 | 0 | 0 | SCC | G/G | G/G | G/G |
| NSCLC-0163 | 1 | 60 | 1 | 0 | 1 | SCC | G/G | G/G | G/A |
| NSCLC-0164 | 1 | 52 | 1 | 0 | 0 | SCC | G/G | G/G | G/G |
| NSCLC-0165 | 1 | 71 | 1 | 1 | 1 | SCC | G/G | G/G | G/G |
| NSCLC-0166 | 1 | 55 | 0 | 0 | 1 | SCC | G/A | G/A | G/A |
| NSCLC-0167 | 1 | 68 | 1 | 1 | 0 | SCC | G/A | G/A | G/G |
| NSCLC-0168 | 1 | 49 | 0 | 0 | 0 | SCC | G/G | G/G | G/G |
| NSCLC-0169 | 2 | 73 | 0 | 0 | 0 | SCC | G/A | G/G | G/G |
| NSCLC-0170 | 1 | 70 | 1 | 0 | 0 | SCC | G/G | G/G | G/G |
| NSCLC-0171 | 1 | 62 | 1 | 0 | 1 | SCC | G/G | G/G | G/G |
| NSCLC-0172 | 1 | 51 | 1 | 1 | 0 | SCC | G/G | G/G | G/G |
| NSCLC-0173 | 1 | 55 | 1 | 1 | 0 | SCC | G/G | G/G | G/G |
| NSCLC-0174 | 1 | 55 | 1 | 1 | 0 | SCC | G/G | G/G | G/G |
| NSCLC-0175 | 2 | 53 | 0 | 0 | 0 | SCC | G/G | G/G | G/A |
| NSCLC-0176 | 1 | 70 | 1 | 1 | 0 | SCC | G/G | G/A | G/G |
| NSCLC-0177 | 1 | 57 | 1 | 0 | 0 | SCC | ? | G/G | ? |
| NSCLC-0178 | 1 | 54 | 1 | 0 | 0 | SCC | G/G | G/G | G/G |
| NSCLC-0179 | 1 | 72 | 1 | 1 | 0 | SCC | G/G | G/G | G/G |
| NSCLC-0180 | 1 | 71 | 1 | 1 | 0 | SCC | G/G | G/G | G/G |
| NSCLC-0181 | 1 | 72 | 1 | 0 | 0 | SCC | G/A | G/G | G/G |
| NSCLC-0182 | 2 | 60 | 1 | 0 | 0 | SCC | A/A | G/A | G/A |
| NSCLC-0183 | 2 | 47 | 0 | 0 | 0 | Non-SCC | G/G | G/A | G/G |
| NSCLC-0184 | 1 | 60 | 0 | 0 | 0 | Non-SCC | G/G | G/G | G/G |
| NSCLC-0185 | 1 | 61 | 0 | 0 | 0 | Non-SCC | G/G | G/G | G/G |
| NSCLC-0186 | 2 | 63 | 0 | 0 | 0 | Non-SCC | G/G | G/A | G/G |
| NSCLC-0187 | 2 | 52 | 0 | 0 | 0 | Non-SCC | G/A | G/G | G/G |
| NSCLC-0188 | 2 | 57 | 0 | 0 | 1 | Non-SCC | G/G | G/G | G/G |
| NSCLC-0189 | 1 | 71 | 1 | 0 | 0 | Non-SCC | G/G | G/G | G/G |
| NSCLC-0190 | 2 | 45 | 0 | 0 | 0 | Non-SCC | G/G | G/G | G/G |
| NSCLC-0191 | 1 | 75 | 1 | 0 | 1 | Non-SCC | G/G | G/G | A/A |
| NSCLC-0192 | 2 | 53 | 0 | 0 | 1 | Non-SCC | G/A | G/A | G/A |
| NSCLC-0193 | 2 | 46 | 0 | 0 | 0 | Non-SCC | G/A | G/A | G/A |
| NSCLC-0194 | 1 | 48 | 1 | 0 | 0 | Non-SCC | G/A | G/A | G/A |
| NSCLC-0195 | 2 | 56 | 0 | 0 | 1 | Non-SCC | G/G | G/G | G/G |
| NSCLC-0196 | 2 | 43 | 0 | 0 | 0 | Non-SCC | G/G | G/G | G/G |
| NSCLC-0197 | 2 | 53 | 0 | 0 | 0 | Non-SCC | G/G | G/G | G/A |
| NSCLC-0198 | 2 | 62 | 0 | 0 | 1 | Non-SCC | G/A | G/G | G/G |
| NSCLC-0199 | 1 | 67 | 1 | 0 | 0 | Non-SCC | G/G | G/G | G/G |
| NSCLC-0200 | 1 | 53 | 0 | 0 | 1 | Non-SCC | G/G | G/G | G/G |
| NSCLC-0201 | 2 | 47 | 0 | 0 | 0 | Non-SCC | G/G | G/G | G/G |
| NSCLC-0202 | 2 | 49 | 0 | 0 | 1 | Non-SCC | G/G | G/G | G/G |
| NSCLC-0203 | 2 | 64 | 0 | 0 | 1 | Non-SCC | G/G | G/G | G/G |
| NSCLC-0204 | 2 | 57 | 0 | 0 | 0 | Non-SCC | G/G | G/G | G/G |
| NSCLC-0205 | 2 | 62 | 0 | 0 | 0 | Non-SCC | G/G | G/G | G/G |
| NSCLC-0206 | 2 | 53 | 0 | 0 | 0 | Non-SCC | G/G | G/G | G/G |
| NSCLC-0207 | 2 | 64 | 0 | 0 | 0 | Non-SCC | G/A | G/G | G/G |
| NSCLC-0208 | 2 | 59 | 0 | 0 | 0 | Non-SCC | G/A | G/A | G/A |
| NSCLC-0209 | 2 | 52 | 0 | 0 | 1 | Non-SCC | G/G | G/G | G/G |
| NSCLC-0210 | 2 | 55 | 0 | 0 | 1 | Non-SCC | G/G | G/G | G/G |
| NSCLC-0211 | 2 | 63 | 0 | 0 | 0 | Non-SCC | G/G | G/G | G/G |
| NSCLC-0212 | 1 | 42 | 1 | 0 | 0 | Non-SCC | G/A | G/A | A/A |
| NSCLC-0213 | 1 | 56 | 1 | 1 | 1 | Non-SCC | G/G | G/G | G/G |
| NSCLC-0214 | 1 | 61 | 1 | 0 | 0 | Non-SCC | G/G | G/G | G/G |
| NSCLC-0215 | 2 | 39 | 0 | 0 | 0 | Non-SCC | G/A | G/A | G/A |
| NSCLC-0216 | 1 | 65 | 1 | 0 | 1 | Non-SCC | G/G | G/G | G/G |
| NSCLC-0217 | 1 | 48 | 1 | 1 | 0 | Non-SCC | G/G | G/G | G/G |
| NSCLC-0218 | 2 | 54 | 0 | 0 | 0 | Non-SCC | G/G | G/G | G/G |
| NSCLC-0219 | 1 | 59 | 1 | 0 | 1 | Non-SCC | G/A | G/A | G/A |
| NSCLC-0220 | 1 | 52 | 1 | 0 | 0 | Non-SCC | G/G | G/G | G/G |
| NSCLC-0221 | 2 | 61 | 0 | 0 | 0 | Non-SCC | G/G | G/G | G/G |
| NSCLC-0222 | 2 | 59 | 0 | 0 | 1 | Non-SCC | G/A | G/A | A/A |
| NSCLC-0223 | 2 | 64 | 0 | 0 | 0 | Non-SCC | G/A | G/A | G/A |
| NSCLC-0224 | 1 | 51 | 1 | 0 | 0 | Non-SCC | G/G | G/G | G/G |
| NSCLC-0225 | 1 | 73 | 0 | 0 | 1 | Non-SCC | G/G | G/G | G/G |
| NSCLC-0226 | 1 | 76 | 0 | 0 | 0 | Non-SCC | G/G | G/G | G/G |
| NSCLC-0227 | 2 | 70 | 0 | 0 | 0 | Non-SCC | G/G | G/G | G/G |
| NSCLC-0228 | 1 | 67 | 0 | 0 | 0 | Non-SCC | G/G | G/G | G/G |
| NSCLC-0229 | 2 | 65 | 0 | 0 | 0 | Non-SCC | G/G | G/G | G/G |
| NSCLC-0230 | 1 | 44 | 1 | 1 | 0 | Non-SCC | G/A | G/G | G/G |
| NSCLC-0231 | 2 | 72 | 0 | 0 | 1 | Non-SCC | G/G | G/G | G/G |
| NSCLC-0232 | 2 | 48 | 0 | 0 | 1 | Non-SCC | G/G | G/G | G/G |
| NSCLC-0233 | 2 | 65 | 0 | 0 | 0 | Non-SCC | G/A | G/A | G/G |
| NSCLC-0234 | 2 | 53 | 0 | 0 | 0 | Non-SCC | G/G | G/G | G/G |
| NSCLC-0235 | 1 | 68 | 1 | 0 | 0 | Non-SCC | G/G | G/G | G/G |
| NSCLC-0236 | 1 | 53 | 1 | 0 | 0 | Non-SCC | G/G | G/G | G/G |
| NSCLC-0237 | 1 | 53 | 1 | 0 | 0 | Non-SCC | G/G | G/G | G/G |
| NSCLC-0238 | 1 | 59 | 1 | 0 | 0 | Non-SCC | G/A | G/A | A/A |
| NSCLC-0239 | 1 | 59 | 1 | 0 | 1 | Non-SCC | G/G | G/G | G/G |
| NSCLC-0240 | 2 | 59 | 0 | 0 | 1 | Non-SCC | G/G | G/G | G/A |
| NSCLC-0241 | 1 | 78 | 1 | 0 | 1 | Non-SCC | G/G | G/G | G/G |
| NSCLC-0242 | 1 | 61 | 0 | 0 | 0 | Non-SCC | G/A | G/G | G/G |
| NSCLC-0243 | 1 | 53 | 0 | 0 | 0 | Non-SCC | G/G | G/G | G/G |
| NSCLC-0244 | 2 | 74 | 0 | 0 | 1 | Non-SCC | G/A | G/A | G/G |
| NSCLC-0245 | 2 | 73 | 0 | 0 | 0 | Non-SCC | G/G | G/G | G/G |
| NSCLC-0246 | 2 | 61 | 0 | 0 | 0 | Non-SCC | G/G | G/G | G/G |
| NSCLC-0247 | 1 | 60 | 1 | 0 | 0 | Non-SCC | G/G | G/G | G/G |
| NSCLC-0248 | 1 | 70 | 1 | 1 | 1 | Non-SCC | G/G | G/G | G/A |
| NSCLC-0249 | 1 | 80 | 0 | 0 | 1 | Non-SCC | G/G | G/G | G/G |
| NSCLC-0250 | 1 | 62 | 1 | 1 | 0 | Non-SCC | G/G | G/G | G/A |
| NSCLC-0251 | 2 | 64 | 0 | 0 | 1 | Non-SCC | G/G | G/A | G/G |
| NSCLC-0252 | 2 | 45 | 0 | 0 | 1 | Non-SCC | G/G | G/G | G/G |
| NSCLC-0253 | 2 | 67 | 0 | 0 | 0 | Non-SCC | G/G | G/G | G/G |
| NSCLC-0254 | 2 | 53 | 0 | 0 | 0 | Non-SCC | G/G | G/G | G/G |
| NSCLC-0255 | 2 | 57 | 0 | 0 | 0 | Non-SCC | G/G | G/G | G/G |
| NSCLC-0256 | 2 | 62 | 0 | 0 | 0 | Non-SCC | G/G | G/G | G/G |
| NSCLC-0257 | 2 | 66 | 0 | 0 | 1 | Non-SCC | G/G | G/G | G/G |
| NSCLC-0258 | 1 | 49 | 0 | 0 | 1 | Non-SCC | G/G | G/G | G/A |
| NSCLC-0259 | 1 | 70 | 1 | 0 | 0 | Non-SCC | G/G | G/G | G/A |
| NSCLC-0260 | 1 | 71 | 1 | 0 | 0 | Non-SCC | G/G | G/G | G/G |
| NSCLC-0261 | 1 | 47 | 1 | 0 | 0 | Non-SCC | G/G | G/G | G/G |
| NSCLC-0262 | 1 | 64 | 1 | 0 | 0 | Non-SCC | G/A | G/A | G/A |
| NSCLC-0263 | 2 | 55 | 0 | 0 | 1 | Non-SCC | G/A | G/G | G/G |
| NSCLC-0264 | 2 | 63 | 0 | 0 | 0 | Non-SCC | G/G | G/G | G/G |
| NSCLC-0265 | 2 | 64 | 0 | 0 | 0 | Non-SCC | G/G | G/G | G/G |
| NSCLC-0266 | 1 | 70 | 1 | 0 | 1 | Non-SCC | G/G | G/G | G/A |
| NSCLC-0267 | 1 | 64 | 0 | 0 | 0 | Non-SCC | G/G | G/G | G/G |
| NSCLC-0268 | 1 | 62 | 1 | 0 | 1 | Non-SCC | G/G | G/G | G/A |
| NSCLC-0269 | 2 | 72 | 0 | 0 | 0 | Non-SCC | G/G | G/G | G/G |
| NSCLC-0270 | 1 | 59 | 0 | 0 | 1 | Non-SCC | G/G | G/G | G/G |
| NSCLC-0271 | 1 | 73 | 1 | 0 | 0 | Non-SCC | G/G | G/G | G/A |
| NSCLC-0272 | 2 | 68 | 0 | 0 | 1 | Non-SCC | G/A | G/G | G/G |
| NSCLC-0273 | 1 | 44 | 1 | 0 | 0 | Non-SCC | G/A | G/A | G/G |
| NSCLC-0274 | 2 | 48 | 0 | 0 | 1 | Non-SCC | G/G | G/G | G/G |
| NSCLC-0275 | 1 | 87 | 0 | 0 | 1 | Non-SCC | G/G | G/A | G/G |
| NSCLC-0276 | 2 | 60 | 0 | 0 | 1 | Non-SCC | G/A | G/A | G/G |
| NSCLC-0277 | 2 | 58 | 0 | 0 | 0 | Non-SCC | G/A | G/A | G/G |
| NSCLC-0278 | 2 | 61 | 0 | 0 | 0 | Non-SCC | G/G | G/G | G/A |
| NSCLC-0279 | 2 | 57 | 0 | 0 | 0 | Non-SCC | G/A | G/G | G/A |
| NSCLC-0280 | 1 | 50 | 0 | 0 | 1 | Non-SCC | G/G | G/G | G/G |
| NSCLC-0281 | 2 | 69 | 0 | 0 | 0 | Non-SCC | G/G | G/A | G/G |
| NSCLC-0282 | 1 | 58 | 1 | 0 | 0 | Non-SCC | G/G | G/G | G/G |
| NSCLC-0283 | 2 | 52 | 0 | 0 | 0 | Non-SCC | G/G | G/G | G/G |
| NSCLC-0284 | 2 | 56 | 0 | 0 | 1 | Non-SCC | G/G | G/G | G/G |
| NSCLC-0285 | 1 | 34 | 1 | 0 | 1 | Non-SCC | G/A | G/G | G/G |
| NSCLC-0286 | 2 | 70 | 0 | 0 | 0 | Non-SCC | G/G | G/G | G/A |
| NSCLC-0287 | 2 | 36 | 0 | 0 | 0 | Non-SCC | G/G | G/G | G/G |
| NSCLC-0288 | 2 | 65 | 0 | 0 | 1 | Non-SCC | G/G | G/G | G/G |
| NSCLC-0289 | 2 | 40 | 0 | 0 | 1 | Non-SCC | G/A | G/A | G/G |
| NSCLC-0290 | 2 | 42 | 0 | 0 | 0 | Non-SCC | G/G | G/G | G/G |
| NSCLC-0291 | 1 | 62 | 0 | 0 | 1 | Non-SCC | G/G | G/G | G/G |
| NSCLC-0292 | 2 | 58 | 0 | 0 | 0 | Non-SCC | G/G | G/G | G/G |
| NSCLC-0293 | 1 | 53 | 1 | 0 | 0 | Non-SCC | G/G | G/A | G/G |
| NSCLC-0294 | 1 | 66 | 1 | 0 | 1 | Non-SCC | G/A | G/A | G/G |
| NSCLC-0295 | 1 | 58 | 1 | 1 | 0 | Non-SCC | G/A | G/G | G/G |
| NSCLC-0296 | 2 | 57 | 0 | 0 | 1 | Non-SCC | G/A | G/G | G/A |
| NSCLC-0297 | 2 | 73 | 0 | 0 | 1 | Non-SCC | G/A | G/A | G/A |
| NSCLC-0298 | 2 | 61 | 0 | 0 | 0 | Non-SCC | G/A | G/A | G/A |
| NSCLC-0299 | 2 | 63 | 0 | 0 | 1 | Non-SCC | G/G | G/A | G/G |
| NSCLC-0300 | 2 | 60 | 0 | 0 | 1 | Non-SCC | G/G | G/G | G/G |
| NSCLC-0301 | 2 | 67 | 0 | 0 | 0 | Non-SCC | G/G | G/G | G/A |
| NSCLC-0302 | 2 | 64 | 0 | 0 | 0 | Non-SCC | G/G | G/G | G/A |
| NSCLC-0303 | 2 | 70 | 0 | 0 | 1 | Non-SCC | G/A | G/G | G/G |
| NSCLC-0304 | 1 | 87 | 0 | 0 | 1 | Non-SCC | G/G | G/A | G/G |
| NSCLC-0305 | 1 | 66 | 1 | 0 | 1 | Non-SCC | G/G | G/G | G/G |
| NSCLC-0306 | 1 | 46 | 1 | 0 | 0 | Non-SCC | G/G | G/G | G/G |
| NSCLC-0307 | 2 | 59 | 0 | 0 | 1 | Non-SCC | G/A | G/A | G/A |
| NSCLC-0308 | 2 | 58 | 0 | 0 | 0 | Non-SCC | G/G | G/G | G/G |
| NSCLC-0309 | 1 | 46 | 0 | 0 | 0 | Non-SCC | G/G | G/G | G/G |
| NSCLC-0310 | 1 | 62 | 1 | 0 | 0 | Non-SCC | G/G | G/G | G/G |
| NSCLC-0311 | 1 | 76 | 0 | 0 | 1 | Non-SCC | G/G | G/G | G/G |
| NSCLC-0312 | 2 | 46 | 0 | 0 | 1 | Non-SCC | G/A | G/A | G/A |
| NSCLC-0313 | 2 | 78 | 0 | 0 | 0 | Non-SCC | G/G | G/G | G/G |
| NSCLC-0314 | 2 | 51 | 0 | 0 | 0 | Non-SCC | G/A | G/A | A/A |
| NSCLC-0315 | 1 | 60 | 1 | 0 | 0 | Non-SCC | G/G | G/G | G/G |
| NSCLC-0316 | 1 | 43 | 0 | 0 | 0 | Non-SCC | G/G | G/G | G/G |
| NSCLC-0317 | 1 | 53 | 1 | 0 | 1 | Non-SCC | G/G | G/G | G/G |
| NSCLC-0318 | 1 | 52 | 1 | 0 | 0 | Non-SCC | G/A | A/A | G/A |
| NSCLC-0319 | 1 | 58 | 0 | 1 | 0 | Non-SCC | G/G | G/G | G/G |
| NSCLC-0320 | 1 | 60 | 0 | 0 | 1 | Non-SCC | G/G | G/G | G/G |
| NSCLC-0321 | 2 | 53 | 0 | 0 | 1 | Non-SCC | G/A | G/A | G/G |
| NSCLC-0322 | 2 | 81 | 0 | 0 | 0 | Non-SCC | G/A | G/G | G/A |
| NSCLC-0323 | 2 | 66 | 0 | 0 | 0 | Non-SCC | G/G | G/G | G/G |
| NSCLC-0324 | 2 | 62 | 0 | 0 | 0 | Non-SCC | A/A | A/A | A/A |
| NSCLC-0325 | 2 | 48 | 0 | 0 | 0 | Non-SCC | G/A | G/G | G/G |
| NSCLC-0326 | 2 | 39 | 0 | 0 | 0 | Non-SCC | G/G | G/G | G/G |
| NSCLC-0327 | 2 | 39 | 0 | 0 | 0 | Non-SCC | G/G | G/G | G/G |
| NSCLC-0328 | 1 | 66 | 1 | 0 | 0 | Non-SCC | G/G | G/G | G/G |
| NSCLC-0329 | 1 | 37 | 0 | 0 | 1 | Non-SCC | G/G | G/G | G/G |
| NSCLC-0330 | 1 | 61 | 1 | 0 | 0 | Non-SCC | G/G | G/G | G/G |
| NSCLC-0331 | 1 | 43 | 1 | 1 | 1 | Non-SCC | G/A | G/G | G/G |
| NSCLC-0332 | 1 | 73 | 1 | 0 | 0 | Non-SCC | G/G | G/G | G/G |
| NSCLC-0333 | 1 | 61 | 1 | 0 | 0 | Non-SCC | G/G | G/G | G/G |
| NSCLC-0334 | 1 | 76 | 0 | 0 | 1 | Non-SCC | G/A | G/G | G/G |
| NSCLC-0335 | 2 | 68 | 0 | 0 | 0 | Non-SCC | G/G | G/G | G/G |
| NSCLC-0336 | 2 | 63 | 0 | 0 | 0 | Non-SCC | G/G | G/G | G/G |
| NSCLC-0337 | 1 | 52 | 1 | 0 | 0 | Non-SCC | G/G | G/G | G/G |
| NSCLC-0338 | 1 | 74 | 1 | 0 | 0 | Non-SCC | G/G | G/G | G/G |
| NSCLC-0339 | 1 | 43 | 1 | 0 | 1 | Non-SCC | G/G | G/G | G/G |
| NSCLC-0340 | 1 | 61 | 1 | 0 | 1 | Non-SCC | G/G | G/G | G/G |
| NSCLC-0341 | 2 | 75 | 0 | 0 | 1 | Non-SCC | G/A | G/G | G/G |
| NSCLC-0342 | 2 | 53 | 0 | 0 | 0 | Non-SCC | G/A | G/G | G/G |
| NSCLC-0343 | 2 | 38 | 0 | 0 | 0 | Non-SCC | G/A | G/A | G/A |
| NSCLC-0344 | 2 | 68 | 0 | 0 | 0 | Non-SCC | G/G | G/G | G/A |
| NSCLC-0345 | 2 | 28 | 0 | 0 | 0 | Non-SCC | G/G | G/G | G/G |
| NSCLC-0346 | 1 | 56 | 1 | 0 | 0 | Non-SCC | G/G | G/G | G/G |
| NSCLC-0347 | 2 | 68 | 0 | 0 | 1 | Non-SCC | G/G | G/G | G/G |
| NSCLC-0348 | 1 | 63 | 1 | 0 | 1 | Non-SCC | G/G | G/G | G/G |
| NSCLC-0349 | 2 | 50 | 0 | 0 | 1 | Non-SCC | G/G | G/G | G/G |
| NSCLC-0350 | 2 | 50 | 0 | 0 | 1 | Non-SCC | G/A | G/G | G/A |
| NSCLC-0351 | 2 | 70 | 0 | 0 | 0 | Non-SCC | G/G | G/G | G/G |
| NSCLC-0352 | 2 | 46 | 0 | 0 | 0 | Non-SCC | G/A | G/G | G/A |
| NSCLC-0353 | 1 | 62 | 1 | 0 | 0 | Non-SCC | G/G | G/G | G/G |
| NSCLC-0354 | 1 | 44 | 1 | 0 | 1 | Non-SCC | G/G | G/G | G/G |
| NSCLC-0355 | 2 | 55 | 0 | 0 | 0 | Non-SCC | G/A | G/G | G/A |
| NSCLC-0356 | 1 | 61 | 1 | 0 | 0 | Non-SCC | G/G | G/G | G/G |
| NSCLC-0357 | 1 | 50 | 1 | 0 | 0 | Non-SCC | G/A | G/A | G/A |
| NSCLC-0358 | 2 | 61 | 0 | 0 | 0 | Non-SCC | G/A | G/G | G/A |
| NSCLC-0359 | 1 | 58 | 1 | 0 | 1 | Non-SCC | G/G | G/G | G/G |
| NSCLC-0360 | 2 | 76 | 0 | 0 | 1 | Non-SCC | G/G | G/G | G/G |
| NSCLC-0361 | 2 | 63 | 0 | 0 | 0 | Non-SCC | G/G | G/G | G/G |
| NSCLC-0362 | 1 | 59 | 0 | 0 | 0 | Non-SCC | G/A | G/G | G/G |
| NSCLC-0363 | 1 | 70 | 1 | 0 | 1 | Non-SCC | G/G | G/G | G/G |
| NSCLC-0364 | 2 | 57 | 0 | 0 | 1 | Non-SCC | G/A | G/A | G/A |
| NSCLC-0365 | 1 | 56 | 1 | 0 | 0 | Non-SCC | G/G | G/G | G/G |
| NSCLC-0366 | 1 | 52 | 1 | 0 | 1 | Non-SCC | G/G | G/G | G/G |
| NSCLC-0367 | 2 | 50 | 0 | 0 | 0 | Non-SCC | G/G | G/G | G/G |
| NSCLC-0368 | 2 | 62 | 0 | 0 | 0 | Non-SCC | G/G | G/G | G/G |
| NSCLC-0369 | 1 | 51 | 1 | 0 | 1 | Non-SCC | G/G | G/G | G/A |
| NSCLC-0370 | 2 | 45 | 0 | 0 | 0 | Non-SCC | A/A | A/A | G/A |
| NSCLC-0371 | 1 | 60 | 1 | 0 | 1 | Non-SCC | G/G | G/G | G/G |
| NSCLC-0372 | 1 | 53 | 0 | 0 | 1 | Non-SCC | G/A | G/G | G/G |
| NSCLC-0373 | 1 | 59 | 0 | 0 | 1 | Non-SCC | G/G | G/G | G/A |
| NSCLC-0374 | 2 | 52 | 0 | 0 | 0 | Non-SCC | G/A | G/A | G/A |
| NSCLC-0375 | 2 | 52 | 0 | 0 | 1 | Non-SCC | G/G | G/G | G/G |
| NSCLC-0376 | 1 | 61 | 1 | 0 | 0 | Non-SCC | G/A | G/A | G/A |
| NSCLC-0377 | 1 | 44 | 1 | 0 | 0 | Non-SCC | G/G | G/G | G/G |
| NSCLC-0378 | 2 | 60 | 0 | 0 | 1 | Non-SCC | G/G | G/G | G/A |
| NSCLC-0379 | 1 | 59 | 0 | 0 | 0 | Non-SCC | G/G | G/G | G/G |
| NSCLC-0380 | 1 | 52 | 1 | 0 | 1 | Non-SCC | G/G | G/G | G/G |
| NSCLC-0381 | 1 | 67 | 1 | 1 | 1 | Non-SCC | G/A | G/A | G/A |
| NSCLC-0382 | 1 | 75 | 0 | 0 | 0 | Non-SCC | G/A | G/A | G/A |
| NSCLC-0383 | 2 | 63 | 0 | 0 | 1 | Non-SCC | G/G | G/G | G/G |
| NSCLC-0384 | 2 | 49 | 0 | 0 | 1 | Non-SCC | G/G | G/G | G/A |
| NSCLC-0385 | 2 | 36 | 0 | 0 | 0 | Non-SCC | G/G | G/G | G/A |
| NSCLC-0386 | 2 | 56 | 0 | 0 | 0 | Non-SCC | G/G | G/A | G/G |
| NSCLC-0387 | 2 | 65 | 0 | 0 | 0 | Non-SCC | G/G | G/G | G/A |
| NSCLC-0388 | 1 | 48 | 1 | 0 | 1 | Non-SCC | G/G | G/G | G/G |
| NSCLC-0389 | 2 | 49 | 0 | 0 | 0 | Non-SCC | G/G | G/G | G/G |
| NSCLC-0390 | 2 | 60 | 0 | 0 | 0 | Non-SCC | G/G | G/G | G/G |
| NSCLC-0391 | 1 | 69 | 1 | 0 | 0 | Non-SCC | G/G | G/G | G/G |
| NSCLC-0392 | 2 | 59 | 0 | 0 | 0 | Non-SCC | G/A | G/A | G/G |
| NSCLC-0393 | 1 | 76 | 1 | 0 | 0 | Non-SCC | G/G | G/G | G/G |
| NSCLC-0394 | 1 | 63 | 1 | 1 | 0 | Non-SCC | G/G | G/A | G/G |
| NSCLC-0395 | 2 | 60 | 0 | 0 | 0 | Non-SCC | G/G | G/G | G/G |
| NSCLC-0396 | 2 | 59 | 0 | 0 | 0 | Non-SCC | G/G | G/G | G/G |
| NSCLC-0397 | 2 | 35 | 0 | 0 | 0 | Non-SCC | G/G | G/G | G/G |
| NSCLC-0398 | 2 | 64 | 0 | 0 | 1 | Non-SCC | G/A | G/A | G/G |
| NSCLC-0399 | 2 | 53 | 0 | 0 | 1 | Non-SCC | G/G | G/G | G/G |
| NSCLC-0400 | 2 | 62 | 0 | 0 | 0 | Non-SCC | G/G | G/G | G/G |
| NSCLC-0401 | 1 | 69 | 0 | 0 | 0 | Non-SCC | G/G | G/G | G/G |
| NSCLC-0402 | 1 | 40 | 1 | 0 | 1 | Non-SCC | G/G | G/A | G/G |
| NSCLC-0403 | 1 | 45 | 0 | 0 | 1 | Non-SCC | G/A | G/G | G/A |
| NSCLC-0404 | 2 | 52 | 0 | 0 | 1 | Non-SCC | G/G | G/G | G/G |
| NSCLC-0405 | 1 | 46 | 1 | 0 | 1 | Non-SCC | G/G | G/G | G/G |
| NSCLC-0406 | 2 | 44 | 0 | 0 | 1 | Non-SCC | G/A | G/G | G/G |
| NSCLC-0407 | 1 | 49 | 1 | 1 | 0 | Non-SCC | G/A | G/A | G/A |
| NSCLC-0408 | 1 | 58 | 0 | 0 | 0 | Non-SCC | G/G | G/G | G/G |
| NSCLC-0409 | 2 | 52 | 0 | 0 | 1 | Non-SCC | G/A | G/G | G/G |
| NSCLC-0410 | 2 | 67 | 0 | 0 | 0 | Non-SCC | G/A | G/A | G/A |
| NSCLC-0411 | 1 | 52 | 1 | 1 | 0 | Non-SCC | G/G | G/G | G/G |
| NSCLC-0412 | 2 | 67 | 0 | 0 | 0 | Non-SCC | G/G | G/G | G/G |
| NSCLC-0413 | 2 | 67 | 0 | 0 | 0 | Non-SCC | G/A | G/A | G/G |
| NSCLC-0414 | 1 | 59 | 1 | 0 | 0 | Non-SCC | G/G | G/G | G/G |
| NSCLC-0415 | 2 | 63 | 0 | 0 | 0 | Non-SCC | A/A | G/G | A/A |
| NSCLC-0416 | 1 | 54 | 1 | 0 | 1 | Non-SCC | G/G | G/G | G/G |
| NSCLC-0417 | 2 | 61 | 0 | 0 | 0 | Non-SCC | G/A | G/A | G/A |
| NSCLC-0418 | 1 | 57 | 0 | 0 | 1 | Non-SCC | G/G | G/G | G/G |
| NSCLC-0419 | 2 | 69 | 0 | 0 | 0 | Non-SCC | G/G | G/G | G/G |
| NSCLC-0420 | 1 | 67 | 0 | 0 | 0 | Non-SCC | G/G | G/G | G/G |
| NSCLC-0421 | 2 | 60 | 0 | 0 | 0 | Non-SCC | G/G | G/G | G/G |
| NSCLC-0422 | 2 | 72 | 0 | 0 | 0 | Non-SCC | G/A | G/A | G/A |
| NSCLC-0423 | 1 | 70 | 0 | 0 | 1 | Non-SCC | G/G | G/G | G/G |
| NSCLC-0424 | 2 | 46 | 0 | 0 | 0 | Non-SCC | G/G | G/G | G/G |
| NSCLC-0425 | 1 | 61 | 0 | 0 | 1 | Non-SCC | G/G | G/G | G/G |
| NSCLC-0426 | 1 | 62 | 1 | 1 | 0 | Non-SCC | G/G | G/G | G/G |
| NSCLC-0427 | 1 | 65 | 1 | 0 | 1 | Non-SCC | G/G | G/G | G/G |
| NSCLC-0428 | 1 | 77 | 1 | 1 | 0 | Non-SCC | G/G | G/G | G/G |
| NSCLC-0429 | 2 | 38 | 0 | 0 | 0 | Non-SCC | G/G | G/G | G/G |
| NSCLC-0430 | 2 | 44 | 0 | 0 | 0 | Non-SCC | G/A | G/A | G/A |
| NSCLC-0431 | 1 | 60 | 0 | 1 | 1 | Non-SCC | G/A | G/G | G/A |
| NSCLC-0432 | 2 | 43 | 0 | 0 | 1 | Non-SCC | G/G | G/G | G/G |
| NSCLC-0433 | 2 | 57 | 0 | 0 | 0 | Non-SCC | G/A | G/G | G/G |
| NSCLC-0434 | 2 | 69 | 0 | 0 | 0 | Non-SCC | G/G | G/G | G/G |
| NSCLC-0435 | 2 | 65 | 0 | 0 | 1 | Non-SCC | G/G | G/G | G/A |
| NSCLC-0436 | 2 | 62 | 0 | 0 | 1 | Non-SCC | A/A | G/A | G/A |
| NSCLC-0437 | 1 | 58 | 1 | 0 | 0 | Non-SCC | G/G | G/G | G/G |
| NSCLC-0438 | 1 | 74 | 1 | 0 | 1 | Non-SCC | G/G | G/G | G/G |
| NSCLC-0439 | 1 | 56 | 1 | 1 | 1 | Non-SCC | G/G | G/G | G/G |
| NSCLC-0440 | 2 | 60 | 0 | 0 | 0 | Non-SCC | G/A | G/A | G/A |
| NSCLC-0441 | 1 | 27 | 0 | 1 | 0 | Non-SCC | G/G | G/G | G/G |
| NSCLC-0442 | 1 | 48 | 1 | 0 | 0 | Non-SCC | G/G | G/G | G/G |
| NSCLC-0443 | 2 | 73 | 0 | 0 | 0 | Non-SCC | G/G | G/G | G/G |
| NSCLC-0444 | 2 | 59 | 0 | 0 | 1 | Non-SCC | G/G | G/G | G/G |
| NSCLC-0445 | 1 | 64 | 0 | 0 | 0 | Non-SCC | G/A | G/A | G/G |
| NSCLC-0446 | 2 | 59 | 0 | 0 | 0 | Non-SCC | G/G | G/G | G/A |
| NSCLC-0447 | 1 | 63 | 1 | 1 | 0 | Non-SCC | G/G | G/G | G/G |
| NSCLC-0448 | 2 | 59 | 0 | 0 | 0 | Non-SCC | G/G | G/G | G/G |
| NSCLC-0449 | 2 | 60 | 0 | 0 | 0 | Non-SCC | A/A | G/A | A/A |
| NSCLC-0450 | 1 | 62 | 1 | 1 | 0 | Non-SCC | G/G | G/G | G/G |
| NSCLC-0451 | 2 | 66 | 0 | 0 | 1 | Non-SCC | G/A | G/A | G/A |
| NSCLC-0452 | 2 | 74 | 0 | 0 | 0 | Non-SCC | G/A | G/A | G/G |
| NSCLC-0453 | 1 | 65 | 1 | 1 | 0 | Non-SCC | G/G | G/G | G/G |
| NSCLC-0454 | 1 | 63 | 0 | 0 | 1 | Non-SCC | G/G | G/G | G/G |
| NSCLC-0455 | 2 | 67 | 0 | 0 | 1 | Non-SCC | G/A | G/A | G/A |
| NSCLC-0456 | 1 | 52 | 1 | 0 | 0 | Non-SCC | G/A | G/G | G/A |
| NSCLC-0457 | 2 | 61 | 0 | 0 | 0 | Non-SCC | G/G | G/G | G/A |
| NSCLC-0458 | 1 | 67 | 0 | 0 | 0 | Non-SCC | G/G | G/G | G/G |
| NSCLC-0459 | 1 | 72 | 0 | 0 | 0 | Non-SCC | G/G | G/G | G/A |
| NSCLC-0460 | 1 | 70 | 1 | 1 | 1 | Non-SCC | G/A | G/G | G/A |
| NSCLC-0461 | 2 | 59 | 0 | 0 | 0 | Non-SCC | G/G | G/G | G/G |
| NSCLC-0462 | 1 | 56 | 0 | 1 | 1 | Non-SCC | G/A | G/A | G/A |
| NSCLC-0463 | 1 | 76 | 1 | 1 | 0 | Non-SCC | G/G | G/G | G/G |
| NSCLC-0464 | 1 | 74 | 0 | 0 | 0 | Non-SCC | G/G | G/G | G/G |
| NSCLC-0465 | 2 | 26 | 0 | 0 | 1 | Non-SCC | G/G | G/G | G/G |
| NSCLC-0466 | 2 | 56 | 0 | 0 | 1 | Non-SCC | G/G | G/G | G/G |
| NSCLC-0467 | 1 | 48 | 0 | 0 | 1 | Non-SCC | G/G | G/G | G/G |
| NSCLC-0468 | 2 | 65 | 0 | 0 | 0 | Non-SCC | G/G | G/G | G/G |
| NSCLC-0469 | 2 | 51 | 0 | 0 | 0 | Non-SCC | G/G | G/G | G/G |
| NSCLC-0470 | 1 | 58 | 1 | 1 | 1 | Non-SCC | G/G | G/G | G/G |
| NSCLC-0471 | 2 | 44 | 0 | 0 | 1 | Non-SCC | G/G | G/G | G/G |
| NSCLC-0472 | 1 | 73 | 0 | 0 | 1 | Non-SCC | G/G | G/G | G/A |
| NSCLC-0473 | 2 | 70 | 0 | 0 | 1 | Non-SCC | G/G | G/G | G/G |
| NSCLC-0474 | 1 | 64 | 1 | 0 | 0 | Non-SCC | G/G | G/G | G/G |
| NSCLC-0475 | 2 | 49 | 0 | 0 | 0 | Non-SCC | G/G | G/G | G/G |
| NSCLC-0476 | 1 | 42 | 0 | 1 | 1 | Non-SCC | G/G | G/G | G/A |
| NSCLC-0477 | 1 | 79 | 0 | 0 | 1 | Non-SCC | G/A | G/G | G/A |
| NSCLC-0478 | 1 | 52 | 1 | 0 | 0 | Non-SCC | G/A | G/A | G/G |
| NSCLC-0479 | 2 | 41 | 0 | 0 | 0 | Non-SCC | G/G | G/G | G/G |
| NSCLC-0480 | 1 | 40 | 0 | 1 | 0 | Non-SCC | G/G | G/G | G/G |
| NSCLC-0481 | 2 | 61 | 0 | 0 | 1 | Non-SCC | G/G | G/G | G/G |
| NSCLC-0482 | 1 | 63 | 1 | 1 | 1 | Non-SCC | G/G | G/G | G/G |
| NSCLC-0483 | 1 | 62 | 1 | 1 | 0 | Non-SCC | G/G | G/G | G/G |
| NSCLC-0484 | 2 | 40 | 0 | 0 | 1 | Non-SCC | G/A | G/G | G/G |
| NSCLC-0485 | 2 | 49 | 0 | 0 | 0 | Non-SCC | G/G | G/G | G/G |
| NSCLC-0486 | 2 | 44 | 0 | 0 | 0 | Non-SCC | G/A | G/A | A/A |
| NSCLC-0487 | 2 | 54 | 0 | 0 | 1 | Non-SCC | G/G | G/G | G/A |
| NSCLC-0488 | 2 | 61 | 0 | 0 | 1 | Non-SCC | G/G | G/G | G/G |
| NSCLC-0489 | 2 | 74 | 0 | 0 | 0 | Non-SCC | G/A | G/G | G/G |
| NSCLC-0490 | 1 | 59 | 1 | 1 | 1 | Non-SCC | G/G | G/G | G/A |
| NSCLC-0491 | 1 | 68 | 0 | 0 | 0 | Non-SCC | G/A | G/A | G/A |
| NSCLC-0492 | 1 | 72 | 1 | 0 | 1 | Non-SCC | G/A | G/G | G/A |
| NSCLC-0493 | 1 | 52 | 1 | 1 | 1 | Non-SCC | G/A | G/G | G/G |
| NSCLC-0494 | 1 | 59 | 1 | 1 | 1 | Non-SCC | G/A | G/G | G/G |
| NSCLC-0495 | 2 | 42 | 0 | 0 | 0 | Non-SCC | G/G | G/G | G/G |
| NSCLC-0496 | 2 | 62 | 0 | 0 | 0 | Non-SCC | G/A | G/A | G/G |
| NSCLC-0497 | 2 | 28 | 0 | 0 | 0 | Non-SCC | G/A | G/A | G/A |
| NSCLC-0498 | 2 | 66 | 0 | 0 | 1 | Non-SCC | G/A | G/A | G/G |
| NSCLC-0499 | 2 | 45 | 0 | 0 | 0 | Non-SCC | G/G | G/G | G/A |
| NSCLC-0500 | 1 | 41 | 0 | 0 | 0 | Non-SCC | G/G | G/G | G/G |
| NSCLC-0501 | 1 | 66 | 1 | 1 | 0 | Non-SCC | G/G | G/G | G/G |
| NSCLC-0502 | 2 | 57 | 0 | 0 | 0 | Non-SCC | G/G | G/G | G/G |
| NSCLC-0503 | 1 | 72 | 1 | 0 | 1 | Non-SCC | G/G | G/G | G/A |
| NSCLC-0504 | 1 | 66 | 0 | 0 | 0 | Non-SCC | G/G | G/G | G/G |
| NSCLC-0505 | 2 | 46 | 0 | 0 | 1 | Non-SCC | G/A | G/A | G/A |
| NSCLC-0506 | 1 | 66 | 0 | 0 | 1 | Non-SCC | G/G | G/G | G/G |
| NSCLC-0507 | 1 | 70 | 0 | 0 | 1 | Non-SCC | G/G | G/G | G/G |
| NSCLC-0508 | 1 | 48 | 1 | 0 | 0 | Non-SCC | G/G | G/G | G/G |
| NSCLC-0509 | 2 | 57 | 0 | 0 | 0 | Non-SCC | G/A | G/G | G/A |
| NSCLC-0510 | 1 | 64 | 1 | 0 | 0 | Non-SCC | G/G | G/G | G/G |
| NSCLC-0511 | 2 | 53 | 0 | 0 | 0 | Non-SCC | G/G | G/G | G/A |
| NSCLC-0512 | 1 | 67 | 1 | 1 | 1 | Non-SCC | G/G | G/G | G/G |
| NSCLC-0513 | 2 | 55 | 0 | 0 | 1 | Non-SCC | G/G | G/G | G/G |
| NSCLC-0514 | 2 | 69 | 0 | 0 | 0 | Non-SCC | A/A | G/A | G/A |
| NSCLC-0515 | 2 | 48 | 0 | 0 | 1 | Non-SCC | G/G | G/G | G/G |
| NSCLC-0516 | 2 | 81 | 0 | 0 | 0 | Non-SCC | G/G | G/G | G/G |
| NSCLC-0517 | 2 | 43 | 0 | 0 | 0 | Non-SCC | G/G | G/G | G/G |
| NSCLC-0518 | 1 | 57 | 1 | 0 | 1 | Non-SCC | G/G | G/G | G/G |
| NSCLC-0519 | 2 | 46 | 0 | 0 | 0 | Non-SCC | G/A | G/A | G/A |
| NSCLC-0520 | 2 | 61 | 0 | 0 | 0 | Non-SCC | G/A | G/A | G/A |
| NSCLC-0521 | 2 | 36 | 0 | 0 | 0 | Non-SCC | A/A | G/A | G/A |
| NSCLC-0522 | 2 | 70 | 0 | 0 | 0 | Non-SCC | G/A | G/A | G/A |
| NSCLC-0523 | 1 | 67 | 1 | 1 | 0 | Non-SCC | G/G | G/G | G/G |
| NSCLC-0524 | 1 | 56 | 1 | 1 | 1 | Non-SCC | G/A | G/A | G/G |
| NSCLC-0525 | 2 | 60 | 1 | 1 | 1 | Non-SCC | G/G | G/G | G/G |
| NSCLC-0526 | 2 | 66 | 0 | 0 | 0 | Non-SCC | G/A | G/G | G/A |
| NSCLC-0527 | 1 | 69 | 1 | 1 | 0 | Non-SCC | G/A | G/A | A/A |
| NSCLC-0528 | 1 | 55 | 1 | 1 | 0 | Non-SCC | G/G | G/G | G/G |
| NSCLC-0529 | 2 | 58 | 0 | 0 | 0 | Non-SCC | G/G | G/G | G/G |
| NSCLC-0530 | 2 | 54 | 0 | 0 | 1 | Non-SCC | G/G | G/G | G/G |
| NSCLC-0531 | 1 | 54 | 1 | 1 | 0 | Non-SCC | G/G | G/G | G/G |
| NSCLC-0532 | 1 | 59 | 0 | 0 | 0 | Non-SCC | G/G | G/G | G/G |
| NSCLC-0533 | 2 | 53 | 0 | 0 | 0 | Non-SCC | G/G | G/G | G/A |
| NSCLC-0534 | 1 | 71 | 1 | 1 | 0 | Non-SCC | G/G | G/G | G/A |
| NSCLC-0535 | 1 | 63 | 1 | 1 | 1 | Non-SCC | A/A | A/A | G/A |
| NSCLC-0536 | 2 | 80 | 0 | 0 | 0 | Non-SCC | G/G | G/G | G/G |
| NSCLC-0537 | 1 | 80 | 1 | 1 | 0 | Non-SCC | G/A | G/A | G/A |
| NSCLC-0538 | 1 | 40 | 0 | 0 | 0 | Non-SCC | G/G | G/G | G/G |
| NSCLC-0539 | 2 | 51 | 0 | 0 | 1 | Non-SCC | G/A | G/G | G/A |
| NSCLC-0540 | 2 | 49 | 0 | 0 | 1 | Non-SCC | G/G | G/G | G/G |
| NSCLC-0541 | 2 | 62 | 0 | 0 | 0 | Non-SCC | G/A | G/A | G/A |
| NSCLC-0542 | 1 | 59 | 0 | 1 | 0 | Non-SCC | G/G | G/A | G/G |
| NSCLC-0543 | 2 | 68 | 0 | 0 | 1 | Non-SCC | G/G | G/G | G/G |
| NSCLC-0544 | 2 | 63 | 0 | 0 | 1 | Non-SCC | G/G | G/G | G/G |
| NSCLC-0545 | 1 | 58 | 0 | 0 | 0 | Non-SCC | G/G | G/G | G/G |
| NSCLC-0546 | 2 | 32 | 0 | 0 | 0 | Non-SCC | G/A | G/A | G/A |
| NSCLC-0547 | 1 | 79 | 0 | 0 | 0 | Non-SCC | G/A | G/G | G/G |
| NSCLC-0548 | 2 | 74 | 0 | 0 | 1 | Non-SCC | G/A | G/A | G/A |
| NSCLC-0549 | 1 | 74 | 1 | 0 | 0 | Non-SCC | G/A | G/A | G/A |
| NSCLC-0550 | 1 | 60 | 1 | 0 | 1 | Non-SCC | G/G | G/G | G/G |
| NSCLC-0551 | 2 | 55 | 0 | 0 | 0 | Non-SCC | G/G | G/G | G/G |
| NSCLC-0552 | 1 | 64 | 1 | 1 | 0 | Non-SCC | G/A | G/G | A/A |
| NSCLC-0553 | 1 | 70 | 1 | 1 | 0 | Non-SCC | G/G | G/G | G/G |
| NSCLC-0554 | 1 | 76 | 0 | 0 | 0 | Non-SCC | G/G | G/A | G/G |
| NSCLC-0555 | 1 | 62 | 0 | 1 | 0 | Non-SCC | G/G | G/G | G/G |
| NSCLC-0556 | 1 | 78 | 0 | 0 | 1 | Non-SCC | G/G | G/G | G/G |
| NSCLC-0557 | 1 | 69 | 0 | 0 | 0 | Non-SCC | G/G | G/G | G/G |
| NSCLC-0558 | 2 | 74 | 0 | 0 | 0 | Non-SCC | G/G | G/G | G/G |
| NSCLC-0559 | 1 | 57 | 0 | 0 | 0 | Non-SCC | G/G | G/G | G/G |
| NSCLC-0560 | 1 | 67 | 1 | 0 | 0 | Non-SCC | G/A | G/G | G/G |
| NSCLC-0561 | 2 | 57 | 0 | 0 | 0 | Non-SCC | G/G | G/G | G/G |
| NSCLC-0562 | 2 | 26 | 0 | 0 | 1 | Non-SCC | G/G | G/G | G/G |
| NSCLC-0563 | 1 | 61 | 1 | 1 | 0 | Non-SCC | G/G | G/G | G/G |
| NSCLC-0564 | 2 | 79 | 0 | 0 | 0 | Non-SCC | G/G | G/G | G/G |
| NSCLC-0565 | 1 | 49 | 1 | 1 | 1 | Non-SCC | G/A | G/G | G/G |
| NSCLC-0566 | 1 | 61 | 0 | 1 | 0 | Non-SCC | G/G | G/G | G/G |
| NSCLC-0567 | 1 | 58 | 1 | 1 | 0 | Non-SCC | G/G | G/G | G/G |
| NSCLC-0568 | 1 | 66 | 1 | 0 | 0 | Non-SCC | G/A | G/A | G/A |
| NSCLC-0569 | 2 | 46 | 0 | 0 | 0 | Non-SCC | G/A | G/A | G/G |
| NSCLC-0570 | 2 | 75 | 0 | 0 | 0 | Non-SCC | G/G | G/G | G/G |
| NSCLC-0571 | 1 | 61 | 0 | 0 | 0 | Non-SCC | G/G | G/G | G/G |
| NSCLC-0572 | 1 | 64 | 0 | 0 | 0 | Non-SCC | G/A | G/G | G/G |
| NSCLC-0573 | 2 | 46 | 0 | 0 | 0 | Non-SCC | G/A | G/A | G/G |
| NSCLC-0574 | 1 | 82 | 0 | 0 | 1 | Non-SCC | G/A | G/A | G/A |
| NSCLC-0575 | 1 | 71 | 1 | 0 | 0 | Non-SCC | G/G | G/G | G/A |
| NSCLC-0576 | 1 | 61 | 0 | 0 | 1 | Non-SCC | G/G | G/G | G/A |
| NSCLC-0577 | 2 | 71 | 0 | 0 | 0 | Non-SCC | G/G | G/A | G/A |
| NSCLC-0578 | 2 | 67 | 0 | 1 | 1 | Non-SCC | G/G | G/G | G/G |
| NSCLC-0579 | 1 | 52 | 1 | 1 | 0 | Non-SCC | G/G | G/G | G/G |
| NSCLC-0580 | 2 | 64 | 0 | 0 | 0 | Non-SCC | G/G | G/G | G/G |
| NSCLC-0581 | 2 | 46 | 0 | 0 | 0 | Non-SCC | G/A | G/A | G/A |
| NSCLC-0582 | 2 | 61 | 0 | 0 | 1 | Non-SCC | G/G | G/G | G/G |
| NSCLC-0583 | 2 | 60 | 0 | 0 | 0 | Non-SCC | G/G | G/G | G/G |
| NSCLC-0584 | 1 | 53 | 1 | 1 | 1 | Non-SCC | G/G | G/G | G/A |
| NSCLC-0585 | 1 | 75 | 0 | 0 | 0 | Non-SCC | G/A | G/A | G/A |
| NSCLC-0586 | 1 | 79 | 0 | 0 | 1 | Non-SCC | G/G | G/G | G/G |
| NSCLC-0587 | 1 | 70 | 1 | 1 | 0 | Non-SCC | G/G | G/G | G/G |
| NSCLC-0588 | 1 | 61 | 1 | 0 | 0 | Non-SCC | G/G | G/G | G/A |
| NSCLC-0589 | 1 | 68 | 1 | 1 | 0 | Non-SCC | G/G | G/G | G/A |
| NSCLC-0590 | 2 | 59 | 0 | 0 | 0 | Non-SCC | A/A | A/A | G/G |
| NSCLC-0591 | 2 | 55 | 0 | 0 | 1 | Non-SCC | G/G | G/G | G/G |
| NSCLC-0592 | 2 | 59 | 0 | 0 | 1 | Non-SCC | G/G | G/G | G/G |
| NSCLC-0593 | 1 | 70 | 0 | 0 | 1 | Non-SCC | G/G | G/G | G/G |
| NSCLC-0594 | 1 | 52 | 1 | 0 | 0 | Non-SCC | G/G | G/G | G/A |
| NSCLC-0595 | 1 | 65 | 1 | 0 | 0 | Non-SCC | G/G | G/G | G/G |
| NSCLC-0596 | 2 | 58 | 0 | 0 | 0 | Non-SCC | G/A | G/A | G/G |
| NSCLC-0597 | 1 | 74 | 1 | 1 | 0 | Non-SCC | G/G | G/G | G/G |
| NSCLC-0598 | 2 | 66 | 0 | 0 | 0 | Non-SCC | G/G | G/G | G/A |
| NSCLC-0599 | 2 | 56 | 0 | 0 | 0 | Non-SCC | G/G | G/G | G/G |
| NSCLC-0600 | 2 | 67 | 0 | 0 | 0 | Non-SCC | G/G | G/G | G/G |
| NSCLC-0601 | 1 | 54 | 1 | 0 | 0 | Non-SCC | G/G | G/G | G/G |
| NSCLC-0602 | 2 | 59 | 0 | 0 | 1 | Non-SCC | G/G | G/G | G/G |
| NSCLC-0603 | 1 | 63 | 0 | 0 | 0 | Non-SCC | G/A | G/A | G/G |
| NSCLC-0604 | 2 | 65 | 1 | 0 | 1 | Non-SCC | G/G | G/G | G/G |
| NSCLC-0605 | 1 | 67 | 0 | 0 | 1 | Non-SCC | G/A | G/A | G/G |
| NSCLC-0606 | 1 | 63 | 0 | 0 | 1 | Non-SCC | G/G | G/G | G/G |
| NSCLC-0607 | 1 | 62 | 1 | 0 | 1 | Non-SCC | G/G | G/G | G/G |
| NSCLC-0608 | 1 | 81 | 0 | 0 | 1 | Non-SCC | G/G | G/G | G/A |
| NSCLC-0609 | 2 | 51 | 0 | 0 | 1 | Non-SCC | G/A | G/G | G/G |
| NSCLC-0610 | 2 | 52 | 0 | 0 | 0 | Non-SCC | G/A | G/A | G/A |
| NSCLC-0611 | 2 | 48 | 0 | 0 | 1 | Non-SCC | G/G | G/G | G/G |
| NSCLC-0612 | 1 | 67 | 1 | 1 | 0 | Non-SCC | G/G | G/G | G/G |
| NSCLC-0613 | 1 | 62 | 1 | 1 | 0 | Non-SCC | G/A | G/A | G/A |
| NSCLC-0614 | 1 | 66 | 1 | 1 | 0 | Non-SCC | G/A | G/A | G/A |
| NSCLC-0615 | 2 | 71 | 0 | 0 | 0 | Non-SCC | G/G | G/G | G/G |
| NSCLC-0616 | 2 | 61 | 0 | 0 | 1 | Non-SCC | G/G | G/G | G/G |
| NSCLC-0617 | 1 | 43 | 0 | 1 | 0 | Non-SCC | G/A | G/A | G/A |
| NSCLC-0618 | 1 | 66 | 1 | 1 | 0 | Non-SCC | G/G | G/G | G/G |
| NSCLC-0619 | 1 | 71 | 1 | 0 | 1 | Non-SCC | G/G | G/G | G/G |
| NSCLC-0620 | 2 | 72 | 0 | 0 | 0 | Non-SCC | G/G | G/G | G/G |
| NSCLC-0621 | 1 | 69 | 1 | 0 | 0 | Non-SCC | G/G | G/G | G/G |
| NSCLC-0622 | 1 | 85 | 0 | 1 | 1 | Non-SCC | G/G | G/G | G/G |
| NSCLC-0623 | 2 | 65 | 0 | 0 | 1 | Non-SCC | A/A | G/A | G/G |
| NSCLC-0624 | 2 | 64 | 0 | 0 | 1 | Non-SCC | G/A | G/G | G/A |
| NSCLC-0625 | 1 | 35 | 1 | 0 | 0 | Non-SCC | G/A | G/A | G/A |
| NSCLC-0626 | 2 | 48 | 0 | 0 | 1 | Non-SCC | ? | ? | ? |
| NSCLC-0627 | 2 | 55 | 0 | 0 | 1 | Non-SCC | G/G | G/G | G/G |
| NSCLC-0628 | 2 | 40 | 0 | 0 | 0 | Non-SCC | ? | ? | ? |
| NSCLC-0629 | 2 | 46 | 0 | 0 | 0 | Non-SCC | G/G | G/G | G/G |
| NSCLC-0630 | 1 | 68 | 0 | 1 | 0 | Non-SCC | G/G | G/G | G/G |
| NSCLC-0631 | 2 | 71 | 0 | 0 | 1 | Non-SCC | G/G | G/G | G/G |
| NSCLC-0632 | 2 | 37 | 0 | 0 | 1 | Non-SCC | G/G | G/G | G/G |
| NSCLC-0633 | 2 | 64 | 0 | 0 | 0 | Non-SCC | G/G | G/G | G/G |
| NSCLC-0634 | 1 | 44 | 1 | 1 | 1 | Non-SCC | G/G | G/G | G/G |
| NSCLC-0635 | 1 | 46 | 0 | 0 | 1 | Non-SCC | G/G | G/G | G/G |
| NSCLC-0636 | 1 | 64 | 1 | 1 | 0 | Non-SCC | G/A | G/A | G/G |
| NSCLC-0637 | 1 | 54 | 1 | 0 | 1 | Non-SCC | G/G | G/G | G/G |
| NSCLC-0638 | 2 | 63 | 0 | 0 | 0 | Non-SCC | G/A | G/G | G/A |
| NSCLC-0639 | 2 | 71 | 0 | 0 | 0 | Non-SCC | G/G | G/G | G/G |
| NSCLC-0640 | 2 | 53 | 0 | 0 | 0 | Non-SCC | G/G | G/G | G/A |
| NSCLC-0641 | 2 | 49 | 0 | 0 | 0 | Non-SCC | G/G | G/G | G/A |
| NSCLC-0642 | 2 | 35 | 0 | 0 | 0 | Non-SCC | G/G | G/G | G/A |
| NSCLC-0643 | 2 | 48 | 0 | 0 | 0 | Non-SCC | G/G | G/G | G/A |
| NSCLC-0644 | 1 | 68 | 0 | 1 | 0 | Non-SCC | G/G | G/G | G/G |
| NSCLC-0645 | 2 | 80 | 0 | 0 | 1 | Non-SCC | G/G | G/G | G/G |
| NSCLC-0646 | 2 | 53 | 0 | 0 | 0 | Non-SCC | G/G | G/G | G/G |
| NSCLC-0647 | 1 | 55 | 0 | 0 | 1 | Non-SCC | G/G | G/G | G/G |
| NSCLC-0648 | 1 | 33 | 0 | 0 | 0 | Non-SCC | G/G | G/G | G/G |
| NSCLC-0649 | 2 | 64 | 0 | 0 | 1 | Non-SCC | G/A | G/A | G/G |
| NSCLC-0650 | 2 | 63 | 0 | 0 | 1 | Non-SCC | G/G | G/G | G/G |
| NSCLC-0651 | 1 | 81 | 0 | 0 | 0 | Non-SCC | G/G | G/G | G/G |
| NSCLC-0652 | 2 | 68 | 0 | 0 | 0 | Non-SCC | G/G | G/G | G/G |
| NSCLC-0653 | 1 | 63 | 0 | 0 | 1 | Non-SCC | G/G | G/G | G/G |
| NSCLC-0654 | 2 | 62 | 0 | 0 | 0 | Non-SCC | G/G | G/G | G/G |
| NSCLC-0655 | 2 | 53 | 0 | 0 | 0 | Non-SCC | G/G | G/G | G/G |
| NSCLC-0656 | 2 | 56 | 0 | 0 | 0 | Non-SCC | G/G | G/G | G/G |
| NSCLC-0657 | 1 | 61 | 1 | 1 | 0 | Non-SCC | G/G | G/G | G/G |
| NSCLC-0658 | 1 | 55 | 0 | 0 | 0 | Non-SCC | G/G | G/G | G/A |
| NSCLC-0659 | 1 | 43 | 0 | 0 | 0 | Non-SCC | G/A | A/A | G/G |
| NSCLC-0660 | 1 | 67 | 1 | 1 | 1 | Non-SCC | G/G | G/G | G/A |
| NSCLC-0661 | 2 | 49 | 0 | 0 | 0 | Non-SCC | A/A | G/A | G/A |
| NSCLC-0662 | 2 | 63 | 0 | 0 | 0 | Non-SCC | G/G | G/G | G/G |
| NSCLC-0663 | 1 | 64 | 1 | 1 | 0 | Non-SCC | G/G | G/G | G/G |
| NSCLC-0664 | 2 | 50 | 0 | 0 | 0 | Non-SCC | G/G | G/G | G/G |
| NSCLC-0665 | 1 | 72 | 0 | 0 | 1 | Non-SCC | G/A | G/G | G/G |
| NSCLC-0666 | 2 | 66 | 0 | 0 | 1 | Non-SCC | G/G | G/G | G/G |
| NSCLC-0667 | 2 | 57 | 0 | 0 | 0 | Non-SCC | G/G | G/G | G/A |
| NSCLC-0668 | 1 | 43 | 1 | 0 | 0 | Non-SCC | G/G | G/G | G/G |
| NSCLC-0669 | 2 | 62 | 0 | 0 | 1 | Non-SCC | G/G | G/A | G/G |
| NSCLC-0670 | 1 | 52 | 0 | 0 | 0 | Non-SCC | G/A | G/G | G/G |
| NSCLC-0671 | 2 | 54 | 0 | 0 | 0 | Non-SCC | G/G | G/G | G/A |
| NSCLC-0672 | 1 | 42 | 0 | 0 | 1 | Non-SCC | G/G | G/G | G/G |
| NSCLC-0673 | 1 | 62 | 1 | 1 | 0 | Non-SCC | G/G | G/G | G/G |
| NSCLC-0674 | 1 | 54 | 0 | 0 | 0 | Non-SCC | G/G | G/G | G/G |
| NSCLC-0675 | 1 | 61 | 0 | 1 | 0 | Non-SCC | G/G | G/G | G/G |
| NSCLC-0676 | 1 | 75 | 1 | 1 | 0 | Non-SCC | G/A | G/A | G/A |
| NSCLC-0677 | 2 | 48 | 0 | 0 | 0 | Non-SCC | G/G | G/G | G/G |
| NSCLC-0678 | 1 | 66 | 1 | 1 | 1 | Non-SCC | G/G | G/G | G/A |
| NSCLC-0679 | 2 | 52 | 1 | 1 | 0 | Non-SCC | G/G | G/G | G/G |
| NSCLC-0680 | 1 | 50 | 0 | 0 | 0 | Non-SCC | G/G | G/G | G/G |
| NSCLC-0681 | 2 | 55 | 0 | 0 | 0 | Non-SCC | G/G | G/G | G/G |
| NSCLC-0682 | 1 | 61 | 1 | 1 | 0 | Non-SCC | G/G | G/G | G/G |
| NSCLC-0683 | 2 | 53 | 0 | 0 | 0 | Non-SCC | G/G | G/A | G/G |
| NSCLC-0684 | 2 | 53 | 0 | 0 | 1 | Non-SCC | G/G | G/G | G/G |
| NSCLC-0685 | 1 | 66 | 0 | 0 | 0 | Non-SCC | G/G | G/G | G/G |
| NSCLC-0686 | 1 | 68 | 0 | 0 | 0 | Non-SCC | G/G | G/G | G/A |
| NSCLC-0687 | 2 | 62 | 0 | 0 | 0 | Non-SCC | G/A | G/A | G/G |
| NSCLC-0688 | 2 | 48 | 0 | 0 | 0 | Non-SCC | G/G | G/G | G/G |
| NSCLC-0689 | 1 | 57 | 1 | 1 | 0 | Non-SCC | G/G | G/G | G/G |
| NSCLC-0690 | 2 | 44 | 0 | 0 | 1 | Non-SCC | G/A | G/A | G/A |
| NSCLC-0691 | 2 | 64 | 0 | 0 | 1 | Non-SCC | G/A | G/G | G/G |
| NSCLC-0692 | 1 | 55 | 1 | 1 | 1 | Non-SCC | G/A | G/A | G/A |
| NSCLC-0693 | 2 | 63 | 0 | 0 | 0 | Non-SCC | G/G | G/G | G/G |
| NSCLC-0694 | 2 | 66 | 0 | 0 | 1 | Non-SCC | G/G | G/G | G/G |
| NSCLC-0695 | 1 | 33 | 1 | 1 | 1 | Non-SCC | G/G | G/G | G/G |
| NSCLC-0696 | 2 | 53 | 0 | 0 | 1 | Non-SCC | G/A | G/G | G/G |
| NSCLC-0697 | 2 | 76 | 0 | 0 | 0 | Non-SCC | G/G | G/G | G/G |
| NSCLC-0698 | 2 | 54 | 0 | 0 | 0 | Non-SCC | G/A | G/G | G/A |
| NSCLC-0699 | 2 | 53 | 0 | 0 | 1 | Non-SCC | G/G | G/G | G/G |
| NSCLC-0700 | 2 | 57 | 0 | 0 | 0 | Non-SCC | G/G | G/G | G/G |
| NSCLC-0701 | 1 | 62 | 0 | 0 | 0 | Non-SCC | G/G | G/G | G/G |
| NSCLC-0702 | 1 | 78 | 1 | 1 | 0 | Non-SCC | G/G | G/G | G/G |
| NSCLC-0703 | 1 | 51 | 0 | 0 | 1 | Non-SCC | G/G | G/G | G/A |
| NSCLC-0704 | 2 | 48 | 0 | 0 | 0 | Non-SCC | G/A | G/A | G/G |
| NSCLC-0705 | 2 | 78 | 0 | 0 | 1 | Non-SCC | G/A | G/G | G/A |
| NSCLC-0706 | 2 | 63 | 0 | 0 | 1 | Non-SCC | G/A | G/A | G/A |
| NSCLC-0707 | 2 | 75 | 0 | 0 | 1 | Non-SCC | G/G | G/G | G/G |
| NSCLC-0708 | 2 | 46 | 0 | 0 | 0 | Non-SCC | G/G | G/G | G/G |
| NSCLC-0709 | 1 | 47 | 1 | 0 | 0 | Non-SCC | G/G | G/G | G/G |
| NSCLC-0710 | 2 | 65 | 0 | 0 | 0 | Non-SCC | A/A | G/G | G/G |
| NSCLC-0711 | 2 | 81 | 0 | 0 | 1 | Non-SCC | G/A | G/G | A/A |
| NSCLC-0712 | 2 | 54 | 0 | 0 | 0 | Non-SCC | G/G | G/G | G/G |
| NSCLC-0713 | 1 | 54 | 0 | 1 | 0 | Non-SCC | G/G | G/G | G/G |
| NSCLC-0714 | 1 | 62 | 1 | 0 | 0 | Non-SCC | G/A | G/A | G/G |
| NSCLC-0715 | 2 | 39 | 0 | 0 | 0 | Non-SCC | G/G | G/G | G/A |
| NSCLC-0716 | 1 | 54 | 1 | 1 | 1 | Non-SCC | G/G | G/G | G/G |
| NSCLC-0717 | 1 | 54 | 1 | 1 | 0 | Non-SCC | G/A | G/A | G/A |
| NSCLC-0718 | 2 | 48 | 0 | 0 | 1 | Non-SCC | G/G | G/G | G/G |
| NSCLC-0719 | 1 | 59 | 1 | 1 | 0 | Non-SCC | G/G | G/G | G/G |
| NSCLC-0720 | 1 | 77 | 0 | 0 | 0 | Non-SCC | G/G | G/G | G/A |
| NSCLC-0721 | 2 | 51 | 0 | 0 | 0 | Non-SCC | G/A | G/A | G/A |
| NSCLC-0722 | 2 | 45 | 0 | 0 | 0 | Non-SCC | G/G | G/G | G/G |
| NSCLC-0723 | 1 | 53 | 1 | 1 | 0 | Non-SCC | G/A | G/A | G/A |
| NSCLC-0724 | 2 | 57 | 0 | 0 | 0 | Non-SCC | G/G | G/G | G/G |
| NSCLC-0725 | 1 | 37 | 1 | 0 | 0 | Non-SCC | G/G | G/G | G/A |
| NSCLC-0726 | 1 | 70 | 0 | 0 | 0 | Non-SCC | G/G | G/G | G/G |
| NSCLC-0727 | 1 | 54 | 0 | 1 | 0 | Non-SCC | G/G | G/G | G/G |
| NSCLC-0728 | 2 | 64 | 0 | 0 | 0 | Non-SCC | A/A | A/A | G/G |
| NSCLC-0729 | 1 | 53 | 1 | 1 | 1 | Non-SCC | G/G | G/G | G/A |
| NSCLC-0730 | 2 | 55 | 0 | 0 | 0 | Non-SCC | ? | ? | ? |
| NSCLC-0731 | 2 | 59 | 0 | 0 | 1 | Non-SCC | G/G | G/G | G/G |
| NSCLC-0732 | 2 | 71 | 0 | 0 | 0 | Non-SCC | G/A | G/G | G/G |
| NSCLC-0733 | 1 | 62 | 0 | 0 | 0 | Non-SCC | G/A | G/A | G/G |
| NSCLC-0734 | 1 | 44 | 0 | 0 | 1 | Non-SCC | G/G | G/G | G/G |
| NSCLC-0735 | 1 | 57 | 0 | 0 | 1 | Non-SCC | ? | ? | ? |
| NSCLC-0736 | 1 | 61 | 0 | 0 | 1 | Non-SCC | G/A | G/G | G/G |
| NSCLC-0737 | 1 | 61 | 0 | 0 | 0 | Non-SCC | G/G | G/G | G/A |
| NSCLC-0738 | 2 | 54 | 0 | 0 | 0 | Non-SCC | G/A | G/G | G/A |
| NSCLC-0739 | 1 | 71 | 1 | 1 | 0 | Non-SCC | G/G | G/G | G/G |
| NSCLC-0740 | 2 | 62 | 0 | 0 | 0 | Non-SCC | G/G | G/G | G/G |
| NSCLC-0741 | 1 | 30 | 0 | 0 | 1 | Non-SCC | A/A | A/A | G/G |
| NSCLC-0742 | 2 | 61 | 0 | 0 | 0 | Non-SCC | G/G | G/G | G/G |
| NSCLC-0743 | 2 | 51 | 0 | 0 | 0 | Non-SCC | G/G | G/G | G/G |
| NSCLC-0744 | 2 | 65 | 0 | 0 | 0 | Non-SCC | G/G | G/A | G/G |
| NSCLC-0745 | 2 | 72 | 0 | 0 | 0 | Non-SCC | G/G | G/G | G/G |
| NSCLC-0746 | 2 | 71 | 0 | 0 | 1 | Non-SCC | G/A | G/A | G/G |
| NSCLC-0747 | 2 | 66 | 0 | 0 | 0 | Non-SCC | G/G | G/G | G/G |
| NSCLC-0748 | 1 | 60 | 1 | 1 | 1 | Non-SCC | G/G | G/G | G/G |
| NSCLC-0749 | 1 | 68 | 1 | 1 | 0 | Non-SCC | G/G | G/G | G/G |
| NSCLC-0750 | 1 | 65 | 1 | 0 | 0 | Non-SCC | G/A | G/G | G/G |
| NSCLC-0751 | 1 | 51 | 0 | 1 | 1 | Non-SCC | G/G | G/G | G/G |
| NSCLC-0752 | 2 | 45 | 0 | 0 | 0 | Non-SCC | G/G | G/G | G/G |
| NSCLC-0753 | 1 | 66 | 0 | 1 | 0 | Non-SCC | G/G | G/G | G/G |
| NSCLC-0754 | 2 | 49 | 0 | 0 | 0 | Non-SCC | G/G | G/G | G/G |
| NSCLC-0755 | 2 | 59 | 0 | 0 | 0 | Non-SCC | G/G | G/G | G/G |
| NSCLC-0756 | 2 | 59 | 0 | 0 | 0 | Non-SCC | G/G | G/G | G/G |
| NSCLC-0757 | 1 | 71 | 1 | 0 | 1 | Non-SCC | G/G | G/G | G/G |
| NSCLC-0758 | 2 | 79 | 0 | 0 | 0 | Non-SCC | G/G | G/G | G/G |
| NSCLC-0759 | 1 | 80 | 0 | 0 | 0 | Non-SCC | G/G | G/G | G/G |
| NSCLC-0760 | 1 | 64 | 1 | 1 | 1 | Non-SCC | G/G | G/G | G/G |
| NSCLC-0761 | 1 | 67 | 1 | 0 | 0 | Non-SCC | G/G | G/G | G/G |
| NSCLC-0762 | 2 | 69 | 0 | 0 | 1 | Non-SCC | G/A | G/A | G/A |
| NSCLC-0763 | 2 | 60 | 0 | 0 | 0 | Non-SCC | G/G | G/G | G/G |
| NSCLC-0764 | 2 | 46 | 0 | 0 | 0 | Non-SCC | G/G | G/G | G/G |
| NSCLC-0765 | 2 | 59 | 0 | 0 | 0 | Non-SCC | G/G | G/A | G/G |
| NSCLC-0766 | 1 | 59 | 1 | 0 | 0 | Non-SCC | G/A | G/A | A/A |
| NSCLC-0767 | 2 | 63 | 0 | 0 | 0 | Non-SCC | G/G | G/G | G/G |
| NSCLC-0768 | 1 | 42 | 0 | 0 | 0 | Non-SCC | G/G | G/G | G/G |
| NSCLC-0769 | 2 | 52 | 0 | 0 | 1 | Non-SCC | G/G | G/G | G/A |
| NSCLC-0770 | 1 | 47 | 0 | 0 | 1 | Non-SCC | G/A | G/G | G/A |
| NSCLC-0771 | 2 | 70 | 0 | 0 | 1 | Non-SCC | G/G | G/G | G/G |
| NSCLC-0772 | 1 | 62 | 1 | 1 | 1 | Non-SCC | G/G | G/G | G/G |
| NSCLC-0773 | 2 | 45 | 0 | 0 | 0 | Non-SCC | G/G | G/G | G/G |
| NSCLC-0774 | 2 | 52 | 0 | 0 | 0 | Non-SCC | G/A | G/A | G/A |
| NSCLC-0775 | 2 | 72 | 0 | 0 | 1 | Non-SCC | G/G | G/G | G/G |
| NSCLC-0776 | 2 | 57 | 0 | 0 | 0 | Non-SCC | G/G | G/G | G/G |
| NSCLC-0777 | 2 | 58 | 0 | 0 | 1 | Non-SCC | G/A | G/A | G/A |
| NSCLC-0778 | 1 | 58 | 1 | 1 | 0 | Non-SCC | G/G | G/G | G/A |
| NSCLC-0779 | 1 | 59 | 1 | 0 | 1 | Non-SCC | G/G | G/G | G/G |
| NSCLC-0780 | 2 | 61 | 0 | 0 | 1 | Non-SCC | G/A | G/A | G/A |
| NSCLC-0781 | 2 | 67 | 0 | 0 | 0 | Non-SCC | G/A | G/G | G/G |
| NSCLC-0782 | 2 | 35 | 0 | 0 | 0 | Non-SCC | G/G | G/G | G/G |
| NSCLC-0783 | 2 | 61 | 0 | 0 | 1 | Non-SCC | G/A | G/G | G/G |
| NSCLC-0784 | 2 | 48 | 0 | 0 | 0 | Non-SCC | G/G | G/G | G/G |
| NSCLC-0785 | 1 | 60 | 1 | 1 | 1 | Non-SCC | G/G | G/A | G/G |
| NSCLC-0786 | 2 | 53 | 0 | 0 | 1 | Non-SCC | G/A | G/A | G/G |
| NSCLC-0787 | 2 | 65 | 0 | 0 | 0 | Non-SCC | G/G | G/G | G/G |
| NSCLC-0788 | 2 | 45 | 0 | 0 | 0 | Non-SCC | G/A | G/G | G/G |
| NSCLC-0789 | 2 | 43 | 0 | 0 | 0 | Non-SCC | G/G | G/G | G/G |
| NSCLC-0790 | 2 | 66 | 0 | 0 | 0 | Non-SCC | G/G | G/G | G/G |
| NSCLC-0791 | 1 | 55 | 1 | 1 | 1 | Non-SCC | G/A | G/A | G/G |
| NSCLC-0792 | 2 | 45 | 0 | 0 | 0 | Non-SCC | G/G | G/G | G/G |
| NSCLC-0793 | 1 | 27 | 0 | 0 | 0 | Non-SCC | G/G | G/G | G/G |
| NSCLC-0794 | 2 | 32 | 0 | 0 | 0 | Non-SCC | G/G | G/G | G/G |
| NSCLC-0795 | 1 | 68 | 1 | 1 | 1 | Non-SCC | G/G | G/G | G/G |
| NSCLC-0796 | 2 | 61 | 0 | 0 | 0 | Non-SCC | G/A | G/A | G/A |
| NSCLC-0797 | 2 | 42 | 0 | 0 | 1 | Non-SCC | G/G | G/A | G/A |
| NSCLC-0798 | 1 | 67 | 1 | 1 | 1 | Non-SCC | G/G | G/G | G/G |
| NSCLC-0799 | 2 | 59 | 0 | 0 | 0 | Non-SCC | G/G | G/G | G/G |
| NSCLC-0800 | 2 | 57 | 0 | 0 | 0 | Non-SCC | G/G | G/G | G/G |
| NSCLC-0801 | 1 | 51 | 0 | 1 | 1 | Non-SCC | G/G | G/G | G/G |
| NSCLC-0802 | 2 | 40 | 0 | 0 | 0 | Non-SCC | G/G | G/G | G/G |
| NSCLC-0803 | 1 | 71 | 0 | 0 | 0 | Non-SCC | G/G | G/G | G/G |
| NSCLC-0804 | 2 | 50 | 0 | 0 | 0 | Non-SCC | G/G | G/G | G/G |
| NSCLC-0805 | 2 | 55 | 0 | 0 | 0 | Non-SCC | G/A | G/G | G/G |
| NSCLC-0806 | 2 | 40 | 0 | 0 | 0 | Non-SCC | G/G | G/G | G/G |
| NSCLC-0807 | 1 | 66 | 0 | 1 | 1 | Non-SCC | G/G | G/G | G/A |
| NSCLC-0808 | 2 | 37 | 0 | 0 | 0 | Non-SCC | G/G | G/G | G/G |
| NSCLC-0809 | 2 | 61 | 0 | 0 | 0 | Non-SCC | G/G | G/G | G/G |
| NSCLC-0810 | 2 | 44 | 0 | 0 | 0 | Non-SCC | G/G | G/G | G/A |
| NSCLC-0811 | 1 | 59 | 0 | 0 | 0 | Non-SCC | G/G | G/G | G/G |
| NSCLC-0812 | 1 | 75 | 1 | 1 | 0 | Non-SCC | ? | ? | ? |
| NSCLC-0813 | 1 | 53 | 1 | 1 | 0 | Non-SCC | G/G | G/G | G/G |
| NSCLC-0814 | 2 | 64 | 0 | 0 | 0 | Non-SCC | G/A | G/A | G/G |
| NSCLC-0815 | 2 | 54 | 0 | 0 | 0 | Non-SCC | G/A | G/G | A/A |
| NSCLC-0816 | 2 | 53 | 0 | 0 | 0 | Non-SCC | G/G | G/G | G/G |
| NSCLC-0817 | 2 | 32 | 0 | 0 | 0 | Non-SCC | G/A | G/A | G/G |
| NSCLC-0818 | 2 | 53 | 0 | 0 | 0 | Non-SCC | G/A | G/G | G/A |
| NSCLC-0819 | 1 | 62 | 1 | 1 | 0 | Non-SCC | G/G | G/G | G/G |
| NSCLC-0820 | 1 | 70 | 0 | 0 | 0 | Non-SCC | G/G | G/G | G/G |
| NSCLC-0821 | 2 | 48 | 0 | 0 | 1 | Non-SCC | G/G | G/G | G/A |
| NSCLC-0822 | 2 | 59 | 0 | 0 | 0 | Non-SCC | G/G | G/G | G/G |
| NSCLC-0823 | 2 | 54 | 0 | 0 | 0 | Non-SCC | G/A | G/A | A/A |
| NSCLC-0824 | 1 | 59 | 1 | 1 | 0 | Non-SCC | G/G | G/A | G/G |
| NSCLC-0825 | 2 | 62 | 0 | 0 | 0 | Non-SCC | G/A | G/A | G/A |
| NSCLC-0826 | 2 | 65 | 0 | 0 | 0 | Non-SCC | G/G | G/G | G/G |
| NSCLC-0827 | 2 | 40 | 0 | 0 | 0 | Non-SCC | G/A | G/G | G/G |
| NSCLC-0828 | 2 | 45 | 0 | 0 | 0 | Non-SCC | G/A | G/G | G/A |
| NSCLC-0829 | 1 | 64 | 1 | 0 | 0 | Non-SCC | G/G | G/G | G/A |
| NSCLC-0830 | 1 | 70 | 1 | 0 | 0 | Non-SCC | G/G | G/G | G/G |
| NSCLC-0831 | 1 | 52 | 1 | 0 | 0 | Non-SCC | G/G | G/G | G/G |
| NSCLC-0832 | 1 | 48 | 1 | 0 | 1 | Non-SCC | G/G | G/G | G/G |
| NSCLC-0833 | 1 | 64 | 1 | 0 | 1 | Non-SCC | G/G | G/G | G/A |
| NSCLC-0834 | 1 | 43 | 0 | 1 | 0 | Non-SCC | G/A | G/A | G/A |
| NSCLC-0835 | 1 | 61 | 1 | 0 | 0 | Non-SCC | G/G | G/G | G/A |
| NSCLC-0836 | 2 | 54 | 0 | 0 | 0 | Non-SCC | G/G | G/G | G/A |
| NSCLC-0837 | 1 | 69 | 1 | 1 | 1 | Non-SCC | G/A | G/A | A/A |
| NSCLC-0838 | 2 | 60 | 0 | 0 | 0 | Non-SCC | G/G | G/G | G/G |
| NSCLC-0839 | 2 | 54 | 0 | 0 | 0 | Non-SCC | G/G | G/G | G/G |
| NSCLC-0840 | 2 | 65 | 0 | 0 | 1 | Non-SCC | G/G | G/G | G/G |
| NSCLC-0841 | 2 | 63 | 1 | 1 | 0 | Non-SCC | G/G | G/A | G/G |
| NSCLC-0842 | 2 | 50 | 0 | 0 | 0 | Non-SCC | G/A | G/A | G/A |
| NSCLC-0843 | 1 | 61 | 0 | 0 | 0 | Non-SCC | G/G | G/G | G/G |
| NSCLC-0844 | 2 | 49 | 0 | 0 | 0 | Non-SCC | G/A | G/G | G/G |
| NSCLC-0845 | 2 | 52 | 0 | 0 | 0 | Non-SCC | G/G | G/G | G/G |
| NSCLC-0846 | 2 | 44 | 0 | 0 | 0 | Non-SCC | G/G | G/G | G/G |
| NSCLC-0847 | 2 | 60 | 0 | 0 | 0 | Non-SCC | G/G | G/A | G/G |
| NSCLC-0848 | 2 | 71 | 0 | 0 | 1 | Non-SCC | G/A | G/A | G/A |
| NSCLC-0849 | 1 | 54 | 0 | 0 | 0 | Non-SCC | G/G | G/G | G/G |
| NSCLC-0850 | 2 | 54 | 0 | 0 | 0 | Non-SCC | G/G | G/G | G/G |
| NSCLC-0851 | 1 | 55 | 1 | 1 | 1 | Non-SCC | G/G | G/G | G/G |
| NSCLC-0852 | 1 | 72 | 1 | 1 | 0 | Non-SCC | G/A | G/G | G/A |
| NSCLC-0853 | 1 | 45 | 0 | 0 | 0 | Non-SCC | G/G | G/G | G/G |
| NSCLC-0854 | 1 | 60 | 0 | 0 | 1 | Non-SCC | G/G | G/G | G/G |
| NSCLC-0855 | 2 | 28 | 0 | 0 | 0 | Non-SCC | G/G | G/G | G/A |
| NSCLC-0856 | 2 | 73 | 0 | 0 | 0 | Non-SCC | G/A | G/A | G/G |
| NSCLC-0857 | 2 | 65 | 0 | 0 | 0 | Non-SCC | G/G | G/G | G/G |
| NSCLC-0858 | 1 | 58 | 1 | 1 | 0 | Non-SCC | G/G | G/G | G/G |
| NSCLC-0859 | 2 | 70 | 0 | 0 | 0 | Non-SCC | G/G | G/G | G/G |
| NSCLC-0860 | 1 | 51 | 1 | 1 | 0 | Non-SCC | G/G | G/G | G/G |
| NSCLC-0861 | 2 | 62 | 0 | 0 | 0 | Non-SCC | G/G | G/G | G/G |
| NSCLC-0862 | 2 | 44 | 0 | 0 | 0 | Non-SCC | G/A | G/A | G/G |
| NSCLC-0863 | 1 | 58 | 1 | 0 | 1 | Non-SCC | G/G | G/G | G/G |
| NSCLC-0864 | 1 | 60 | 0 | 1 | 0 | Non-SCC | G/G | G/G | G/G |
| NSCLC-0865 | 2 | 66 | 0 | 0 | 1 | Non-SCC | G/G | G/G | G/G |
| NSCLC-0866 | 1 | 63 | 0 | 0 | 0 | Non-SCC | G/A | G/G | G/A |
| NSCLC-0867 | 2 | 65 | 0 | 0 | 0 | Non-SCC | G/A | G/A | A/A |
| NSCLC-0868 | 2 | 72 | 0 | 0 | 0 | Non-SCC | G/A | G/G | G/A |
| NSCLC-0869 | 1 | 60 | 0 | 0 | 0 | Non-SCC | G/A | G/A | G/G |
| NSCLC-0870 | 2 | 47 | 0 | 0 | 1 | Non-SCC | G/G | G/G | G/A |
| NSCLC-0871 | 1 | 73 | 0 | 1 | 0 | Non-SCC | G/G | G/G | G/G |
| NSCLC-0872 | 1 | 70 | 1 | 0 | 0 | Non-SCC | G/G | G/G | G/G |
| NSCLC-0873 | 2 | 63 | 0 | 0 | 0 | Non-SCC | G/G | G/G | G/G |
| NSCLC-0874 | 1 | 40 | 0 | 1 | 1 | Non-SCC | G/G | G/G | G/G |
| NSCLC-0875 | 2 | 46 | 0 | 0 | 0 | Non-SCC | G/G | G/G | G/G |
| NSCLC-0876 | 1 | 56 | 1 | 1 | 0 | Non-SCC | G/A | G/A | G/G |
| NSCLC-0877 | 1 | 53 | 1 | 1 | 1 | Non-SCC | G/G | G/G | G/G |
| NSCLC-0878 | 1 | 76 | 1 | 1 | 0 | Non-SCC | G/G | G/G | G/A |
| NSCLC-0879 | 1 | 29 | 0 | 0 | 1 | Non-SCC | G/G | G/G | G/G |
| NSCLC-0880 | 1 | 50 | 1 | 0 | 0 | Non-SCC | G/G | G/G | G/G |
| NSCLC-0881 | 2 | 54 | 0 | 0 | 0 | Non-SCC | G/A | G/G | G/A |
| NSCLC-0882 | 1 | 57 | 0 | 1 | 0 | Non-SCC | G/G | G/G | G/A |
| NSCLC-0883 | 1 | 38 | 1 | 1 | 1 | Non-SCC | G/G | G/G | G/G |
| NSCLC-0884 | 1 | 30 | 0 | 0 | 0 | Non-SCC | G/A | G/G | G/G |
| NSCLC-0885 | 2 | 51 | 0 | 0 | 1 | Non-SCC | G/G | G/G | G/G |
| NSCLC-0886 | 2 | 49 | 0 | 0 | 0 | Non-SCC | G/G | G/G | G/G |
| NSCLC-0887 | 1 | 66 | 0 | 1 | 1 | Non-SCC | G/A | G/A | G/G |
| NSCLC-0888 | 1 | 64 | 1 | 0 | 0 | Non-SCC | G/A | G/A | G/A |
| NSCLC-0889 | 2 | 45 | 0 | 0 | 0 | Non-SCC | G/A | G/A | G/G |
| NSCLC-0890 | 2 | 54 | 0 | 0 | 1 | Non-SCC | G/G | G/G | G/G |
| NSCLC-0891 | 2 | 50 | 0 | 0 | 0 | Non-SCC | G/A | G/A | G/A |
| NSCLC-0892 | 2 | 40 | 0 | 0 | 0 | Non-SCC | G/A | G/A | G/G |
| NSCLC-0893 | 1 | 71 | 1 | 0 | 0 | Non-SCC | G/A | G/A | G/G |
| NSCLC-0894 | 1 | 57 | 1 | 1 | 1 | Non-SCC | G/A | G/A | G/A |
| NSCLC-0895 | 2 | 65 | 0 | 0 | 0 | Non-SCC | G/A | G/A | G/A |
| NSCLC-0896 | 2 | 62 | 0 | 0 | 1 | Non-SCC | G/G | G/G | G/G |
| NSCLC-0897 | 2 | 68 | 0 | 0 | 0 | Non-SCC | G/G | G/G | G/G |
| NSCLC-0898 | 1 | 48 | 0 | 0 | 1 | Non-SCC | G/G | G/G | G/G |
| NSCLC-0899 | 1 | 60 | 1 | 0 | 1 | Non-SCC | G/G | G/A | G/G |
| NSCLC-0900 | 1 | 57 | 0 | 1 | 1 | Non-SCC | G/G | G/G | G/G |
| NSCLC-0901 | 1 | 59 | 0 | 1 | 1 | Non-SCC | G/G | G/G | G/G |
| NSCLC-0902 | 2 | 55 | 0 | 0 | 0 | Non-SCC | G/G | G/G | G/G |
| NSCLC-0903 | 2 | 46 | 0 | 0 | 1 | Non-SCC | G/G | G/G | G/G |
| NSCLC-0904 | 1 | 59 | 1 | 1 | 0 | Non-SCC | G/G | G/G | G/G |
| NSCLC-0905 | 1 | 51 | 1 | 1 | 1 | Non-SCC | G/G | G/G | G/G |
| NSCLC-0906 | 1 | 51 | 1 | 1 | 0 | Non-SCC | G/G | G/G | G/G |
| NSCLC-0907 | 2 | 67 | 0 | 1 | 0 | Non-SCC | G/G | G/G | G/G |
| NSCLC-0908 | 2 | 56 | 0 | 0 | 0 | Non-SCC | G/G | G/G | G/G |
| NSCLC-0909 | 2 | 60 | 0 | 0 | 0 | Non-SCC | G/G | G/G | G/G |
| NSCLC-0910 | 2 | 68 | 0 | 0 | 0 | Non-SCC | G/G | G/G | G/G |
| NSCLC-0911 | 2 | 51 | 0 | 0 | 0 | Non-SCC | G/G | G/G | G/G |
| NSCLC-0912 | 2 | 58 | 0 | 0 | 0 | Non-SCC | G/A | G/A | G/A |
| NSCLC-0913 | 2 | 53 | 0 | 0 | 1 | Non-SCC | G/G | G/G | G/G |
| NSCLC-0914 | 2 | 53 | 0 | 0 | 1 | Non-SCC | G/G | G/G | G/G |
| NSCLC-0915 | 2 | 58 | 0 | 0 | 0 | Non-SCC | G/G | G/G | G/G |
| NSCLC-0916 | 1 | 66 | 1 | 1 | 0 | Non-SCC | G/G | G/A | G/A |
| NSCLC-0917 | 1 | 78 | 0 | 0 | 0 | Non-SCC | G/G | G/G | G/A |
| NSCLC-0918 | 1 | 54 | 1 | 1 | 1 | Non-SCC | G/A | G/A | G/G |
| NSCLC-0919 | 1 | 38 | 0 | 0 | 1 | Non-SCC | G/G | G/G | G/G |
| NSCLC-0920 | 2 | 67 | 0 | 0 | 1 | Non-SCC | G/A | G/G | G/A |
| NSCLC-0921 | 2 | 51 | 0 | 0 | 1 | Non-SCC | G/G | G/G | G/G |
| NSCLC-0922 | 2 | 46 | 0 | 0 | 1 | Non-SCC | G/G | G/G | G/G |
| NSCLC-0923 | 1 | 27 | 0 | 0 | 1 | Non-SCC | A/A | G/A | A/A |
| NSCLC-0924 | 2 | 51 | 0 | 0 | 0 | Non-SCC | G/G | G/G | G/A |
| NSCLC-0925 | 1 | 28 | 1 | 1 | 0 | Non-SCC | G/A | G/A | G/G |
| NSCLC-0926 | 1 | 62 | 1 | 1 | 1 | Non-SCC | G/G | G/G | G/A |
| NSCLC-0927 | 2 | 59 | 0 | 0 | 0 | Non-SCC | G/G | G/G | G/G |
| NSCLC-0928 | 1 | 40 | 0 | 1 | 0 | Non-SCC | G/A | G/G | G/A |
| NSCLC-0929 | 2 | 40 | 0 | 0 | 0 | Non-SCC | G/G | G/G | G/G |
| NSCLC-0930 | 2 | 49 | 0 | 0 | 0 | Non-SCC | G/A | G/A | G/G |
| NSCLC-0931 | 1 | 69 | 1 | 1 | 0 | Non-SCC | G/G | G/G | G/G |
| NSCLC-0932 | 2 | 53 | 0 | 0 | 0 | Non-SCC | G/A | G/G | G/G |
| NSCLC-0933 | 2 | 51 | 0 | 0 | 0 | Non-SCC | G/G | G/G | G/G |
| NSCLC-0934 | 2 | 48 | 0 | 0 | 1 | Non-SCC | G/G | G/G | G/G |
| NSCLC-0935 | 1 | 64 | 1 | 0 | 1 | Non-SCC | G/G | G/G | G/G |
| NSCLC-0936 | 1 | 54 | 1 | 1 | 0 | Non-SCC | G/A | G/A | G/A |
| NSCLC-0937 | 1 | 61 | 0 | 0 | 0 | Non-SCC | G/G | G/G | G/G |
| NSCLC-0938 | 2 | 43 | 0 | 0 | 0 | Non-SCC | G/G | G/G | G/G |
| NSCLC-0939 | 1 | 43 | 1 | 1 | 1 | Non-SCC | G/A | G/A | G/A |
| NSCLC-0940 | 2 | 63 | 0 | 0 | 0 | Non-SCC | G/A | G/G | G/G |
| NSCLC-0941 | 1 | 65 | 1 | 1 | 0 | Non-SCC | G/G | G/G | G/G |
| NSCLC-0942 | 2 | 62 | 0 | 0 | 0 | Non-SCC | G/A | G/A | G/A |
| NSCLC-0943 | 1 | 82 | 1 | 0 | 0 | Non-SCC | G/G | G/G | G/G |
| NSCLC-0944 | 2 | 50 | 0 | 0 | 0 | Non-SCC | G/G | G/G | G/G |
| NSCLC-0945 | 2 | 68 | 0 | 0 | 0 | Non-SCC | G/A | G/G | G/G |
| NSCLC-0946 | 2 | 53 | 0 | 0 | 0 | Non-SCC | G/G | G/G | G/G |
| NSCLC-0947 | 2 | 52 | 0 | 0 | 0 | Non-SCC | G/G | G/A | G/G |
| NSCLC-0948 | 2 | 60 | 0 | 0 | 0 | Non-SCC | G/G | G/G | G/G |
| NSCLC-0949 | 1 | 48 | 1 | 1 | 0 | Non-SCC | G/G | G/G | G/G |
| NSCLC-0950 | 2 | 60 | 0 | 0 | 1 | Non-SCC | G/A | G/A | G/G |
| NSCLC-0951 | 1 | 47 | 0 | 0 | 0 | Non-SCC | G/G | G/G | G/A |
| NSCLC-0952 | 1 | 52 | 1 | 1 | 1 | Non-SCC | G/G | G/G | G/G |
| NSCLC-0953 | 1 | 66 | 1 | 1 | 0 | Non-SCC | G/G | G/G | G/A |
| NSCLC-0954 | 1 | 79 | 1 | 1 | 1 | Non-SCC | G/G | G/G | G/G |
| NSCLC-0955 | 1 | 63 | 1 | 0 | 1 | Non-SCC | G/G | G/G | G/G |
| NSCLC-0956 | 1 | 58 | 1 | 0 | 0 | Non-SCC | G/G | G/G | G/G |
| NSCLC-0957 | 2 | 66 | 0 | 0 | 0 | Non-SCC | G/G | G/G | G/G |
| NSCLC-0958 | 1 | 55 | 1 | 0 | 0 | Non-SCC | G/G | G/G | G/G |
| NSCLC-0959 | 2 | 71 | 0 | 0 | 0 | Non-SCC | G/G | G/G | G/G |
| NSCLC-0960 | 2 | 55 | 0 | 0 | 0 | Non-SCC | G/G | G/G | G/G |
| NSCLC-0961 | 2 | 57 | 0 | 0 | 0 | Non-SCC | G/A | G/A | G/A |
| NSCLC-0962 | 1 | 55 | 1 | 0 | 0 | Non-SCC | G/G | G/G | G/G |
| NSCLC-0963 | 2 | 62 | 0 | 0 | 0 | Non-SCC | G/A | G/A | G/A |
| NSCLC-0964 | 1 | 59 | 1 | 1 | 0 | Non-SCC | G/A | G/G | G/A |
| NSCLC-0965 | 2 | 59 | 0 | 0 | 0 | Non-SCC | G/G | G/G | G/G |
| NSCLC-0966 | 1 | 62 | 1 | 1 | 0 | Non-SCC | G/A | G/A | G/A |
| NSCLC-0967 | 1 | 53 | 1 | 1 | 0 | Non-SCC | G/G | G/G | G/A |
| NSCLC-0968 | 1 | 66 | 1 | 0 | 1 | Non-SCC | G/G | G/G | G/G |
| NSCLC-0969 | 2 | 63 | 0 | 0 | 0 | Non-SCC | G/G | G/G | G/A |
| NSCLC-0970 | 2 | 56 | 0 | 0 | 0 | Non-SCC | G/A | G/G | G/G |
| NSCLC-0971 | 1 | 70 | 0 | 0 | 0 | Non-SCC | A/A | G/G | G/A |
| NSCLC-0972 | 1 | 52 | 0 | 0 | 1 | Non-SCC | G/G | G/G | G/G |
| NSCLC-0973 | 2 | 64 | 1 | 1 | 0 | Non-SCC | G/G | G/G | G/G |
| NSCLC-0974 | 2 | 55 | 0 | 0 | 0 | Non-SCC | G/G | G/G | G/G |
| NSCLC-0975 | 1 | 53 | 1 | 1 | 1 | Non-SCC | G/G | G/G | G/G |
| NSCLC-0976 | 2 | 54 | 0 | 0 | 0 | Non-SCC | G/G | G/G | G/G |
| NSCLC-0977 | 1 | 51 | 1 | 1 | 0 | Non-SCC | G/A | G/G | G/G |
| NSCLC-0978 | 1 | 55 | 1 | 1 | 1 | Non-SCC | G/G | G/G | G/G |
| NSCLC-0979 | 1 | 64 | 1 | 1 | 1 | Non-SCC | A/A | A/A | A/A |
| NSCLC-0980 | 1 | 52 | 1 | 1 | 1 | Non-SCC | G/A | G/G | G/G |
| NSCLC-0981 | 1 | 52 | 0 | 0 | 0 | Non-SCC | G/G | G/G | G/G |
| NSCLC-0982 | 1 | 59 | 0 | 0 | 0 | Non-SCC | G/G | G/G | G/G |
| NSCLC-0983 | 2 | 76 | 0 | 0 | 0 | Non-SCC | G/G | G/G | G/G |
| NSCLC-0984 | 1 | 70 | 0 | 0 | 1 | Non-SCC | G/G | G/G | G/G |
| NSCLC-0985 | 2 | 51 | 0 | 0 | 1 | Non-SCC | G/G | G/G | G/G |
| NSCLC-0986 | 2 | 59 | 0 | 0 | 0 | Non-SCC | G/G | G/G | G/G |
| NSCLC-0987 | 2 | 55 | 0 | 0 | 1 | Non-SCC | G/G | G/G | G/G |
| NSCLC-0988 | 1 | 56 | 0 | 0 | 1 | Non-SCC | G/G | G/G | G/G |
| NSCLC-0989 | 1 | 59 | 1 | 1 | 0 | Non-SCC | G/G | G/G | G/G |
| NSCLC-0990 | 1 | 52 | 1 | 1 | 0 | Non-SCC | G/G | G/G | G/G |
| NSCLC-0991 | 2 | 52 | 0 | 0 | 1 | Non-SCC | G/G | G/G | G/G |
| NSCLC-0992 | 2 | 54 | 0 | 0 | 0 | Non-SCC | G/A | G/A | G/A |
| NSCLC-0993 | 1 | 39 | 1 | 0 | 0 | Non-SCC | G/G | G/G | G/G |
| NSCLC-0994 | 2 | 55 | 0 | 0 | 0 | Non-SCC | G/G | G/G | G/G |
| NSCLC-0995 | 1 | 52 | 1 | 1 | 0 | Non-SCC | G/G | G/G | G/G |
| NSCLC-0996 | 2 | 54 | 0 | 0 | 1 | Non-SCC | G/A | G/G | G/G |
| NSCLC-0997 | 2 | 75 | 0 | 0 | 1 | Non-SCC | G/G | G/G | G/G |
| NSCLC-0998 | 2 | 71 | 0 | 0 | 0 | Non-SCC | G/G | G/G | G/G |
| NSCLC-0999 | 2 | 56 | 0 | 0 | 0 | Non-SCC | G/G | G/G | G/A |
| NSCLC-1000 | 2 | 71 | 0 | 0 | 1 | Non-SCC | G/G | G/G | G/G |
| NSCLC-1001 | 1 | 69 | 1 | 0 | 0 | Non-SCC | G/G | G/G | G/G |
| NSCLC-1002 | 2 | 71 | 0 | 0 | 0 | Non-SCC | G/G | G/G | G/G |
| NSCLC-1003 | 2 | 60 | 0 | 0 | 0 | Non-SCC | G/G | G/G | G/G |
| NSCLC-1004 | 2 | 53 | 0 | 0 | 1 | Non-SCC | G/G | G/G | G/G |
| NSCLC-1005 | 2 | 57 | 0 | 0 | 0 | Non-SCC | G/G | G/G | G/G |
| NSCLC-1006 | 1 | 69 | 1 | 1 | 1 | Non-SCC | G/G | G/G | G/G |
| NSCLC-1007 | 2 | 67 | 0 | 0 | 0 | Non-SCC | G/G | G/G | G/G |
| NSCLC-1008 | 2 | 53 | 0 | 0 | 0 | Non-SCC | G/G | G/G | G/G |
| NSCLC-1009 | 2 | 52 | 0 | 0 | 0 | Non-SCC | G/G | G/A | G/G |
| NSCLC-1010 | 2 | 62 | 0 | 0 | 1 | Non-SCC | G/A | G/G | G/A |
| NSCLC-1011 | 2 | 65 | 0 | 0 | 0 | Non-SCC | G/G | G/G | G/G |
| NSCLC-1012 | 1 | 49 | 1 | 1 | 0 | Non-SCC | G/G | G/G | G/G |
| NSCLC-1013 | 2 | 59 | 0 | 0 | 1 | Non-SCC | G/G | G/G | G/G |
| NSCLC-1014 | 2 | 46 | 0 | 0 | 0 | Non-SCC | G/A | G/G | G/G |
| NSCLC-1015 | 2 | 67 | 0 | 0 | 0 | Non-SCC | G/G | G/G | G/G |
| NSCLC-1016 | 1 | 55 | 0 | 0 | 0 | Non-SCC | G/G | G/G | G/A |
| NSCLC-1017 | 1 | 76 | 0 | 0 | 0 | Non-SCC | G/G | G/G | G/G |
| NSCLC-1018 | 1 | 60 | 0 | 0 | 0 | Non-SCC | G/A | G/A | G/A |
| NSCLC-1019 | 1 | 67 | 1 | 1 | 0 | Non-SCC | G/G | G/G | G/G |
| NSCLC-1020 | 2 | 63 | 0 | 0 | 0 | Non-SCC | G/G | G/G | G/A |
| NSCLC-1021 | 1 | 60 | 0 | 1 | 1 | Non-SCC | G/G | G/G | G/G |
| NSCLC-1022 | 1 | 59 | 0 | 0 | 1 | Non-SCC | G/A | G/G | G/G |
| NSCLC-1023 | 2 | 57 | 0 | 0 | 0 | Non-SCC | G/G | G/A | G/G |
| NSCLC-1024 | 2 | 56 | 0 | 0 | 1 | Non-SCC | G/G | G/G | G/G |
| NSCLC-1025 | 1 | 60 | 0 | 0 | 0 | Non-SCC | G/G | G/G | G/G |
| NSCLC-1026 | 2 | 72 | 0 | 0 | 0 | Non-SCC | G/G | G/G | G/G |
| NSCLC-1027 | 2 | 73 | 0 | 0 | 1 | Non-SCC | G/A | G/G | G/A |
| NSCLC-1028 | 2 | 59 | 0 | 0 | 1 | Non-SCC | G/G | G/G | G/G |
| NSCLC-1029 | 2 | 54 | 0 | 0 | 1 | Non-SCC | G/G | G/G | G/G |
| NSCLC-1030 | 1 | 62 | 1 | 1 | 0 | Non-SCC | G/A | G/A | G/A |
| NSCLC-1031 | 2 | 62 | 0 | 0 | 0 | Non-SCC | G/A | G/G | G/G |
| NSCLC-1032 | 1 | 75 | 0 | 0 | 1 | Non-SCC | G/A | G/A | G/G |
| NSCLC-1033 | 2 | 72 | 0 | 0 | 0 | Non-SCC | G/G | G/G | G/A |
| NSCLC-1034 | 1 | 46 | 1 | 1 | 1 | Non-SCC | G/G | G/G | G/G |
| NSCLC-1035 | 1 | 58 | 1 | 0 | 1 | Non-SCC | G/A | G/A | G/G |
| NSCLC-1036 | 2 | 66 | 0 | 0 | 1 | Non-SCC | G/G | G/G | G/G |
| NSCLC-1037 | 2 | 64 | 0 | 0 | 0 | Non-SCC | G/G | G/G | G/G |
| NSCLC-1038 | 2 | 53 | 0 | 0 | 1 | Non-SCC | G/A | G/G | G/G |
| NSCLC-1039 | 1 | 62 | 0 | 0 | 1 | Non-SCC | G/G | G/G | G/A |
| NSCLC-1040 | 1 | 63 | 0 | 0 | 1 | Non-SCC | G/G | G/G | G/G |
| NSCLC-1041 | 2 | 60 | 0 | 0 | 1 | Non-SCC | G/G | G/G | G/G |
| NSCLC-1042 | 1 | 57 | 0 | 0 | 0 | Non-SCC | G/G | G/G | G/A |
| NSCLC-1043 | 2 | 67 | 0 | 0 | 1 | Non-SCC | G/G | G/G | G/G |
| NSCLC-1044 | 2 | 54 | 0 | 0 | 1 | Non-SCC | G/G | G/G | G/G |
| NSCLC-1045 | 2 | 79 | 0 | 0 | 0 | Non-SCC | G/G | G/G | G/A |
| NSCLC-1046 | 1 | 63 | 1 | 0 | 1 | Non-SCC | G/A | G/G | G/G |
| NSCLC-1047 | 2 | 69 | 0 | 0 | 1 | Non-SCC | G/G | G/G | G/G |
| NSCLC-1048 | 1 | 69 | 1 | 1 | 0 | Non-SCC | G/A | G/A | A/A |
| NSCLC-1049 | 1 | 45 | 0 | 0 | 0 | Non-SCC | G/G | G/G | G/G |
| NSCLC-1050 | 2 | 69 | 0 | 0 | 1 | Non-SCC | G/A | G/A | G/G |
| NSCLC-1051 | 1 | 62 | 1 | 0 | 0 | Non-SCC | G/G | G/G | G/G |
| NSCLC-1052 | 1 | 55 | 1 | 0 | 0 | Non-SCC | G/A | G/A | G/G |
| NSCLC-1053 | 2 | 55 | 0 | 0 | 1 | Non-SCC | G/G | G/G | G/G |
| NSCLC-1054 | 2 | 50 | 0 | 0 | 1 | Non-SCC | G/G | G/G | G/G |
| NSCLC-1055 | 2 | 65 | 0 | 0 | 0 | Non-SCC | G/G | G/G | G/A |
| NSCLC-1056 | 2 | 62 | 0 | 0 | 0 | Non-SCC | G/G | G/G | G/G |
| NSCLC-1057 | 1 | 67 | 1 | 0 | 1 | Non-SCC | G/G | G/A | G/G |
| NSCLC-1058 | 2 | 75 | 0 | 0 | 1 | Non-SCC | G/A | G/G | G/G |
| NSCLC-1059 | 1 | 63 | 0 | 0 | 1 | Non-SCC | G/A | G/A | G/G |
| NSCLC-1060 | 2 | 66 | 0 | 0 | 0 | Non-SCC | G/A | G/A | G/A |
| NSCLC-1061 | 1 | 66 | 1 | 1 | 0 | Non-SCC | G/G | G/G | G/G |
| NSCLC-1062 | 2 | 54 | 0 | 0 | 0 | Non-SCC | G/G | G/G | G/G |
| NSCLC-1063 | 1 | 60 | 0 | 0 | 0 | Non-SCC | G/G | G/G | G/G |
| NSCLC-1064 | 1 | 75 | 1 | 1 | 0 | Non-SCC | G/G | G/G | G/G |
| NSCLC-1065 | 1 | 49 | 0 | 0 | 1 | Non-SCC | G/A | G/A | G/A |
| NSCLC-1066 | 2 | 60 | 0 | 0 | 1 | Non-SCC | G/A | G/A | G/G |
| NSCLC-1067 | 1 | 62 | 0 | 0 | 0 | Non-SCC | G/G | G/G | G/G |
| NSCLC-1068 | 2 | 53 | 0 | 0 | 0 | Non-SCC | G/A | G/G | G/A |
| NSCLC-1069 | 2 | 58 | 0 | 0 | 0 | Non-SCC | G/G | G/G | G/G |
| NSCLC-1070 | 2 | 58 | 0 | 0 | 0 | Non-SCC | A/A | A/A | A/A |
| NSCLC-1071 | 2 | 64 | 0 | 0 | 0 | Non-SCC | G/G | G/G | G/A |
| NSCLC-1072 | 2 | 54 | 0 | 0 | 1 | Non-SCC | G/G | G/G | G/G |
| NSCLC-1073 | 1 | 72 | 0 | 0 | 0 | Non-SCC | G/A | G/A | G/G |
| NSCLC-1074 | 1 | 62 | 0 | 0 | 0 | Non-SCC | G/A | G/A | G/A |
| NSCLC-1075 | 1 | 64 | 0 | 0 | 1 | Non-SCC | G/G | G/G | G/G |
| NSCLC-1076 | 2 | 64 | 0 | 0 | 0 | Non-SCC | A/A | G/A | G/G |
| NSCLC-1077 | 1 | 65 | 0 | 0 | 0 | Non-SCC | G/G | G/G | G/G |
| NSCLC-1078 | 1 | 66 | 1 | 0 | 0 | Non-SCC | G/G | G/G | G/G |
| NSCLC-1079 | 2 | 74 | 0 | 0 | 0 | Non-SCC | G/G | G/G | G/G |
| NSCLC-1080 | 1 | 68 | 0 | 0 | 0 | Non-SCC | G/G | G/G | G/G |
| NSCLC-1081 | 2 | 49 | 0 | 0 | 0 | Non-SCC | G/G | G/G | G/G |
| NSCLC-1082 | 1 | 69 | 1 | 0 | 0 | Non-SCC | G/A | G/A | A/A |
| NSCLC-1083 | 1 | 50 | 1 | 0 | 1 | Non-SCC | G/G | G/G | G/G |
| NSCLC-1084 | 1 | 66 | 0 | 0 | 1 | Non-SCC | G/A | G/A | G/G |
| NSCLC-1085 | 1 | 53 | 1 | 0 | 0 | Non-SCC | G/G | G/G | G/G |
| NSCLC-1086 | 1 | 69 | 0 | 0 | 0 | Non-SCC | G/G | G/G | G/G |
| NSCLC-1087 | 2 | 60 | 0 | 0 | 1 | Non-SCC | G/G | G/G | G/G |
| NSCLC-1088 | 1 | 63 | 1 | 1 | 1 | Non-SCC | G/A | G/G | G/G |
| NSCLC-1089 | 1 | 58 | 1 | 1 | 0 | Non-SCC | G/A | G/G | G/G |
| NSCLC-1090 | 1 | 69 | 0 | 0 | 1 | Non-SCC | G/A | G/A | G/A |
| NSCLC-1091 | 2 | 70 | 0 | 0 | 0 | Non-SCC | G/G | G/G | G/G |
| NSCLC-1092 | 2 | 46 | 0 | 0 | 1 | Non-SCC | G/A | G/G | G/A |
| NSCLC-1093 | 2 | 49 | 0 | 0 | 0 | Non-SCC | G/G | G/G | G/G |
| NSCLC-1094 | 2 | 59 | 0 | 0 | 0 | Non-SCC | G/G | G/G | G/G |
| NSCLC-1095 | 1 | 63 | 0 | 0 | 1 | Non-SCC | G/G | G/G | G/G |
| NSCLC-1096 | 1 | 67 | 1 | 1 | 0 | Non-SCC | A/A | G/A | G/G |
| NSCLC-1097 | 1 | 58 | 0 | 0 | 1 | Non-SCC | G/G | G/G | G/G |
| NSCLC-1098 | 2 | 63 | 0 | 0 | 1 | Non-SCC | G/A | G/G | G/G |
| NSCLC-1099 | 1 | 37 | 1 | 1 | 0 | Non-SCC | G/G | G/G | G/A |
| NSCLC-1100 | 1 | 50 | 1 | 1 | 0 | Non-SCC | G/G | G/G | G/G |
| NSCLC-1101 | 2 | 44 | 0 | 0 | 0 | Non-SCC | G/G | G/G | G/G |
| NSCLC-1102 | 2 | 55 | 0 | 0 | 0 | Non-SCC | G/A | G/A | G/A |
| NSCLC-1103 | 1 | 57 | 1 | 1 | 0 | Non-SCC | G/A | G/A | G/A |
| NSCLC-1104 | 1 | 51 | 1 | 0 | 1 | Non-SCC | G/G | G/G | G/G |
| NSCLC-1105 | 1 | 53 | 0 | 0 | 1 | Non-SCC | G/G | G/G | G/G |
| NSCLC-1106 | 1 | 60 | 1 | 0 | 0 | Non-SCC | G/G | G/G | G/G |
| NSCLC-1107 | 2 | 37 | 0 | 0 | 0 | Non-SCC | G/G | G/G | G/G |
| NSCLC-1108 | 2 | 66 | 0 | 0 | 0 | Non-SCC | G/G | G/G | G/G |
| NSCLC-1109 | 1 | 66 | 0 | 0 | 0 | Non-SCC | G/G | G/G | G/G |
| NSCLC-1110 | 1 | 55 | 0 | 0 | 0 | Non-SCC | G/G | G/G | G/G |
| NSCLC-1111 | 2 | 52 | 0 | 0 | 0 | Non-SCC | G/G | G/G | G/G |
| NSCLC-1112 | 1 | 52 | 1 | 1 | 0 | Non-SCC | G/G | G/G | G/G |
| NSCLC-1113 | 2 | 46 | 0 | 0 | 0 | Non-SCC | G/G | G/G | G/G |
| NSCLC-1114 | 2 | 77 | 0 | 0 | 1 | Non-SCC | G/G | G/G | G/G |
| NSCLC-1115 | 2 | 47 | 0 | 0 | 0 | Non-SCC | G/A | G/G | G/G |
| NSCLC-1116 | 2 | 51 | 0 | 0 | 0 | Non-SCC | G/A | G/G | G/A |
| NSCLC-1117 | 1 | 42 | 0 | 0 | 0 | Non-SCC | G/G | G/A | G/G |
| NSCLC-1118 | 2 | 55 | 0 | 0 | 1 | Non-SCC | G/G | G/A | G/G |
| NSCLC-1119 | 2 | 65 | 0 | 0 | 0 | Non-SCC | G/A | G/G | G/G |
| NSCLC-1120 | 1 | 49 | 0 | 0 | 0 | Non-SCC | G/A | G/G | G/A |
| NSCLC-1121 | 2 | 48 | 0 | 0 | 0 | Non-SCC | G/G | G/G | G/G |
| NSCLC-1122 | 1 | 77 | 0 | 0 | 0 | Non-SCC | G/G | G/G | G/G |
| NSCLC-1123 | 1 | 76 | 1 | 1 | 1 | Non-SCC | G/A | G/G | G/G |
| NSCLC-1124 | 1 | 57 | 0 | 1 | 1 | Non-SCC | G/G | G/G | G/G |
| NSCLC-1125 | 1 | 63 | 1 | 1 | 1 | Non-SCC | G/A | G/G | G/G |
| NSCLC-1126 | 1 | 53 | 0 | 0 | 1 | Non-SCC | G/G | G/G | G/G |
| NSCLC-1127 | 1 | 51 | 1 | 0 | 1 | Non-SCC | G/G | G/G | G/G |
| NSCLC-1128 | 2 | 48 | 0 | 0 | 0 | Non-SCC | G/A | G/G | G/A |
| NSCLC-1129 | 1 | 70 | 1 | 0 | 0 | Non-SCC | G/G | G/G | G/G |
| NSCLC-1130 | 2 | 44 | 0 | 0 | 0 | Non-SCC | G/G | G/G | G/G |
| NSCLC-1131 | 1 | 70 | 0 | 0 | 0 | Non-SCC | G/A | G/A | G/G |
| NSCLC-1132 | 2 | 58 | 0 | 0 | 0 | Non-SCC | G/G | G/G | G/G |
| NSCLC-1133 | 1 | 55 | 0 | 0 | 0 | Non-SCC | G/G | G/G | G/A |
| NSCLC-1134 | 2 | 61 | 0 | 0 | 0 | Non-SCC | G/A | G/A | G/A |
| NSCLC-1135 | 1 | 48 | 1 | 1 | 1 | Non-SCC | G/G | G/G | G/G |
| NSCLC-1136 | 1 | 43 | 0 | 0 | 0 | Non-SCC | ? | G/G | ? |
| NSCLC-1137 | 1 | 59 | 1 | 0 | 0 | Non-SCC | G/G | G/G | G/G |
| NSCLC-1138 | 1 | 46 | 1 | 1 | 1 | Non-SCC | G/G | G/G | G/G |
| NSCLC-1139 | 2 | 61 | 0 | 0 | 0 | Non-SCC | G/A | G/A | G/A |
| NSCLC-1140 | 1 | 68 | 1 | 0 | 1 | Non-SCC | G/G | G/G | G/G |
| NSCLC-1141 | 1 | 60 | 1 | 1 | 1 | Non-SCC | G/G | G/G | G/G |
| NSCLC-1142 | 2 | 68 | 0 | 0 | 1 | Non-SCC | G/G | G/G | G/G |
| NSCLC-1143 | 2 | 65 | 0 | 0 | 0 | Non-SCC | A/A | G/A | G/G |
| NSCLC-1144 | 2 | 62 | 0 | 0 | 1 | Non-SCC | G/G | G/G | G/G |
| NSCLC-1145 | 1 | 70 | 1 | 1 | 0 | Non-SCC | ? | ? | ? |
| NSCLC-1146 | 1 | 70 | 1 | 0 | 0 | Non-SCC | G/G | G/G | G/G |
| NSCLC-1147 | 2 | 39 | 0 | 0 | 0 | Non-SCC | G/G | G/G | G/G |
| NSCLC-1148 | 1 | 57 | 0 | 0 | 1 | Non-SCC | G/G | G/G | G/G |
| NSCLC-1149 | 2 | 68 | 0 | 0 | 1 | Non-SCC | G/G | G/G | G/G |
| NSCLC-1150 | 2 | 57 | 0 | 0 | 0 | Non-SCC | G/A | G/A | G/A |
| NSCLC-1151 | 1 | 64 | 1 | 1 | 0 | Non-SCC | G/G | G/G | G/G |
| NSCLC-1152 | 1 | 65 | 1 | 1 | 0 | Non-SCC | G/G | G/G | G/G |
| NSCLC-1153 | 2 | 45 | 0 | 0 | 0 | Non-SCC | ? | G/G | ? |
| NSCLC-1154 | 2 | 33 | 0 | 0 | 0 | Non-SCC | G/G | G/G | G/G |
| NSCLC-1155 | 1 | 29 | 0 | 0 | 0 | Non-SCC | G/G | G/G | G/G |
| NSCLC-1156 | 2 | 66 | 0 | 0 | 0 | Non-SCC | G/G | G/G | G/G |
| NSCLC-1157 | 2 | 82 | 0 | 0 | 0 | Non-SCC | ? | ? | ? |
| NSCLC-1158 | 1 | 62 | 0 | 0 | 1 | Non-SCC | G/G | G/G | G/G |
| NSCLC-1159 | 2 | 50 | 0 | 0 | 0 | Non-SCC | ? | ? | ? |
| NSCLC-1160 | 1 | 51 | 1 | 0 | 0 | Non-SCC | G/G | G/G | G/A |
| NSCLC-1161 | 2 | 55 | 0 | 0 | 0 | Non-SCC | G/G | G/G | G/G |
| NSCLC-1162 | 1 | 51 | 1 | 1 | 1 | Non-SCC | G/G | G/G | G/G |
| NSCLC-1163 | 2 | 66 | 0 | 0 | 1 | Non-SCC | G/G | G/G | G/G |
| NSCLC-1164 | 2 | 48 | 0 | 0 | 0 | Non-SCC | G/A | G/G | G/G |
| NSCLC-1165 | 2 | 71 | 0 | 0 | 1 | Non-SCC | G/G | G/G | G/G |
| NSCLC-1166 | 2 | 47 | 0 | 0 | 0 | Non-SCC | G/G | G/G | G/G |
| NSCLC-1167 | 1 | 51 | 1 | 0 | 1 | Non-SCC | G/G | G/G | G/G |
| NSCLC-1168 | 2 | 45 | 0 | 0 | 1 | Non-SCC | G/G | G/G | G/G |
| NSCLC-1169 | 1 | 57 | 0 | 0 | 0 | Non-SCC | G/G | G/G | G/A |
| NSCLC-1170 | 2 | 54 | 0 | 0 | 0 | Non-SCC | G/G | G/G | G/G |
| NSCLC-1171 | 2 | 61 | 0 | 0 | 0 | Non-SCC | G/A | G/A | G/A |
| NSCLC-1172 | 1 | 73 | 1 | 1 | 0 | Non-SCC | G/G | G/G | G/G |
| NSCLC-1173 | 1 | 29 | 0 | 0 | 0 | Non-SCC | G/A | G/G | G/A |
| NSCLC-1174 | 2 | 62 | 0 | 0 | 0 | Non-SCC | G/G | G/G | G/G |
| NSCLC-1175 | 2 | 29 | 0 | 0 | 0 | Non-SCC | G/G | G/G | G/G |
| NSCLC-1176 | 2 | 54 | 0 | 0 | 1 | Non-SCC | G/A | G/A | G/G |
| NSCLC-1177 | 2 | 73 | 0 | 0 | 0 | Non-SCC | G/G | G/G | G/G |
| NSCLC-1178 | 2 | 54 | 0 | 0 | 0 | Non-SCC | G/G | G/G | G/G |
| NSCLC-1179 | 1 | 53 | 1 | 0 | 0 | Non-SCC | G/G | G/A | G/G |
| NSCLC-1180 | 1 | 62 | 1 | 0 | 0 | Non-SCC | G/G | G/G | G/G |
| NSCLC-1181 | 2 | 67 | 0 | 0 | 0 | Non-SCC | G/G | G/G | G/G |
| NSCLC-1182 | 2 | 45 | 0 | 0 | 0 | Non-SCC | G/A | G/A | G/G |
| NSCLC-1183 | 1 | 65 | 1 | 1 | 0 | Non-SCC | G/G | G/G | G/G |
| NSCLC-1184 | 1 | 54 | 1 | 0 | 0 | Non-SCC | G/G | G/G | G/G |
| NSCLC-1185 | 1 | 51 | 1 | 1 | 0 | Non-SCC | G/G | G/G | G/G |
| NSCLC-1186 | 1 | 60 | 0 | 0 | 0 | Non-SCC | G/G | G/G | G/G |
| NSCLC-1187 | 1 | 52 | 0 | 0 | 0 | Non-SCC | G/G | G/A | G/G |
| NSCLC-1188 | 1 | 71 | 1 | 0 | 0 | Non-SCC | G/G | G/G | G/G |
| NSCLC-1189 | 1 | 64 | 1 | 1 | 1 | Non-SCC | G/G | G/G | G/G |
| NSCLC-1190 | 2 | 67 | 0 | 0 | 1 | Non-SCC | G/G | G/G | G/G |
| NSCLC-1191 | 2 | 63 | 0 | 0 | 0 | Non-SCC | G/G | G/G | G/G |
| NSCLC-1192 | 2 | 29 | 0 | 0 | 0 | Non-SCC | G/A | G/G | G/A |
| NSCLC-1193 | 2 | 56 | 0 | 0 | 0 | Non-SCC | G/A | G/A | G/G |
| Control-0001 | 1 | 55 | 0 | 0 | 1 |  | G/G | G/G | G/G |
| Control-0002 | 2 | 53 | 0 | 0 | 1 |  | G/A | G/A | G/G |
| Control-0003 | 1 | 59 | 0 | 0 | 1 |  | G/A | G/A | G/A |
| Control-0004 | 2 | 54 | 0 | 0 | 0 |  | G/G | G/A | G/G |
| Control-0005 | 2 | 70 | 0 | 0 | 1 |  | G/G | G/G | G/G |
| Control-0006 | 2 | 56 | 0 | 0 | 0 |  | G/A | G/A | G/G |
| Control-0007 | 1 | 51 | 0 | 0 | 1 |  | G/G | G/G | G/G |
| Control-0008 | 2 | 61 | 0 | 0 | 0 |  | G/G | G/G | G/G |
| Control-0009 | 1 | 57 | 1 | 0 | 1 |  | G/A | A/A | G/A |
| Control-0010 | 1 | 53 | 1 | 0 | 0 |  | G/A | G/A | G/A |
| Control-0011 | 1 | 56 | 0 | 1 | 1 |  | G/A | G/A | G/A |
| Control-0012 | 1 | 64 | 1 | 1 | 1 |  | G/A | G/A | G/G |
| Control-0013 | 1 | 62 | 0 | 0 | 1 |  | G/G | G/G | G/G |
| Control-0014 | 1 | 66 | 0 | 0 | 1 |  | G/G | G/G | G/G |
| Control-0015 | 1 | 29 | 1 | 0 | 1 |  | G/G | G/A | G/G |
| Control-0016 | 1 | 38 | 0 | 0 | 1 |  | G/G | G/G | G/A |
| Control-0017 | 1 | 60 | 1 | 0 | 1 |  | G/G | G/G | G/G |
| Control-0018 | 2 | 53 | 0 | 0 | 1 |  | G/A | G/A | G/G |
| Control-0019 | 1 | 58 | 0 | 0 | 0 |  | G/G | G/G | G/G |
| Control-0020 | 2 | 52 | 0 | 0 | 0 |  | G/G | G/G | G/G |
| Control-0021 | 2 | 38 | 0 | 0 | 0 |  | G/G | G/G | G/G |
| Control-0022 | 1 | 28 | 0 | 0 | 0 |  | G/G | G/G | G/G |
| Control-0023 | 2 | 65 | 0 | 0 | 1 |  | G/A | G/A | G/A |
| Control-0024 | 2 | 52 | 0 | 0 | 0 |  | G/G | G/G | G/G |
| Control-0025 | 2 | 46 | 0 | 0 | 0 |  | G/G | G/G | G/G |
| Control-0026 | 1 | 56 | 0 | 0 | 1 |  | G/A | G/A | G/G |
| Control-0027 | 2 | 68 | 0 | 0 | 1 |  | G/G | G/G | G/G |
| Control-0028 | 1 | 59 | 0 | 0 | 0 |  | G/A | G/G | G/G |
| Control-0029 | 1 | 62 | 0 | 0 | 1 |  | G/G | G/A | G/G |
| Control-0030 | 1 | 71 | 0 | 0 | 0 |  | G/G | G/G | G/G |
| Control-0031 | 1 | 60 | 1 | 1 | 0 |  | G/G | G/G | G/A |
| Control-0032 | 1 | 56 | 1 | 0 | 0 |  | G/A | G/G | G/G |
| Control-0033 | 1 | 60 | 0 | 0 | 0 |  | G/G | G/G | G/G |
| Control-0034 | 2 | 72 | 0 | 0 | 0 |  | G/G | G/G | G/G |
| Control-0035 | 2 | 61 | 0 | 0 | 0 |  | G/A | G/G | G/A |
| Control-0036 | 2 | 66 | 0 | 0 | 0 |  | G/G | G/G | G/G |
| Control-0037 | 1 | 61 | 1 | 0 | 0 |  | G/G | G/G | G/G |
| Control-0038 | 2 | 54 | 0 | 0 | 0 |  | G/G | G/G | G/G |
| Control-0039 | 2 | 56 | 0 | 0 | 0 |  | G/A | G/G | G/G |
| Control-0040 | 2 | 71 | 0 | 0 | 1 |  | G/A | G/A | G/G |
| Control-0041 | 1 | 72 | 0 | 0 | 0 |  | G/A | G/G | G/A |
| Control-0042 | 1 | 46 | 0 | 0 | 0 |  | G/G | G/G | G/G |
| Control-0043 | 1 | 62 | 1 | 0 | 0 |  | G/G | G/G | G/G |
| Control-0044 | 2 | 59 | 0 | 0 | 0 |  | G/G | G/G | G/G |
| Control-0045 | 2 | 60 | 0 | 0 | 1 |  | G/G | G/G | G/G |
| Control-0046 | 2 | 52 | 0 | 0 | 1 |  | G/A | G/G | G/G |
| Control-0047 | 1 | 56 | 1 | 0 | 0 |  | G/A | G/A | G/A |
| Control-0048 | 1 | 59 | 1 | 0 | 0 |  | G/G | G/G | G/G |
| Control-0049 | 1 | 61 | 0 | 0 | 1 |  | G/A | A/A | G/A |
| Control-0050 | 1 | 63 | 0 | 0 | 1 |  | G/A | G/G | G/A |
| Control-0051 | 1 | 72 | 0 | 0 | 0 |  | G/A | G/A | G/A |
| Control-0052 | 1 | 64 | 0 | 1 | 0 |  | G/A | G/A | G/A |
| Control-0053 | 2 | 67 | 0 | 0 | 1 |  | G/G | G/G | G/G |
| Control-0054 | 1 | 58 | 1 | 0 | 0 |  | G/G | G/G | G/A |
| Control-0055 | 2 | 61 | 0 | 0 | 0 |  | G/G | G/G | G/G |
| Control-0056 | 1 | 41 | 1 | 0 | 1 |  | G/A | G/G | G/G |
| Control-0057 | 2 | 69 | 0 | 0 | 0 |  | G/A | G/A | G/G |
| Control-0058 | 1 | 62 | 0 | 0 | 0 |  | G/G | G/G | G/G |
| Control-0059 | 1 | 81 | 0 | 0 | 0 |  | G/G | G/G | G/G |
| Control-0060 | 1 | 57 | 0 | 0 | 0 |  | G/G | G/G | G/A |
| Control-0061 | 2 | 51 | 0 | 0 | 1 |  | G/G | G/G | G/G |
| Control-0062 | 1 | 55 | 1 | 0 | 0 |  | G/G | G/G | G/A |
| Control-0063 | 2 | 37 | 0 | 0 | 0 |  | G/A | G/A | G/A |
| Control-0064 | 1 | 65 | 1 | 1 | 0 |  | G/G | G/G | G/G |
| Control-0065 | 1 | 57 | 0 | 0 | 1 |  | G/G | ? | G/G |
| Control-0066 | 1 | 80 | 0 | 0 | 0 |  | G/G | G/G | G/A |
| Control-0067 | 2 | 49 | 0 | 0 | 0 |  | G/G | G/G | G/G |
| Control-0068 | 2 | 46 | 0 | 0 | 0 |  | G/G | G/G | G/A |
| Control-0069 | 2 | 64 | 0 | 0 | 1 |  | G/G | G/A | G/G |
| Control-0070 | 2 | 65 | 0 | 0 | 0 |  | G/G | G/G | G/G |
| Control-0071 | 1 | 78 | 0 | 0 | 1 |  | G/G | G/G | G/G |
| Control-0072 | 1 | 43 | 0 | 0 | 0 |  | G/G | G/A | G/G |
| Control-0073 | 1 | 64 | 0 | 0 | 0 |  | G/A | G/A | G/G |
| Control-0074 | 2 | 56 | 0 | 0 | 1 |  | G/G | G/G | G/G |
| Control-0075 | 2 | 52 | 0 | 0 | 1 |  | G/G | G/G | G/G |
| Control-0076 | 2 | 57 | 0 | 0 | 0 |  | G/G | G/G | G/G |
| Control-0077 | 2 | 67 | 0 | 0 | 0 |  | G/G | G/G | G/G |
| Control-0078 | 2 | 53 | 0 | 0 | 0 |  | G/G | G/G | G/G |
| Control-0079 | 1 | 48 | 1 | 0 | 1 |  | G/G | G/G | G/G |
| Control-0080 | 1 | 57 | 1 | 0 | 0 |  | G/G | G/G | G/G |
| Control-0081 | 1 | 62 | 1 | 0 | 1 |  | G/G | G/G | G/G |
| Control-0082 | 2 | 71 | 0 | 0 | 0 |  | G/A | G/G | G/G |
| Control-0083 | 2 | 53 | 0 | 0 | 0 |  | G/G | G/G | G/G |
| Control-0084 | 2 | 49 | 0 | 0 | 1 |  | G/G | G/G | G/G |
| Control-0085 | 2 | 61 | 0 | 0 | 0 |  | G/G | G/G | G/A |
| Control-0086 | 2 | 62 | 0 | 0 | 1 |  | A/A | G/A | G/A |
| Control-0087 | 1 | 53 | 1 | 0 | 1 |  | G/G | G/G | G/G |
| Control-0088 | 1 | 62 | 0 | 0 | 1 |  | G/A | G/A | G/A |
| Control-0089 | 1 | 63 | 0 | 0 | 0 |  | G/G | G/G | G/A |
| Control-0090 | 1 | 33 | 0 | 0 | 0 |  | G/G | G/G | G/G |
| Control-0091 | 1 | 81 | 0 | 0 | 1 |  | G/G | G/A | G/G |
| Control-0092 | 2 | 59 | 0 | 0 | 1 |  | G/A | G/A | G/A |
| Control-0093 | 2 | 70 | 0 | 0 | 0 |  | G/G | G/G | G/G |
| Control-0094 | 2 | 56 | 0 | 0 | 0 |  | G/A | G/A | G/A |
| Control-0095 | 1 | 58 | 0 | 0 | 0 |  | G/G | G/G | G/G |
| Control-0096 | 1 | 60 | 1 | 0 | 0 |  | G/A | G/A | G/G |
| Control-0097 | 1 | 59 | 0 | 0 | 1 |  | G/A | G/G | G/G |
| Control-0098 | 1 | 76 | 1 | 0 | 0 |  | G/A | G/A | A/A |
| Control-0099 | 1 | 52 | 0 | 0 | 0 |  | G/G | G/G | G/G |
| Control-0100 | 2 | 34 | 0 | 0 | 0 |  | G/A | G/A | G/G |
| Control-0101 | 1 | 60 | 1 | 0 | 1 |  | G/G | G/G | G/G |
| Control-0102 | 1 | 76 | 1 | 0 | 0 |  | G/G | G/G | G/G |
| Control-0103 | 2 | 50 | 0 | 0 | 1 |  | G/G | G/G | G/G |
| Control-0104 | 1 | 57 | 0 | 0 | 0 |  | G/A | G/A | G/A |
| Control-0105 | 1 | 51 | 0 | 0 | 0 |  | G/G | G/G | G/G |
| Control-0106 | 1 | 60 | 0 | 0 | 0 |  | G/G | G/G | G/G |
| Control-0107 | 2 | 65 | 0 | 0 | 1 |  | G/G | G/G | G/G |
| Control-0108 | 1 | 55 | 0 | 0 | 1 |  | G/G | G/G | G/G |
| Control-0109 | 1 | 38 | 1 | 0 | 0 |  | G/G | G/G | G/G |
| Control-0110 | 1 | 64 | 0 | 0 | 1 |  | G/A | G/A | G/G |
| Control-0111 | 1 | 62 | 0 | 0 | 0 |  | G/G | G/G | G/A |
| Control-0112 | 1 | 54 | 1 | 0 | 0 |  | G/G | G/G | G/G |
| Control-0113 | 1 | 64 | 0 | 0 | 0 |  | G/A | G/A | G/A |
| Control-0114 | 1 | 56 | 0 | 0 | 0 |  | G/G | G/G | G/G |
| Control-0115 | 2 | 60 | 0 | 0 | 1 |  | G/G | G/G | G/A |
| Control-0116 | 1 | 64 | 0 | 0 | 0 |  | G/A | G/A | G/A |
| Control-0117 | 1 | 58 | 0 | 0 | 0 |  | G/G | G/G | G/G |
| Control-0118 | 1 | 46 | 0 | 0 | 0 |  | G/G | G/G | G/A |
| Control-0119 | 2 | 72 | 0 | 0 | 0 |  | A/A | A/A | G/A |
| Control-0120 | 1 | 44 | 1 | 0 | 1 |  | G/G | G/G | G/G |
| Control-0121 | 2 | 41 | 0 | 0 | 0 |  | G/G | G/G | G/G |
| Control-0122 | 2 | 67 | 0 | 0 | 0 |  | G/A | G/A | G/A |
| Control-0123 | 2 | 65 | 0 | 0 | 0 |  | G/G | G/G | G/G |
| Control-0124 | 2 | 66 | 0 | 0 | 1 |  | G/G | G/G | G/G |
| Control-0125 | 1 | 61 | 1 | 0 | 0 |  | G/G | G/G | G/G |
| Control-0126 | 1 | 52 | 0 | 0 | 0 |  | A/A | G/A | G/A |
| Control-0127 | 2 | 67 | 0 | 0 | 1 |  | G/A | G/A | G/A |
| Control-0128 | 1 | 60 | 0 | 0 | 1 |  | G/G | G/G | G/G |
| Control-0129 | 1 | 56 | 1 | 0 | 0 |  | G/G | G/G | G/G |
| Control-0130 | 1 | 60 | 0 | 0 | 1 |  | G/G | G/G | G/G |
| Control-0131 | 1 | 57 | 0 | 0 | 1 |  | G/G | G/G | G/G |
| Control-0132 | 2 | 65 | 0 | 0 | 0 |  | G/G | G/G | G/G |
| Control-0133 | 2 | 70 | 0 | 0 | 0 |  | G/G | G/G | G/G |
| Control-0134 | 2 | 69 | 0 | 0 | 1 |  | G/A | G/A | G/A |
| Control-0135 | 1 | 53 | 1 | 0 | 0 |  | G/G | G/G | G/G |
| Control-0136 | 2 | 25 | 0 | 0 | 0 |  | G/G | G/G | G/G |
| Control-0137 | 2 | 43 | 0 | 0 | 0 |  | G/G | G/G | G/A |
| Control-0138 | 1 | 62 | 1 | 0 | 1 |  | G/G | G/G | G/G |
| Control-0139 | 2 | 74 | 0 | 0 | 1 |  | G/G | G/G | G/G |
| Control-0140 | 2 | 73 | 0 | 0 | 0 |  | G/G | G/G | G/G |
| Control-0141 | 2 | 62 | 0 | 0 | 0 |  | G/A | G/A | G/A |
| Control-0142 | 1 | 58 | 0 | 0 | 0 |  | G/A | A/A | G/A |
| Control-0143 | 2 | 69 | 0 | 0 | 1 |  | G/G | G/G | A/A |
| Control-0144 | 2 | 51 | 0 | 0 | 0 |  | G/G | G/G | G/A |
| Control-0145 | 1 | 61 | 0 | 0 | 1 |  | G/G | G/G | G/A |
| Control-0146 | 1 | 74 | 0 | 0 | 0 |  | G/A | G/G | G/G |
| Control-0147 | 1 | 55 | 0 | 0 | 1 |  | G/G | G/G | G/G |
| Control-0148 | 2 | 72 | 0 | 0 | 0 |  | G/A | G/A | G/A |
| Control-0149 | 1 | 56 | 0 | 0 | 0 |  | G/G | G/G | G/G |
| Control-0150 | 2 | 78 | 0 | 0 | 1 |  | G/A | G/G | G/G |
| Control-0151 | 2 | 63 | 0 | 0 | 1 |  | G/G | G/G | G/G |
| Control-0152 | 2 | 61 | 0 | 0 | 1 |  | G/G | G/G | G/G |
| Control-0153 | 1 | 59 | 1 | 0 | 1 |  | G/A | G/A | G/A |
| Control-0154 | 2 | 63 | 0 | 0 | 1 |  | A/A | G/G | G/G |
| Control-0155 | 2 | 39 | 0 | 0 | 1 |  | G/A | G/A | G/G |
| Control-0156 | 1 | 58 | 0 | 0 | 0 |  | G/G | G/G | G/G |
| Control-0157 | 1 | 54 | 0 | 0 | 0 |  | G/A | G/A | G/A |
| Control-0158 | 1 | 57 | 1 | 0 | 1 |  | G/G | G/G | G/G |
| Control-0159 | 1 | 66 | 0 | 0 | 0 |  | G/G | G/G | G/G |
| Control-0160 | 1 | 74 | 0 | 0 | 1 |  | G/G | G/G | G/G |
| Control-0161 | 2 | 50 | 0 | 0 | 0 |  | G/G | G/G | G/G |
| Control-0162 | 1 | 68 | 0 | 0 | 1 |  | G/A | G/G | G/A |
| Control-0163 | 1 | 60 | 0 | 0 | 1 |  | G/G | G/G | G/G |
| Control-0164 | 2 | 59 | 0 | 0 | 1 |  | G/G | G/G | G/G |
| Control-0165 | 1 | 73 | 0 | 0 | 1 |  | G/G | G/G | G/A |
| Control-0166 | 1 | 75 | 1 | 0 | 1 |  | G/G | G/G | G/G |
| Control-0167 | 1 | 65 | 0 | 0 | 0 |  | G/G | G/G | G/G |
| Control-0168 | 1 | 62 | 0 | 0 | 0 |  | G/A | G/A | G/A |
| Control-0169 | 1 | 57 | 0 | 0 | 0 |  | G/A | G/A | G/A |
| Control-0170 | 2 | 76 | 0 | 0 | 1 |  | G/G | G/G | G/G |
| Control-0171 | 2 | 60 | 0 | 0 | 1 |  | G/G | G/G | G/G |
| Control-0172 | 1 | 59 | 0 | 0 | 0 |  | G/A | G/A | G/G |
| Control-0173 | 2 | 52 | 0 | 0 | 0 |  | G/A | G/A | G/A |
| Control-0174 | 1 | 52 | 0 | 0 | 1 |  | G/G | G/G | G/G |
| Control-0175 | 1 | 76 | 0 | 0 | 1 |  | G/A | G/G | G/G |
| Control-0176 | 1 | 75 | 0 | 0 | 1 |  | G/A | G/G | G/G |
| Control-0177 | 2 | 51 | 0 | 0 | 1 |  | G/G | G/G | G/G |
| Control-0178 | 1 | 66 | 0 | 0 | 1 |  | G/G | G/G | G/G |
| Control-0179 | 2 | 61 | 0 | 0 | 0 |  | G/A | G/A | G/A |
| Control-0180 | 2 | 62 | 0 | 0 | 1 |  | G/G | G/G | G/G |
| Control-0181 | 1 | 40 | 0 | 0 | 1 |  | G/G | G/A | G/A |
| Control-0182 | 1 | 50 | 0 | 1 | 1 |  | G/G | G/A | G/G |
| Control-0183 | 2 | 59 | 0 | 0 | 0 |  | G/G | G/G | G/G |
| Control-0184 | 1 | 48 | 0 | 0 | 1 |  | G/A | G/A | G/A |
| Control-0185 | 1 | 74 | 0 | 1 | 1 |  | G/G | G/G | G/G |
| Control-0186 | 1 | 84 | 0 | 0 | 0 |  | G/G | G/G | G/A |
| Control-0187 | 1 | 57 | 0 | 0 | 0 |  | G/A | G/A | G/G |
| Control-0188 | 1 | 69 | 0 | 0 | 1 |  | G/G | G/G | G/A |
| Control-0189 | 1 | 62 | 0 | 0 | 1 |  | A/A | G/A | G/A |
| Control-0190 | 2 | 57 | 0 | 0 | 1 |  | G/G | G/G | G/G |
| Control-0191 | 1 | 37 | 0 | 0 | 0 |  | G/G | G/G | G/G |
| Control-0192 | 1 | 64 | 0 | 0 | 1 |  | G/A | G/A | G/G |
| Control-0193 | 1 | 64 | 0 | 0 | 0 |  | G/A | G/A | G/A |
| Control-0194 | 1 | 77 | 0 | 0 | 0 |  | G/A | G/A | G/A |
| Control-0195 | 1 | 60 | 0 | 0 | 0 |  | G/G | G/G | G/G |
| Control-0196 | 1 | 68 | 0 | 0 | 0 |  | G/A | G/G | G/G |
| Control-0197 | 1 | 81 | 0 | 0 | 1 |  | G/G | G/G | G/G |
| Control-0198 | 2 | 55 | 0 | 0 | 1 |  | G/G | G/G | G/G |
| Control-0199 | 1 | 79 | 1 | 1 | 0 |  | G/G | G/G | G/G |
| Control-0200 | 1 | 71 | 1 | 1 | 0 |  | G/G | G/G | G/G |
| Control-0201 | 1 | 64 | 0 | 0 | 0 |  | G/G | G/G | G/G |
| Control-0202 | 2 | 57 | 0 | 0 | 0 |  | G/G | G/G | G/G |
| Control-0203 | 2 | 78 | 0 | 0 | 1 |  | G/G | G/G | G/G |
| Control-0204 | 1 | 58 | 1 | 0 | 1 |  | G/G | G/G | G/G |
| Control-0205 | 1 | 74 | 0 | 0 | 0 |  | G/G | G/A | G/G |
| Control-0206 | 2 | 54 | 0 | 0 | 1 |  | G/G | G/G | G/G |
| Control-0207 | 1 | 59 | 0 | 1 | 0 |  | G/G | G/G | G/G |
| Control-0208 | 1 | 70 | 0 | 0 | 0 |  | G/A | G/G | A/A |
| Control-0209 | 1 | 55 | 0 | 0 | 1 |  | G/G | G/G | G/G |
| Control-0210 | 1 | 58 | 0 | 0 | 0 |  | G/G | G/G | G/G |
| Control-0211 | 1 | 59 | 0 | 0 | 1 |  | G/G | G/G | G/G |
| Control-0212 | 1 | 60 | 0 | 0 | 0 |  | G/G | G/G | G/G |
| Control-0213 | 1 | 58 | 0 | 0 | 0 |  | G/G | G/G | G/G |
| Control-0214 | 2 | 60 | 0 | 0 | 0 |  | G/G | G/G | G/G |
| Control-0215 | 2 | 61 | 0 | 0 | 0 |  | G/G | G/G | G/G |
| Control-0216 | 2 | 67 | 0 | 0 | 0 |  | G/G | G/G | G/G |
| Control-0217 | 1 | 59 | 1 | 0 | 1 |  | G/G | G/G | G/G |
| Control-0218 | 2 | 55 | 0 | 0 | 0 |  | G/G | G/G | G/G |
| Control-0219 | 1 | 66 | 0 | 0 | 0 |  | G/A | G/A | G/G |
| Control-0220 | 1 | 64 | 0 | 0 | 1 |  | G/G | G/G | G/G |
| Control-0221 | 1 | 56 | 1 | 0 | 1 |  | G/G | G/G | G/A |
| Control-0222 | 2 | 61 | 0 | 0 | 1 |  | A/A | G/A | A/A |
| Control-0223 | 1 | 60 | 1 | 0 | 0 |  | G/G | G/G | G/G |
| Control-0224 | 2 | 66 | 0 | 0 | 0 |  | G/G | G/G | G/A |
| Control-0225 | 2 | 63 | 0 | 0 | 1 |  | G/G | G/G | G/G |
| Control-0226 | 1 | 65 | 1 | 0 | 1 |  | G/A | G/G | G/A |
| Control-0227 | 1 | 60 | 0 | 0 | 0 |  | G/G | G/G | G/G |
| Control-0228 | 2 | 51 | 0 | 0 | 0 |  | G/G | G/G | G/G |
| Control-0229 | 1 | 57 | 0 | 0 | 1 |  | G/G | G/G | G/G |
| Control-0230 | 2 | 79 | 0 | 0 | 1 |  | G/G | G/G | G/G |
| Control-0231 | 1 | 55 | 1 | 1 | 0 |  | G/G | G/G | G/G |
| Control-0232 | 1 | 51 | 0 | 0 | 1 |  | G/G | G/G | G/G |
| Control-0233 | 2 | 66 | 0 | 0 | 0 |  | G/G | G/G | G/G |
| Control-0234 | 1 | 53 | 1 | 0 | 0 |  | G/G | G/G | G/G |
| Control-0235 | 1 | 61 | 0 | 0 | 1 |  | G/G | G/G | G/G |
| Control-0236 | 1 | 62 | 0 | 0 | 1 |  | G/G | G/G | G/G |
| Control-0237 | 1 | 60 | 1 | 0 | 1 |  | G/G | G/G | G/G |
| Control-0238 | 1 | 71 | 1 | 1 | 1 |  | G/G | G/G | G/A |
| Control-0239 | 1 | 67 | 0 | 0 | 0 |  | G/G | G/G | G/A |
| Control-0240 | 2 | 69 | 0 | 0 | 0 |  | G/G | G/G | G/G |
| Control-0241 | 1 | 62 | 0 | 0 | 0 |  | G/G | G/G | G/G |
| Control-0242 | 1 | 61 | 1 | 0 | 0 |  | G/G | G/G | G/G |
| Control-0243 | 1 | 62 | 1 | 0 | 0 |  | G/G | G/G | G/G |
| Control-0244 | 2 | 70 | 0 | 0 | 1 |  | G/G | G/G | G/G |
| Control-0245 | 2 | 56 | 0 | 0 | 1 |  | G/G | G/G | G/G |
| Control-0246 | 2 | 68 | 0 | 0 | 1 |  | G/G | G/G | G/A |
| Control-0247 | 2 | 53 | 0 | 0 | 1 |  | G/G | G/G | G/G |
| Control-0248 | 2 | 56 | 0 | 0 | 1 |  | G/G | G/G | G/A |
| Control-0249 | 2 | 48 | 0 | 0 | 0 |  | G/G | G/G | G/G |
| Control-0250 | 1 | 61 | 0 | 0 | 1 |  | G/G | G/G | G/G |
| Control-0251 | 1 | 67 | 0 | 0 | 1 |  | G/A | G/G | G/G |
| Control-0252 | 1 | 57 | 0 | 0 | 0 |  | G/G | G/G | G/G |
| Control-0253 | 1 | 63 | 0 | 0 | 0 |  | G/G | G/G | G/A |
| Control-0254 | 1 | 74 | 1 | 0 | 0 |  | G/G | G/G | G/G |
| Control-0255 | 1 | 67 | 0 | 0 | 0 |  | G/G | G/G | G/G |
| Control-0256 | 1 | 61 | 0 | 0 | 0 |  | G/G | G/G | G/A |
| Control-0257 | 1 | 74 | 0 | 1 | 0 |  | G/G | G/A | G/G |
| Control-0258 | 2 | 65 | 0 | 0 | 1 |  | G/A | G/A | G/A |
| Control-0259 | 1 | 71 | 1 | 0 | 0 |  | G/A | G/A | G/A |
| Control-0260 | 1 | 59 | 1 | 0 | 0 |  | G/G | G/G | G/G |
| Control-0261 | 1 | 69 | 1 | 0 | 0 |  | G/A | G/A | G/G |
| Control-0262 | 2 | 78 | 0 | 0 | 0 |  | G/G | G/G | G/G |
| Control-0263 | 1 | 59 | 0 | 0 | 1 |  | G/A | G/G | G/A |
| Control-0264 | 1 | 63 | 0 | 0 | 1 |  | G/A | G/A | G/A |
| Control-0265 | 2 | 63 | 0 | 0 | 1 |  | G/G | G/G | G/G |
| Control-0266 | 1 | 71 | 0 | 0 | 1 |  | G/G | G/G | G/G |
| Control-0267 | 1 | 64 | 0 | 1 | 0 |  | G/A | G/A | G/G |
| Control-0268 | 1 | 56 | 0 | 0 | 0 |  | G/G | G/G | G/G |
| Control-0269 | 2 | 72 | 0 | 0 | 1 |  | G/G | G/G | G/G |
| Control-0270 | 1 | 62 | 1 | 0 | 0 |  | G/G | G/G | G/G |
| Control-0271 | 2 | 51 | 0 | 0 | 0 |  | G/A | G/A | G/A |
| Control-0272 | 1 | 58 | 1 | 0 | 0 |  | G/G | G/G | G/G |
| Control-0273 | 1 | 60 | 0 | 0 | 0 |  | G/G | G/G | G/G |
| Control-0274 | 2 | 66 | 0 | 0 | 0 |  | G/G | G/G | G/A |
| Control-0275 | 2 | 70 | 0 | 0 | 0 |  | G/G | G/G | G/G |
| Control-0276 | 1 | 57 | 0 | 0 | 1 |  | G/A | G/A | G/A |
| Control-0277 | 2 | 61 | 0 | 0 | 0 |  | G/A | G/A | G/A |
| Control-0278 | 2 | 59 | 0 | 0 | 0 |  | G/G | G/G | G/A |
| Control-0279 | 2 | 54 | 0 | 0 | 0 |  | G/A | G/G | G/A |
| Control-0280 | 1 | 64 | 0 | 0 | 0 |  | G/A | G/A | G/G |
| Control-0281 | 2 | 48 | 0 | 0 | 1 |  | G/G | G/G | G/A |
| Control-0282 | 1 | 63 | 1 | 0 | 0 |  | G/A | G/A | G/A |
| Control-0283 | 2 | 69 | 0 | 0 | 0 |  | G/G | G/G | G/G |
| Control-0284 | 2 | 74 | 0 | 0 | 1 |  | G/G | G/G | G/A |
| Control-0285 | 1 | 59 | 0 | 0 | 1 |  | G/G | G/G | G/G |
| Control-0286 | 2 | 63 | 0 | 0 | 1 |  | G/A | G/A | G/A |
| Control-0287 | 1 | 73 | 0 | 0 | 1 |  | G/G | G/G | G/G |
| Control-0288 | 2 | 68 | 0 | 0 | 0 |  | G/G | G/G | G/G |
| Control-0289 | 1 | 65 | 0 | 0 | 0 |  | G/G | G/G | G/G |
| Control-0290 | 1 | 73 | 0 | 0 | 1 |  | G/A | G/A | G/G |
| Control-0291 | 2 | 59 | 0 | 0 | 0 |  | G/G | G/G | G/G |
| Control-0292 | 1 | 65 | 1 | 0 | 0 |  | G/G | G/G | G/G |
| Control-0293 | 1 | 66 | 0 | 0 | 0 |  | G/G | G/G | G/G |
| Control-0294 | 2 | 67 | 0 | 0 | 0 |  | G/A | G/A | G/A |
| Control-0295 | 2 | 55 | 0 | 0 | 0 |  | G/G | G/G | G/G |
| Control-0296 | 1 | 71 | 0 | 0 | 1 |  | G/G | G/G | G/G |
| Control-0297 | 2 | 67 | 0 | 0 | 0 |  | G/G | G/G | G/G |
| Control-0298 | 1 | 53 | 0 | 0 | 1 |  | G/G | G/G | G/G |
| Control-0299 | 2 | 67 | 0 | 0 | 1 |  | G/A | G/A | G/G |
| Control-0300 | 2 | 68 | 0 | 0 | 1 |  | G/G | G/G | G/G |
| Control-0301 | 1 | 55 | 1 | 0 | 0 |  | G/G | G/G | G/G |
| Control-0302 | 2 | 63 | 0 | 0 | 1 |  | G/G | G/G | G/G |
| Control-0303 | 2 | 72 | 0 | 0 | 0 |  | G/A | G/G | G/G |
| Control-0304 | 1 | 56 | 1 | 0 | 1 |  | G/A | G/G | G/G |
| Control-0305 | 1 | 56 | 0 | 0 | 1 |  | G/G | G/G | G/G |
| Control-0306 | 1 | 65 | 0 | 0 | 0 |  | G/A | G/G | G/G |
| Control-0307 | 1 | 59 | 0 | 0 | 1 |  | G/A | G/A | G/A |
| Control-0308 | 1 | 72 | 0 | 0 | 0 |  | G/G | G/G | G/G |
| Control-0309 | 1 | 59 | 1 | 0 | 1 |  | G/G | G/G | G/A |
| Control-0310 | 1 | 77 | 1 | 0 | 0 |  | G/A | G/A | G/G |
| Control-0311 | 1 | 60 | 0 | 0 | 0 |  | G/A | G/A | G/G |
| Control-0312 | 1 | 56 | 1 | 0 | 0 |  | G/A | G/G | G/G |
| Control-0313 | 1 | 62 | 0 | 0 | 0 |  | G/G | G/G | G/G |
| Control-0314 | 2 | 65 | 0 | 0 | 1 |  | G/G | G/G | G/A |
| Control-0315 | 1 | 49 | 1 | 0 | 0 |  | G/G | G/G | G/G |
| Control-0316 | 1 | 71 | 1 | 0 | 1 |  | G/G | G/G | G/G |
| Control-0317 | 1 | 52 | 0 | 0 | 0 |  | G/A | G/A | G/A |
| Control-0318 | 1 | 85 | 1 | 0 | 1 |  | G/A | G/G | G/G |
| Control-0319 | 1 | 47 | 0 | 0 | 0 |  | G/A | G/A | A/A |
| Control-0320 | 1 | 48 | 0 | 0 | 1 |  | A/A | G/A | G/A |
| Control-0321 | 2 | 46 | 0 | 0 | 0 |  | G/G | G/G | G/G |
| Control-0322 | 2 | 51 | 0 | 0 | 0 |  | G/A | G/A | G/A |
| Control-0323 | 1 | 67 | 1 | 0 | 1 |  | G/A | G/A | G/A |
| Control-0324 | 1 | 78 | 0 | 0 | 0 |  | G/A | G/G | G/A |
| Control-0325 | 1 | 48 | 1 | 0 | 0 |  | G/G | G/G | G/G |
| Control-0326 | 2 | 56 | 0 | 0 | 0 |  | G/G | G/G | G/G |
| Control-0327 | 2 | 51 | 0 | 0 | 1 |  | G/G | G/G | G/G |
| Control-0328 | 2 | 63 | 0 | 0 | 1 |  | G/G | G/G | G/G |
| Control-0329 | 2 | 54 | 0 | 0 | 1 |  | G/G | G/G | G/G |
| Control-0330 | 2 | 52 | 0 | 0 | 1 |  | G/G | G/G | G/G |
| Control-0331 | 1 | 52 | 0 | 0 | 1 |  | G/G | G/A | G/A |
| Control-0332 | 1 | 58 | 0 | 0 | 0 |  | G/G | G/G | G/G |
| Control-0333 | 1 | 51 | 0 | 0 | 0 |  | G/G | G/G | G/G |
| Control-0334 | 2 | 68 | 0 | 0 | 1 |  | G/A | G/A | G/G |
| Control-0335 | 1 | 52 | 0 | 0 | 0 |  | G/G | G/G | G/G |
| Control-0336 | 2 | 51 | 0 | 0 | 1 |  | G/A | G/A | A/A |
| Control-0337 | 2 | 66 | 0 | 0 | 1 |  | G/G | G/G | G/G |
| Control-0338 | 2 | 62 | 0 | 0 | 0 |  | G/A | G/A | G/G |
| Control-0339 | 1 | 66 | 0 | 0 | 0 |  | G/G | G/G | G/G |
| Control-0340 | 1 | 65 | 0 | 0 | 1 |  | G/G | G/G | G/G |
| Control-0341 | 2 | 60 | 0 | 0 | 0 |  | G/G | G/G | G/G |
| Control-0342 | 1 | 48 | 0 | 0 | 1 |  | G/A | G/A | G/G |
| Control-0343 | 2 | 53 | 0 | 0 | 1 |  | G/G | G/G | G/G |
| Control-0344 | 1 | 70 | 1 | 0 | 1 |  | G/G | G/G | G/G |
| Control-0345 | 2 | 60 | 0 | 0 | 1 |  | G/G | G/G | G/G |
| Control-0346 | 2 | 63 | 0 | 0 | 0 |  | G/G | G/G | G/G |
| Control-0347 | 2 | 61 | 0 | 0 | 1 |  | G/G | G/G | G/G |
| Control-0348 | 1 | 55 | 0 | 0 | 0 |  | G/G | G/G | G/G |
| Control-0349 | 1 | 69 | 0 | 0 | 1 |  | G/G | G/G | G/G |
| Control-0350 | 1 | 67 | 0 | 0 | 1 |  | G/G | G/G | G/A |
| Control-0351 | 1 | 62 | 0 | 0 | 0 |  | G/A | G/A | G/A |
| Control-0352 | 2 | 49 | 0 | 0 | 0 |  | G/G | G/G | G/A |
| Control-0353 | 1 | 55 | 0 | 0 | 1 |  | G/A | G/G | A/A |
| Control-0354 | 1 | 62 | 1 | 0 | 0 |  | G/G | G/G | G/G |
| Control-0355 | 1 | 48 | 1 | 0 | 1 |  | G/A | G/A | G/A |
| Control-0356 | 1 | 49 | 1 | 0 | 1 |  | G/G | G/G | G/G |
| Control-0357 | 1 | 61 | 0 | 0 | 1 |  | G/G | G/G | G/G |
| Control-0358 | 2 | 53 | 0 | 0 | 0 |  | G/A | G/A | G/A |
| Control-0359 | 1 | 52 | 0 | 0 | 0 |  | G/G | G/G | G/A |
| Control-0360 | 2 | 67 | 0 | 0 | 0 |  | G/G | G/G | G/G |
| Control-0361 | 1 | 30 | 0 | 0 | 0 |  | G/A | G/A | G/A |
| Control-0362 | 1 | 58 | 0 | 0 | 0 |  | G/G | G/G | G/G |
| Control-0363 | 2 | 61 | 0 | 0 | 1 |  | G/G | G/G | G/G |
| Control-0364 | 1 | 54 | 0 | 0 | 0 |  | G/G | G/G | G/G |
| Control-0365 | 2 | 42 | 0 | 0 | 0 |  | G/G | G/G | G/G |
| Control-0366 | 2 | 61 | 0 | 0 | 0 |  | G/G | G/G | G/G |
| Control-0367 | 1 | 57 | 0 | 0 | 0 |  | G/G | G/G | G/G |
| Control-0368 | 1 | 50 | 1 | 0 | 1 |  | G/G | G/G | G/G |
| Control-0369 | 2 | 66 | 0 | 0 | 0 |  | G/G | G/G | G/G |
| Control-0370 | 1 | 55 | 1 | 0 | 1 |  | G/G | G/G | G/G |
| Control-0371 | 2 | 52 | 0 | 0 | 1 |  | G/A | G/A | G/A |
| Control-0372 | 1 | 41 | 0 | 0 | 0 |  | G/G | G/G | G/G |
| Control-0373 | 2 | 63 | 0 | 0 | 1 |  | G/G | G/G | G/G |
| Control-0374 | 1 | 59 | 0 | 0 | 1 |  | G/G | G/G | G/G |
| Control-0375 | 1 | 74 | 1 | 1 | 0 |  | G/A | G/A | G/A |
| Control-0376 | 1 | 68 | 0 | 0 | 0 |  | G/G | G/G | G/G |
| Control-0377 | 1 | 63 | 1 | 1 | 1 |  | G/G | G/G | G/G |
| Control-0378 | 1 | 60 | 1 | 0 | 0 |  | G/A | G/A | G/A |
| Control-0379 | 1 | 59 | 0 | 0 | 1 |  | G/G | G/G | G/G |
| Control-0380 | 2 | 57 | 0 | 0 | 1 |  | G/G | G/G | G/G |
| Control-0381 | 1 | 60 | 1 | 0 | 1 |  | G/A | G/A | G/G |
| Control-0382 | 2 | 42 | 0 | 0 | 0 |  | G/G | G/G | G/G |
| Control-0383 | 1 | 54 | 1 | 0 | 0 |  | G/G | G/G | G/G |
| Control-0384 | 2 | 42 | 0 | 0 | 1 |  | G/A | G/A | G/A |
| Control-0385 | 1 | 51 | 0 | 0 | 0 |  | G/G | G/A | G/G |
| Control-0386 | 1 | 50 | 0 | 0 | 1 |  | G/A | G/A | G/A |
| Control-0387 | 1 | 58 | 0 | 0 | 1 |  | G/G | G/G | G/G |
| Control-0388 | 2 | 65 | 1 | 0 | 1 |  | G/A | G/A | G/A |
| Control-0389 | 1 | 51 | 0 | 0 | 0 |  | G/G | G/G | G/A |
| Control-0390 | 1 | 64 | 0 | 0 | 1 |  | G/A | G/G | G/A |
| Control-0391 | 1 | 27 | 0 | 0 | 0 |  | G/G | G/G | G/G |
| Control-0392 | 1 | 63 | 0 | 0 | 0 |  | G/G | G/G | G/A |
| Control-0393 | 1 | 53 | 0 | 0 | 0 |  | G/G | G/G | G/G |
| Control-0394 | 2 | 64 | 0 | 0 | 0 |  | G/A | G/A | G/A |
| Control-0395 | 1 | 55 | 1 | 0 | 0 |  | G/A | G/A | G/A |
| Control-0396 | 1 | 39 | 1 | 0 | 0 |  | G/G | G/G | G/G |
| Control-0397 | 1 | 69 | 0 | 0 | 1 |  | G/G | G/G | G/G |
| Control-0398 | 1 | 50 | 1 | 0 | 0 |  | G/A | G/A | G/A |
| Control-0399 | 1 | 76 | 0 | 0 | 0 |  | G/G | G/G | G/G |
| Control-0400 | 1 | 79 | 1 | 0 | 0 |  | G/G | G/G | G/G |
| Control-0401 | 1 | 52 | 0 | 0 | 0 |  | G/G | G/G | G/G |
| Control-0402 | 2 | 58 | 0 | 0 | 1 |  | G/A | G/A | G/A |
| Control-0403 | 1 | 43 | 0 | 0 | 0 |  | G/G | G/A | G/G |
| Control-0404 | 2 | 53 | 0 | 0 | 0 |  | G/G | G/G | G/G |
| Control-0405 | 1 | 78 | 0 | 0 | 0 |  | G/A | G/A | G/A |
| Control-0406 | 1 | 63 | 1 | 0 | 0 |  | G/G | G/G | G/G |
| Control-0407 | 1 | 57 | 1 | 0 | 1 |  | G/G | G/G | G/G |
| Control-0408 | 2 | 60 | 0 | 0 | 0 |  | G/A | G/A | G/G |
| Control-0409 | 1 | 67 | 0 | 0 | 1 |  | G/G | G/A | G/G |
| Control-0410 | 1 | 66 | 0 | 0 | 1 |  | G/A | G/A | G/A |
| Control-0411 | 1 | 62 | 0 | 0 | 1 |  | G/G | G/G | G/G |
| Control-0412 | 2 | 61 | 0 | 0 | 1 |  | G/G | G/G | G/G |
| Control-0413 | 1 | 61 | 0 | 0 | 1 |  | G/G | G/G | G/G |
| Control-0414 | 1 | 66 | 0 | 0 | 1 |  | G/G | G/G | G/G |
| Control-0415 | 1 | 52 | 0 | 0 | 1 |  | G/A | G/A | G/A |
| Control-0416 | 1 | 60 | 1 | 0 | 0 |  | G/G | G/G | G/G |
| Control-0417 | 1 | 72 | 1 | 1 | 0 |  | G/G | G/G | G/G |
| Control-0418 | 1 | 70 | 0 | 0 | 0 |  | G/G | G/G | G/G |
| Control-0419 | 1 | 68 | 0 | 0 | 0 |  | G/G | G/G | G/G |
| Control-0420 | 1 | 71 | 1 | 0 | 0 |  | G/A | G/A | G/A |
| Control-0421 | 2 | 69 | 0 | 0 | 1 |  | G/G | G/G | G/G |
| Control-0422 | 2 | 61 | 0 | 0 | 1 |  | G/A | G/G | G/A |
| Control-0423 | 1 | 69 | 0 | 0 | 1 |  | G/G | G/G | G/G |
| Control-0424 | 1 | 74 | 1 | 0 | 1 |  | G/G | G/G | G/G |
| Control-0425 | 2 | 52 | 0 | 0 | 1 |  | G/G | G/G | G/A |
| Control-0426 | 1 | 41 | 0 | 0 | 0 |  | G/G | G/G | G/G |
| Control-0427 | 1 | 52 | 0 | 0 | 0 |  | G/G | G/G | G/G |
| Control-0428 | 2 | 44 | 0 | 0 | 0 |  | G/G | G/G | G/A |
| Control-0429 | 2 | 50 | 0 | 0 | 0 |  | G/G | G/G | G/A |
| Control-0430 | 1 | 63 | 0 | 0 | 1 |  | G/A | G/A | G/A |
| Control-0431 | 1 | 59 | 1 | 0 | 1 |  | G/G | G/G | G/A |
| Control-0432 | 1 | 44 | 0 | 0 | 1 |  | G/G | G/G | G/G |
| Control-0433 | 2 | 66 | 0 | 0 | 0 |  | G/A | A/A | G/A |
| Control-0434 | 1 | 59 | 1 | 1 | 0 |  | G/G | G/G | G/G |
| Control-0435 | 1 | 61 | 1 | 0 | 1 |  | G/A | G/G | G/G |
| Control-0436 | 2 | 53 | 0 | 0 | 1 |  | G/G | G/G | G/G |
| Control-0437 | 1 | 66 | 1 | 0 | 1 |  | G/G | G/G | G/G |
| Control-0438 | 2 | 54 | 0 | 0 | 0 |  | G/A | G/A | A/A |
| Control-0439 | 2 | 51 | 0 | 0 | 1 |  | G/G | G/G | G/G |
| Control-0440 | 2 | 61 | 0 | 0 | 0 |  | G/G | G/G | G/A |
| Control-0441 | 1 | 56 | 0 | 0 | 1 |  | G/G | G/G | G/G |
| Control-0442 | 1 | 52 | 0 | 0 | 1 |  | G/A | G/A | G/G |
| Control-0443 | 2 | 49 | 0 | 0 | 1 |  | G/A | G/A | A/A |
| Control-0444 | 2 | 48 | 0 | 0 | 0 |  | G/G | G/G | G/G |
| Control-0445 | 2 | 45 | 0 | 0 | 1 |  | G/G | G/G | G/G |
| Control-0446 | 2 | 47 | 0 | 0 | 0 |  | G/G | G/G | G/G |
| Control-0447 | 2 | 65 | 0 | 0 | 1 |  | G/G | G/G | G/G |
| Control-0448 | 1 | 73 | 1 | 0 | 0 |  | G/A | G/A | A/A |
| Control-0449 | 2 | 59 | 0 | 0 | 0 |  | G/G | G/G | G/G |
| Control-0450 | 1 | 59 | 0 | 0 | 0 |  | G/G | G/G | G/A |
| Control-0451 | 1 | 67 | 0 | 0 | 0 |  | G/G | G/G | G/A |
| Control-0452 | 1 | 59 | 0 | 0 | 1 |  | A/A | G/A | A/A |
| Control-0453 | 1 | 59 | 0 | 0 | 1 |  | G/A | G/A | G/A |
| Control-0454 | 1 | 47 | 1 | 0 | 1 |  | G/A | G/A | G/A |
| Control-0455 | 2 | 68 | 0 | 0 | 0 |  | G/A | G/G | G/A |
| Control-0456 | 1 | 64 | 0 | 1 | 0 |  | A/A | G/A | G/A |
| Control-0457 | 2 | 50 | 0 | 0 | 1 |  | G/G | G/A | G/G |
| Control-0458 | 1 | 74 | 1 | 0 | 0 |  | G/A | G/G | G/A |
| Control-0459 | 2 | 50 | 0 | 0 | 0 |  | G/G | G/G | G/G |
| Control-0460 | 1 | 70 | 0 | 0 | 0 |  | G/G | G/G | G/A |
| Control-0461 | 2 | 63 | 0 | 0 | 0 |  | G/G | G/G | G/G |
| Control-0462 | 1 | 53 | 0 | 0 | 0 |  | G/G | G/G | G/G |
| Control-0463 | 2 | 59 | 0 | 0 | 0 |  | G/G | G/G | G/G |
| Control-0464 | 1 | 58 | 0 | 0 | 0 |  | G/G | G/G | G/G |
| Control-0465 | 1 | 68 | 0 | 0 | 0 |  | G/G | G/G | G/A |
| Control-0466 | 2 | 53 | 0 | 0 | 1 |  | G/G | G/G | G/G |
| Control-0467 | 2 | 65 | 0 | 0 | 1 |  | G/G | G/G | G/G |
| Control-0468 | 1 | 73 | 1 | 0 | 0 |  | G/G | G/G | G/G |
| Control-0469 | 2 | 56 | 0 | 0 | 1 |  | G/G | G/G | G/A |
| Control-0470 | 1 | 59 | 0 | 0 | 0 |  | G/G | G/G | G/G |
| Control-0471 | 2 | 51 | 0 | 0 | 1 |  | G/G | G/G | G/G |
| Control-0472 | 1 | 62 | 0 | 0 | 1 |  | G/G | G/G | G/G |
| Control-0473 | 1 | 55 | 0 | 0 | 0 |  | G/G | G/G | G/A |
| Control-0474 | 2 | 59 | 0 | 0 | 1 |  | G/G | G/G | G/G |
| Control-0475 | 1 | 62 | 0 | 0 | 1 |  | G/G | G/G | G/G |
| Control-0476 | 1 | 69 | 0 | 0 | 0 |  | G/G | G/G | G/G |
| Control-0477 | 1 | 29 | 0 | 0 | 1 |  | G/G | G/A | G/G |
| Control-0478 | 1 | 59 | 0 | 0 | 0 |  | G/G | G/G | A/A |
| Control-0479 | 1 | 52 | 1 | 0 | 0 |  | G/G | G/G | G/G |
| Control-0480 | 1 | 60 | 0 | 0 | 1 |  | G/G | G/G | G/G |
| Control-0481 | 2 | 61 | 0 | 0 | 0 |  | G/A | G/A | G/A |
| Control-0482 | 2 | 69 | 0 | 0 | 1 |  | G/G | G/G | G/G |
| Control-0483 | 1 | 59 | 0 | 0 | 1 |  | G/G | G/G | G/G |
| Control-0484 | 2 | 63 | 0 | 0 | 1 |  | G/A | G/A | G/G |
| Control-0485 | 2 | 66 | 0 | 0 | 0 |  | G/G | G/G | G/G |
| Control-0486 | 1 | 63 | 0 | 0 | 1 |  | G/G | G/G | G/G |
| Control-0487 | 1 | 74 | 0 | 0 | 1 |  | G/G | G/G | G/G |
| Control-0488 | 2 | 59 | 0 | 0 | 1 |  | G/G | G/G | G/G |
| Control-0489 | 1 | 57 | 0 | 0 | 1 |  | G/A | G/A | A/A |
| Control-0490 | 1 | 63 | 0 | 0 | 0 |  | G/G | G/G | G/G |
| Control-0491 | 2 | 68 | 0 | 0 | 1 |  | G/G | G/G | G/G |
| Control-0492 | 1 | 61 | 1 | 0 | 1 |  | G/G | G/G | G/A |
| Control-0493 | 1 | 53 | 0 | 0 | 1 |  | G/G | G/G | G/G |
| Control-0494 | 2 | 58 | 0 | 0 | 0 |  | G/G | G/G | G/G |
| Control-0495 | 1 | 62 | 0 | 0 | 1 |  | G/G | G/G | G/G |
| Control-0496 | 1 | 57 | 1 | 0 | 0 |  | G/G | G/G | G/G |
| Control-0497 | 1 | 57 | 1 | 0 | 1 |  | G/A | G/G | G/G |
| Control-0498 | 1 | 46 | 1 | 1 | 1 |  | G/G | G/G | G/G |
| Control-0499 | 1 | 60 | 1 | 1 | 0 |  | G/G | G/G | G/G |
| Control-0500 | 2 | 65 | 0 | 0 | 0 |  | G/A | G/A | G/G |
| Control-0501 | 1 | 62 | 0 | 0 | 0 |  | G/G | G/G | G/A |
| Control-0502 | 1 | 54 | 1 | 0 | 0 |  | G/G | G/G | G/G |
| Control-0503 | 1 | 53 | 0 | 0 | 0 |  | G/A | G/A | G/G |
| Control-0504 | 2 | 64 | 0 | 0 | 1 |  | G/G | G/A | G/A |
| Control-0505 | 1 | 64 | 1 | 1 | 1 |  | G/G | G/G | G/G |
| Control-0506 | 1 | 55 | 0 | 0 | 1 |  | G/G | G/G | G/G |
| Control-0507 | 2 | 60 | 0 | 0 | 0 |  | G/G | G/G | G/A |
| Control-0508 | 1 | 59 | 1 | 0 | 0 |  | G/G | G/G | G/G |
| Control-0509 | 1 | 59 | 1 | 0 | 1 |  | G/G | G/A | G/G |
| Control-0510 | 1 | 63 | 1 | 0 | 0 |  | G/A | G/G | G/A |
| Control-0511 | 1 | 62 | 0 | 1 | 1 |  | G/G | G/G | G/G |
| Control-0512 | 1 | 53 | 0 | 0 | 1 |  | G/G | G/G | G/G |
| Control-0513 | 1 | 52 | 0 | 0 | 0 |  | G/G | G/G | G/A |
| Control-0514 | 1 | 54 | 1 | 0 | 1 |  | G/G | G/G | G/G |
| Control-0515 | 1 | 55 | 0 | 0 | 0 |  | G/G | G/G | G/G |
| Control-0516 | 2 | 69 | 0 | 0 | 1 |  | G/G | G/G | G/A |
| Control-0517 | 1 | 65 | 0 | 1 | 0 |  | G/A | G/G | G/G |
| Control-0518 | 1 | 58 | 0 | 1 | 1 |  | G/A | G/G | G/G |
| Control-0519 | 1 | 51 | 1 | 0 | 1 |  | G/G | G/G | G/G |
| Control-0520 | 1 | 48 | 1 | 0 | 1 |  | G/A | G/G | G/G |
| Control-0521 | 2 | 54 | 0 | 0 | 0 |  | G/G | G/G | G/G |
| Control-0522 | 2 | 60 | 0 | 0 | 1 |  | G/A | G/A | G/A |
| Control-0523 | 2 | 64 | 0 | 0 | 0 |  | G/G | G/G | G/G |
| Control-0524 | 1 | 59 | 1 | 1 | 1 |  | G/A | G/A | G/A |
| Control-0525 | 2 | 59 | 0 | 0 | 0 |  | G/A | G/A | G/A |
| Control-0526 | 1 | 56 | 1 | 0 | 0 |  | G/G | G/G | G/G |
| Control-0527 | 1 | 60 | 0 | 0 | 0 |  | G/G | G/A | G/G |
| Control-0528 | 1 | 51 | 0 | 0 | 0 |  | G/G | G/G | G/A |
| Control-0529 | 1 | 58 | 1 | 0 | 0 |  | G/G | G/G | G/G |
| Control-0530 | 1 | 65 | 0 | 0 | 0 |  | G/G | G/G | G/A |
| Control-0531 | 1 | 63 | 0 | 0 | 0 |  | G/A | G/G | G/G |
| Control-0532 | 2 | 58 | 0 | 0 | 0 |  | G/A | G/G | G/A |
| Control-0533 | 2 | 61 | 0 | 0 | 0 |  | G/G | G/G | G/G |
| Control-0534 | 1 | 62 | 1 | 1 | 1 |  | A/A | G/A | G/A |
| Control-0535 | 1 | 54 | 0 | 0 | 0 |  | G/G | G/G | G/G |
| Control-0536 | 1 | 58 | 0 | 0 | 1 |  | G/A | G/G | G/G |
| Control-0537 | 2 | 60 | 0 | 0 | 1 |  | G/G | G/G | G/G |
| Control-0538 | 1 | 59 | 1 | 0 | 1 |  | G/G | G/G | G/G |
| Control-0539 | 2 | 59 | 0 | 0 | 1 |  | G/G | G/G | G/G |
| Control-0540 | 1 | 64 | 0 | 0 | 1 |  | G/G | G/G | G/G |
| Control-0541 | 1 | 58 | 1 | 0 | 0 |  | G/G | G/G | G/A |
| Control-0542 | 1 | 60 | 0 | 0 | 1 |  | G/G | G/G | G/G |
| Control-0543 | 1 | 60 | 0 | 1 | 1 |  | G/A | G/A | G/A |
| Control-0544 | 2 | 50 | 0 | 0 | 0 |  | G/G | G/A | G/G |
| Control-0545 | 1 | 54 | 1 | 1 | 1 |  | G/A | G/A | G/G |
| Control-0546 | 1 | 58 | 0 | 0 | 1 |  | G/A | G/A | G/A |
| Control-0547 | 1 | 57 | 0 | 0 | 0 |  | G/A | G/G | G/G |
| Control-0548 | 1 | 62 | 0 | 0 | 1 |  | G/G | G/G | G/A |
| Control-0549 | 1 | 65 | 0 | 0 | 1 |  | G/G | G/G | G/G |
| Control-0550 | 1 | 61 | 0 | 0 | 1 |  | G/G | G/G | G/G |
| Control-0551 | 2 | 61 | 0 | 0 | 0 |  | G/G | G/G | G/G |
| Control-0552 | 2 | 57 | 0 | 0 | 0 |  | G/G | G/G | G/G |
| Control-0553 | 1 | 56 | 1 | 1 | 1 |  | G/A | G/A | G/A |
| Control-0554 | 2 | 59 | 0 | 0 | 1 |  | G/G | G/G | G/G |
| Control-0555 | 2 | 61 | 0 | 0 | 1 |  | G/G | G/G | G/G |
| Control-0556 | 2 | 54 | 0 | 1 | 0 |  | G/G | G/G | G/G |
| Control-0557 | 1 | 65 | 0 | 0 | 1 |  | G/G | G/G | G/G |
| Control-0558 | 1 | 64 | 1 | 0 | 0 |  | G/A | G/G | G/A |
| Control-0559 | 2 | 66 | 0 | 0 | 1 |  | G/A | G/G | G/G |
| Control-0560 | 1 | 56 | 1 | 1 | 0 |  | G/G | G/G | G/G |
| Control-0561 | 1 | 51 | 1 | 0 | 1 |  | G/A | G/G | G/A |
| Control-0562 | 1 | 54 | 0 | 0 | 1 |  | G/A | G/A | G/A |
| Control-0563 | 1 | 59 | 1 | 1 | 0 |  | G/A | G/A | G/A |
| Control-0564 | 1 | 57 | 1 | 0 | 1 |  | G/G | G/G | G/G |
| Control-0565 | 1 | 52 | 1 | 1 | 0 |  | G/G | G/G | G/G |
| Control-0566 | 2 | 65 | 0 | 0 | 0 |  | G/G | G/G | G/G |
| Control-0567 | 1 | 59 | 1 | 0 | 0 |  | G/G | G/G | G/G |
| Control-0568 | 1 | 64 | 0 | 1 | 1 |  | G/G | G/G | G/G |
| Control-0569 | 1 | 57 | 1 | 0 | 1 |  | G/A | G/A | G/A |
| Control-0570 | 1 | 52 | 0 | 0 | 1 |  | G/G | G/G | G/G |
| Control-0571 | 1 | 51 | 0 | 0 | 1 |  | G/A | G/A | G/A |
| Control-0572 | 1 | 63 | 0 | 0 | 0 |  | G/G | G/G | G/G |
| Control-0573 | 1 | 63 | 1 | 0 | 1 |  | G/G | G/G | G/A |
| Control-0574 | 2 | 68 | 0 | 0 | 1 |  | G/G | G/G | G/G |
| Control-0575 | 2 | 55 | 0 | 0 | 1 |  | G/G | G/G | G/G |
| Control-0576 | 1 | 55 | 1 | 1 | 1 |  | G/G | G/G | G/G |
| Control-0577 | 1 | 64 | 1 | 1 | 0 |  | G/G | G/G | G/G |
| Control-0578 | 1 | 60 | 0 | 0 | 0 |  | G/G | G/G | G/G |
| Control-0579 | 1 | 50 | 0 | 0 | 0 |  | G/G | G/G | G/G |
| Control-0580 | 1 | 51 | 0 | 1 | 1 |  | G/A | G/G | G/A |
| Control-0581 | 1 | 58 | 1 | 1 | 0 |  | G/G | G/A | G/G |
| Control-0582 | 1 | 57 | 0 | 0 | 0 |  | G/G | G/G | G/G |
| Control-0583 | 1 | 61 | 1 | 1 | 1 |  | G/G | G/G | G/A |
| Control-0584 | 1 | 63 | 1 | 0 | 0 |  | G/A | G/A | G/A |
| Control-0585 | 1 | 60 | 1 | 0 | 0 |  | G/G | G/G | G/G |
| Control-0586 | 2 | 66 | 0 | 0 | 1 |  | G/A | G/A | G/A |
| Control-0587 | 1 | 65 | 1 | 0 | 0 |  | G/G | G/G | G/G |
| Control-0588 | 2 | 63 | 0 | 0 | 0 |  | G/G | G/G | G/G |
| Control-0589 | 2 | 59 | 0 | 0 | 1 |  | G/G | G/G | G/G |
| Control-0590 | 1 | 59 | 1 | 1 | 0 |  | G/G | G/G | G/A |
| Control-0591 | 1 | 61 | 1 | 1 | 0 |  | G/A | G/A | G/G |
| Control-0592 | 1 | 50 | 0 | 1 | 1 |  | G/A | G/A | G/A |
| Control-0593 | 2 | 50 | 0 | 0 | 1 |  | A/A | A/A | G/A |
| Control-0594 | 2 | 56 | 0 | 0 | 0 |  | G/A | G/A | G/A |
| Control-0595 | 1 | 55 | 0 | 0 | 0 |  | G/A | G/G | G/A |
| Control-0596 | 2 | 59 | 0 | 0 | 1 |  | G/G | G/G | G/G |
| Control-0597 | 1 | 59 | 0 | 0 | 0 |  | G/G | G/G | G/G |
| Control-0598 | 1 | 59 | 1 | 0 | 0 |  | G/G | G/G | G/G |
| Control-0599 | 1 | 56 | 0 | 0 | 1 |  | G/G | G/G | G/G |
| Control-0600 | 2 | 63 | 0 | 0 | 1 |  | G/G | G/G | G/G |
| Control-0601 | 1 | 52 | 1 | 0 | 1 |  | G/A | G/A | G/A |
| Control-0602 | 2 | 68 | 0 | 0 | 1 |  | G/G | G/G | G/G |
| Control-0603 | 2 | 53 | 0 | 0 | 1 |  | G/G | G/G | G/G |
| Control-0604 | 1 | 61 | 1 | 1 | 0 |  | G/G | G/G | G/G |
| Control-0605 | 2 | 68 | 0 | 0 | 0 |  | G/G | G/G | G/G |
| Control-0606 | 1 | 56 | 0 | 0 | 0 |  | G/G | G/G | G/A |
| Control-0607 | 1 | 64 | 1 | 1 | 0 |  | G/G | G/G | G/G |
| Control-0608 | 2 | 53 | 0 | 0 | 1 |  | G/G | G/G | G/G |
| Control-0609 | 1 | 52 | 0 | 0 | 1 |  | G/G | G/G | G/G |
| Control-0610 | 1 | 52 | 1 | 0 | 1 |  | G/G | G/G | G/G |
| Control-0611 | 1 | 66 | 0 | 0 | 0 |  | G/G | G/G | G/A |
| Control-0612 | 2 | 57 | 0 | 0 | 0 |  | G/G | G/G | G/G |
| Control-0613 | 1 | 56 | 1 | 1 | 0 |  | G/G | G/G | G/G |
| Control-0614 | 2 | 62 | 0 | 0 | 0 |  | G/G | G/G | G/G |
| Control-0615 | 2 | 61 | 0 | 0 | 0 |  | G/G | G/G | G/G |
| Control-0616 | 2 | 57 | 0 | 0 | 0 |  | G/A | G/G | G/G |
| Control-0617 | 2 | 63 | 0 | 0 | 1 |  | G/G | G/G | G/G |
| Control-0618 | 2 | 58 | 0 | 0 | 1 |  | G/A | G/A | G/G |
| Control-0619 | 1 | 50 | 0 | 0 | 0 |  | G/G | G/G | G/G |
| Control-0620 | 2 | 55 | 0 | 0 | 1 |  | G/G | G/G | G/G |
| Control-0621 | 1 | 56 | 0 | 0 | 1 |  | G/G | G/G | G/G |
| Control-0622 | 2 | 59 | 0 | 0 | 0 |  | G/A | G/G | G/A |
| Control-0623 | 1 | 65 | 1 | 1 | 0 |  | G/G | G/G | G/G |
| Control-0624 | 2 | 47 | 0 | 0 | 1 |  | G/G | G/G | G/G |
| Control-0625 | 1 | 58 | 1 | 1 | 0 |  | G/G | G/G | G/A |
| Control-0626 | 1 | 60 | 1 | 1 | 0 |  | G/G | G/G | G/G |
| Control-0627 | 1 | 69 | 0 | 0 | 1 |  | G/G | G/G | G/G |
| Control-0628 | 2 | 62 | 0 | 0 | 0 |  | G/G | G/G | G/G |
| Control-0629 | 2 | 61 | 0 | 0 | 1 |  | G/G | G/G | G/G |
| Control-0630 | 2 | 69 | 0 | 0 | 1 |  | G/G | G/G | G/G |
| Control-0631 | 2 | 53 | 0 | 0 | 0 |  | G/G | G/G | G/G |
| Control-0632 | 1 | 58 | 0 | 0 | 1 |  | G/G | G/G | G/G |
| Control-0633 | 2 | 61 | 0 | 0 | 0 |  | G/G | G/G | G/G |
| Control-0634 | 1 | 61 | 0 | 0 | 0 |  | A/A | A/A | A/A |
| Control-0635 | 2 | 53 | 0 | 0 | 0 |  | G/G | G/G | G/A |
| Control-0636 | 1 | 69 | 0 | 0 | 1 |  | G/A | G/G | G/G |
| Control-0637 | 2 | 45 | 0 | 0 | 1 |  | G/G | G/G | G/A |
| Control-0638 | 1 | 55 | 1 | 1 | 0 |  | G/G | G/G | G/G |
| Control-0639 | 2 | 52 | 0 | 0 | 1 |  | G/G | G/G | G/G |
| Control-0640 | 1 | 67 | 0 | 0 | 1 |  | G/G | G/G | G/A |
| Control-0641 | 1 | 53 | 1 | 0 | 1 |  | G/G | G/G | G/G |
| Control-0642 | 2 | 59 | 0 | 0 | 1 |  | G/A | G/A | G/G |
| Control-0643 | 1 | 49 | 1 | 1 | 0 |  | G/A | G/A | G/A |
| Control-0644 | 2 | 59 | 0 | 0 | 1 |  | G/G | G/G | G/G |
| Control-0645 | 1 | 55 | 1 | 0 | 1 |  | G/A | G/A | G/G |
| Control-0646 | 1 | 62 | 0 | 0 | 1 |  | G/A | G/G | G/G |
| Control-0647 | 2 | 63 | 0 | 0 | 1 |  | G/G | G/G | G/A |
| Control-0648 | 1 | 57 | 0 | 1 | 0 |  | G/G | G/G | G/G |
| Control-0649 | 1 | 53 | 0 | 0 | 0 |  | G/A | G/A | G/A |
| Control-0650 | 2 | 68 | 0 | 0 | 0 |  | G/G | G/G | G/G |
| Control-0651 | 1 | 45 | 0 | 0 | 1 |  | G/G | G/G | G/G |
| Control-0652 | 1 | 67 | 1 | 1 | 0 |  | G/G | G/G | G/G |
| Control-0653 | 1 | 52 | 0 | 0 | 1 |  | G/G | G/G | G/G |
| Control-0654 | 2 | 47 | 0 | 0 | 0 |  | G/G | G/G | G/G |
| Control-0655 | 1 | 53 | 0 | 0 | 1 |  | G/A | G/A | G/A |
| Control-0656 | 2 | 56 | 0 | 0 | 0 |  | G/A | G/G | G/G |
| Control-0657 | 2 | 53 | 0 | 0 | 0 |  | G/G | G/G | G/G |
| Control-0658 | 1 | 52 | 0 | 1 | 0 |  | G/G | G/G | G/A |
| Control-0659 | 2 | 67 | 0 | 0 | 1 |  | G/A | A/A | G/A |
| Control-0660 | 2 | 48 | 0 | 0 | 1 |  | G/A | G/G | G/A |
| Control-0661 | 1 | 52 | 0 | 0 | 1 |  | G/A | G/A | G/A |
| Control-0662 | 2 | 65 | 0 | 0 | 1 |  | G/A | G/A | G/G |
| Control-0663 | 2 | 48 | 0 | 0 | 0 |  | G/G | G/G | G/G |
| Control-0664 | 2 | 59 | 0 | 0 | 0 |  | G/A | G/G | G/A |
| Control-0665 | 1 | 69 | 1 | 1 | 0 |  | G/A | G/A | G/A |
| Control-0666 | 1 | 50 | 0 | 0 | 0 |  | G/G | G/G | G/G |
| Control-0667 | 2 | 53 | 0 | 0 | 1 |  | G/G | G/G | G/G |
| Control-0668 | 1 | 54 | 0 | 0 | 1 |  | G/G | G/G | G/G |
| Control-0669 | 1 | 56 | 0 | 0 | 1 |  | G/G | G/G | G/A |
| Control-0670 | 2 | 58 | 0 | 0 | 1 |  | G/A | G/A | G/G |
| Control-0671 | 1 | 60 | 0 | 0 | 0 |  | G/G | G/G | G/G |
| Control-0672 | 1 | 82 | 1 | 0 | 0 |  | G/A | G/A | G/G |
| Control-0673 | 1 | 60 | 1 | 0 | 0 |  | G/A | G/G | G/G |
| Control-0674 | 2 | 57 | 0 | 0 | 1 |  | G/G | G/G | G/G |
| Control-0675 | 1 | 55 | 0 | 0 | 0 |  | G/G | G/G | G/G |
| Control-0676 | 2 | 68 | 0 | 0 | 0 |  | G/G | G/G | G/G |
| Control-0677 | 2 | 61 | 0 | 0 | 1 |  | G/G | G/G | G/G |
| Control-0678 | 1 | 45 | 1 | 1 | 0 |  | G/G | G/G | G/G |
| Control-0679 | 2 | 64 | 0 | 0 | 0 |  | G/G | G/G | G/G |
| Control-0680 | 2 | 66 | 0 | 0 | 0 |  | G/G | G/G | G/G |
| Control-0681 | 2 | 67 | 0 | 0 | 0 |  | G/A | G/G | G/G |
| Control-0682 | 2 | 51 | 0 | 0 | 1 |  | G/G | G/G | G/G |
| Control-0683 | 2 | 67 | 0 | 0 | 1 |  | A/A | G/A | G/G |
| Control-0684 | 1 | 51 | 1 | 0 | 0 |  | G/G | G/G | G/A |
| Control-0685 | 1 | 65 | 0 | 0 | 0 |  | G/G | G/G | G/G |
| Control-0686 | 2 | 61 | 0 | 0 | 1 |  | G/A | G/G | G/G |
| Control-0687 | 2 | 65 | 0 | 0 | 0 |  | G/G | G/G | G/A |
| Control-0688 | 1 | 65 | 0 | 0 | 1 |  | G/A | G/G | G/A |
| Control-0689 | 1 | 60 | 1 | 0 | 1 |  | G/G | G/G | G/G |
| Control-0690 | 1 | 58 | 1 | 1 | 1 |  | G/G | G/G | G/G |
| Control-0691 | 2 | 67 | 0 | 0 | 0 |  | G/G | G/G | G/G |
| Control-0692 | 2 | 66 | 0 | 0 | 1 |  | G/A | G/G | G/A |
| Control-0693 | 1 | 68 | 0 | 0 | 1 |  | G/G | G/G | G/G |
| Control-0694 | 1 | 43 | 0 | 0 | 1 |  | G/G | G/G | G/G |
| Control-0695 | 2 | 47 | 0 | 0 | 1 |  | G/G | G/G | G/G |
| Control-0696 | 2 | 52 | 0 | 0 | 1 |  | G/G | G/G | G/A |
| Control-0697 | 1 | 52 | 0 | 0 | 1 |  | G/G | G/G | G/G |
| Control-0698 | 2 | 61 | 0 | 0 | 0 |  | G/G | G/G | G/G |
| Control-0699 | 2 | 66 | 0 | 0 | 0 |  | G/G | G/G | G/G |
| Control-0700 | 2 | 59 | 0 | 0 | 0 |  | G/A | G/A | G/A |
| Control-0701 | 1 | 63 | 1 | 0 | 1 |  | G/G | G/G | G/G |
| Control-0702 | 1 | 59 | 1 | 1 | 0 |  | G/G | G/G | G/A |
| Control-0703 | 2 | 56 | 0 | 0 | 1 |  | G/G | G/G | G/G |
| Control-0704 | 2 | 69 | 0 | 0 | 1 |  | G/G | G/G | G/G |
| Control-0705 | 1 | 58 | 1 | 1 | 1 |  | G/A | G/A | G/A |
| Control-0706 | 1 | 49 | 1 | 1 | 1 |  | G/G | G/G | G/G |
| Control-0707 | 2 | 60 | 0 | 0 | 1 |  | G/G | G/G | G/G |
| Control-0708 | 1 | 51 | 1 | 1 | 0 |  | G/G | G/G | G/G |
| Control-0709 | 2 | 51 | 0 | 0 | 1 |  | G/G | G/G | G/G |
| Control-0710 | 1 | 46 | 1 | 0 | 1 |  | G/G | G/G | G/G |
| Control-0711 | 1 | 64 | 1 | 0 | 0 |  | G/G | G/G | G/G |
| Control-0712 | 1 | 56 | 1 | 1 | 1 |  | G/A | G/A | G/A |
| Control-0713 | 2 | 46 | 0 | 0 | 0 |  | G/A | G/A | G/A |
| Control-0714 | 2 | 56 | 0 | 0 | 0 |  | G/G | G/G | G/G |
| Control-0715 | 1 | 53 | 0 | 0 | 1 |  | G/A | G/A | G/A |
| Control-0716 | 2 | 66 | 0 | 0 | 1 |  | G/A | G/G | G/G |
| Control-0717 | 2 | 54 | 0 | 0 | 1 |  | G/G | G/G | G/A |
| Control-0718 | 1 | 53 | 0 | 0 | 0 |  | G/G | G/G | G/A |
| Control-0719 | 1 | 65 | 1 | 0 | 1 |  | G/A | G/G | G/G |
| Control-0720 | 2 | 60 | 0 | 0 | 0 |  | G/G | G/G | G/G |
| Control-0721 | 2 | 48 | 0 | 0 | 0 |  | G/G | G/G | G/G |
| Control-0722 | 1 | 52 | 0 | 0 | 0 |  | G/G | G/A | G/G |
| Control-0723 | 2 | 62 | 0 | 0 | 0 |  | G/G | G/G | G/G |
| Control-0724 | 2 | 61 | 0 | 0 | 0 |  | G/G | G/G | G/G |
| Control-0725 | 1 | 56 | 0 | 0 | 1 |  | G/G | G/G | G/G |
| Control-0726 | 1 | 57 | 0 | 0 | 1 |  | G/A | G/A | G/A |
| Control-0727 | 1 | 61 | 1 | 1 | 0 |  | G/G | G/G | G/G |
| Control-0728 | 2 | 65 | 0 | 0 | 0 |  | G/G | G/G | G/G |
| Control-0729 | 2 | 68 | 0 | 0 | 0 |  | G/G | G/A | G/A |
| Control-0730 | 1 | 60 | 1 | 0 | 0 |  | G/G | G/G | G/G |
| Control-0731 | 1 | 65 | 0 | 0 | 1 |  | G/G | G/G | G/A |
| Control-0732 | 2 | 56 | 0 | 0 | 0 |  | G/A | G/G | G/A |
| Control-0733 | 1 | 50 | 0 | 1 | 0 |  | G/G | G/G | G/G |
| Control-0734 | 1 | 49 | 0 | 0 | 0 |  | G/G | G/G | G/G |
| Control-0735 | 2 | 47 | 0 | 0 | 1 |  | G/G | G/A | G/A |
| Control-0736 | 2 | 71 | 0 | 0 | 1 |  | G/A | G/G | G/G |
| Control-0737 | 2 | 64 | 0 | 0 | 0 |  | G/A | G/G | G/G |
| Control-0738 | 2 | 61 | 0 | 0 | 1 |  | G/G | G/G | G/G |
| Control-0739 | 2 | 60 | 0 | 0 | 0 |  | G/G | G/G | G/G |
| Control-0740 | 2 | 66 | 0 | 0 | 1 |  | G/G | G/G | G/G |
| Control-0741 | 2 | 77 | 0 | 0 | 0 |  | G/G | G/G | G/G |
| Control-0742 | 2 | 75 | 0 | 0 | 1 |  | G/G | G/G | G/G |
| Control-0743 | 2 | 43 | 0 | 0 | 1 |  | G/A | G/A | G/A |
| Control-0744 | 2 | 66 | 0 | 0 | 1 |  | G/G | G/G | G/G |
| Control-0745 | 1 | 58 | 0 | 0 | 0 |  | G/G | G/G | G/G |
| Control-0746 | 1 | 58 | 0 | 1 | 1 |  | G/G | G/G | G/G |
| Control-0747 | 1 | 62 | 0 | 0 | 1 |  | G/G | G/G | G/G |
| Control-0748 | 2 | 66 | 0 | 0 | 1 |  | G/G | G/G | G/G |
| Control-0749 | 2 | 61 | 0 | 0 | 0 |  | G/G | G/G | G/G |
| Control-0750 | 1 | 66 | 1 | 0 | 0 |  | G/G | G/G | G/G |
| Control-0751 | 2 | 61 | 0 | 0 | 0 |  | G/G | G/G | G/G |
| Control-0752 | 2 | 70 | 0 | 0 | 1 |  | G/A | G/A | G/G |
| Control-0753 | 1 | 70 | 0 | 0 | 1 |  | G/G | G/G | G/G |
| Control-0754 | 1 | 69 | 1 | 1 | 1 |  | A/A | G/A | G/G |
| Control-0755 | 1 | 60 | 0 | 0 | 0 |  | G/G | G/G | G/G |
| Control-0756 | 2 | 72 | 0 | 0 | 1 |  | G/G | G/G | G/G |
| Control-0757 | 1 | 56 | 0 | 0 | 1 |  | G/G | G/G | G/G |
| Control-0758 | 1 | 66 | 0 | 0 | 0 |  | G/G | G/G | G/G |
| Control-0759 | 2 | 51 | 0 | 0 | 0 |  | G/A | A/A | G/A |
| Control-0760 | 1 | 47 | 1 | 1 | 1 |  | G/G | G/G | G/A |
| Control-0761 | 2 | 72 | 0 | 0 | 0 |  | G/G | G/G | G/G |
| Control-0762 | 2 | 74 | 0 | 0 | 0 |  | G/G | G/G | G/A |
| Control-0763 | 2 | 59 | 0 | 0 | 0 |  | G/A | G/A | G/G |
| Control-0764 | 1 | 69 | 0 | 0 | 1 |  | G/G | G/G | G/G |
| Control-0765 | 1 | 64 | 0 | 0 | 0 |  | G/G | G/G | G/G |
| Control-0766 | 2 | 66 | 0 | 0 | 0 |  | G/A | G/G | G/A |
| Control-0767 | 2 | 72 | 0 | 0 | 1 |  | G/G | G/G | G/G |
| Control-0768 | 2 | 67 | 0 | 0 | 1 |  | G/A | G/G | G/G |
| Control-0769 | 2 | 79 | 0 | 0 | 1 |  | G/A | G/A | G/A |
| Control-0770 | 2 | 59 | 0 | 0 | 0 |  | G/A | G/G | G/A |
| Control-0771 | 2 | 53 | 0 | 0 | 0 |  | A/A | G/A | A/A |
| Control-0772 | 1 | 66 | 0 | 0 | 1 |  | G/G | G/G | G/A |
| Control-0773 | 1 | 50 | 0 | 0 | 1 |  | G/G | G/G | G/G |
| Control-0774 | 1 | 67 | 0 | 0 | 1 |  | G/G | G/G | G/G |
| Control-0775 | 1 | 83 | 0 | 0 | 1 |  | G/G | G/G | G/G |
| Control-0776 | 2 | 50 | 0 | 0 | 0 |  | G/G | G/G | G/G |
| Control-0777 | 2 | 69 | 0 | 0 | 0 |  | G/G | G/G | G/G |
| Control-0778 | 1 | 52 | 0 | 0 | 0 |  | G/G | G/G | G/G |
| Control-0779 | 2 | 69 | 0 | 0 | 0 |  | G/G | G/G | G/G |
| Control-0780 | 2 | 83 | 0 | 0 | 1 |  | G/G | G/G | G/G |
| Control-0781 | 2 | 68 | 0 | 0 | 0 |  | G/A | G/G | G/A |
| Control-0782 | 2 | 61 | 0 | 0 | 0 |  | G/G | G/G | G/G |
| Control-0783 | 2 | 53 | 0 | 0 | 1 |  | G/G | G/G | G/G |
| Control-0784 | 2 | 74 | 0 | 0 | 1 |  | G/G | G/G | G/G |
| Control-0785 | 2 | 67 | 0 | 0 | 1 |  | G/G | G/G | G/G |
| Control-0786 | 2 | 73 | 0 | 0 | 0 |  | G/G | G/G | G/G |
| Control-0787 | 2 | 64 | 0 | 0 | 0 |  | G/A | G/A | G/G |
| Control-0788 | 2 | 61 | 0 | 0 | 1 |  | G/G | G/G | G/G |
| Control-0789 | 2 | 68 | 0 | 0 | 1 |  | G/G | G/G | G/G |
| Control-0790 | 2 | 62 | 0 | 0 | 1 |  | G/A | G/A | G/G |
| Control-0791 | 2 | 75 | 0 | 0 | 1 |  | G/A | A/A | G/A |
| Control-0792 | 2 | 65 | 0 | 0 | 1 |  | G/G | G/G | G/G |
| Control-0793 | 2 | 69 | 0 | 0 | 1 |  | G/A | G/A | G/G |
| Control-0794 | 2 | 48 | 0 | 0 | 0 |  | G/A | G/G | G/A |
| Control-0795 | 2 | 63 | 0 | 0 | 1 |  | G/G | G/G | G/G |
| Control-0796 | 2 | 41 | 0 | 0 | 0 |  | G/A | G/A | G/G |
| Control-0797 | 2 | 49 | 0 | 0 | 0 |  | G/G | G/G | G/G |
| Control-0798 | 2 | 66 | 0 | 0 | 0 |  | G/A | G/A | G/G |
| Control-0799 | 2 | 45 | 0 | 0 | 0 |  | G/A | G/G | G/G |
| Control-0800 | 2 | 45 | 0 | 0 | 1 |  | G/G | G/G | G/G |
| Control-0801 | 2 | 46 | 0 | 0 | 1 |  | G/G | G/G | G/G |
| Control-0802 | 2 | 67 | 0 | 0 | 0 |  | G/G | G/G | G/A |
| Control-0803 | 2 | 65 | 0 | 0 | 0 |  | G/A | G/A | G/A |
| Control-0804 | 2 | 74 | 0 | 0 | 0 |  | G/G | G/G | G/G |
| Control-0805 | 2 | 62 | 0 | 0 | 0 |  | G/G | G/A | G/A |
| Control-0806 | 2 | 55 | 0 | 0 | 0 |  | G/G | G/G | G/A |
| Control-0807 | 2 | 59 | 0 | 0 | 1 |  | G/G | G/G | G/G |
| Control-0808 | 2 | 59 | 1 | 1 | 1 |  | G/G | G/G | G/G |
| Control-0809 | 2 | 57 | 0 | 0 | 1 |  | G/G | G/A | G/A |
| Control-0810 | 2 | 64 | 0 | 0 | 0 |  | G/G | G/G | G/G |
| Control-0811 | 2 | 58 | 0 | 0 | 1 |  | G/G | G/G | G/G |
| Control-0812 | 2 | 64 | 0 | 0 | 1 |  | G/A | G/G | G/G |
| Control-0813 | 2 | 62 | 0 | 0 | 0 |  | G/G | G/G | G/G |
| Control-0814 | 2 | 63 | 0 | 0 | 0 |  | G/G | G/G | G/A |
| Control-0815 | 2 | 56 | 0 | 0 | 1 |  | G/G | G/G | G/G |
| Control-0816 | 1 | 57 | 0 | 0 | 0 |  | G/G | G/G | G/A |
| Control-0817 | 1 | 54 | 0 | 0 | 1 |  | G/A | G/G | G/G |
| Control-0818 | 1 | 52 | 0 | 0 | 0 |  | G/A | G/G | G/G |
| Control-0819 | 2 | 52 | 0 | 0 | 0 |  | G/G | G/G | G/G |
| Control-0820 | 1 | 53 | 0 | 0 | 0 |  | G/G | G/G | G/G |
| Control-0821 | 1 | 59 | 0 | 0 | 0 |  | G/A | G/G | G/G |
| Control-0822 | 1 | 61 | 0 | 0 | 0 |  | G/A | G/A | G/A |
| Control-0823 | 2 | **66** | 0 | **0** | 0 |  | G/G | G/G | G/G |
| Control-0824 | 1 | 63 | 0 | 0 | 0 |  | G/G | G/G | G/G |
| Control-0825 | 1 | 57 | 0 | 0 | 1 |  | G/G | G/G | G/G |
| Control-0826 | 1 | 67 | 0 | 0 | 0 |  | G/A | G/G | G/G |
| Control-0827 | 1 | 65 | 0 | 0 | 1 |  | G/G | G/G | G/G |
| Control-0828 | 1 | 56 | 0 | 0 | 1 |  | G/G | G/A | G/G |
| Control-0829 | 1 | 67 | 0 | 0 | 0 |  | G/A | G/A | G/G |
| Control-0830 | 1 | 71 | 1 | 1 | 0 |  | G/G | G/G | G/A |
| Control-0831 | 1 | 65 | 0 | 0 | 0 |  | G/G | G/G | G/G |
| Control-0832 | 1 | 55 | 0 | 0 | 0 |  | G/A | G/G | G/G |
| Control-0833 | 1 | 68 | 0 | 0 | 1 |  | G/G | G/G | G/G |
| Control-0834 | 2 | 53 | 0 | 0 | 0 |  | G/G | G/G | G/G |
| Control-0835 | 2 | 65 | 0 | 0 | 0 |  | G/G | G/G | G/G |
| Control-0836 | 2 | 72 | 0 | 0 | 0 |  | G/A | G/G | G/G |
| Control-0837 | 1 | 65 | 1 | 0 | 1 |  | G/G | G/G | G/G |
| Control-0838 | 1 | 65 | 1 | 0 | 0 |  | G/G | G/G | G/G |
| Control-0839 | 2 | 68 | 0 | 0 | 1 |  | G/G | G/G | G/G |
| Control-0840 | 1 | 62 | 0 | 0 | 1 |  | G/G | G/G | G/G |
| Control-0841 | 1 | 52 | 0 | 0 | 1 |  | G/G | G/G | G/G |
| Control-0842 | 1 | 70 | 1 | 1 | 0 |  | G/G | G/G | G/A |
| Control-0843 | 2 | 49 | 0 | 0 | 1 |  | G/G | G/G | G/G |
| Control-0844 | 2 | 56 | 0 | 0 | 0 |  | G/G | G/G | G/G |
| Control-0845 | 2 | 64 | 0 | 0 | 1 |  | G/G | G/G | G/G |
| Control-0846 | 2 | 68 | 0 | 0 | 0 |  | G/A | G/A | G/A |
| Control-0847 | 2 | 53 | 0 | 0 | 0 |  | G/G | G/G | G/G |
| Control-0848 | 2 | 54 | 0 | 0 | 0 |  | G/G | G/G | G/G |
| Control-0849 | 2 | 68 | 0 | 0 | 0 |  | A/A | A/A | A/A |
| Control-0850 | 2 | 49 | 0 | 0 | 1 |  | G/A | G/A | G/G |
| Control-0851 | 2 | 61 | 0 | 0 | 0 |  | G/G | G/A | G/A |
| Control-0852 | 2 | 59 | 0 | 0 | 0 |  | G/A | G/A | G/A |
| Control-0853 | 2 | 57 | 0 | 0 | 0 |  | G/G | G/G | G/G |
| Control-0854 | 2 | 52 | 0 | 0 | 1 |  | G/G | G/G | G/G |
| Control-0855 | 2 | 58 | 0 | 0 | 0 |  | G/A | G/A | G/A |
| Control-0856 | 2 | 64 | 0 | 0 | 0 |  | G/G | G/G | G/G |
| Control-0857 | 2 | 59 | 0 | 0 | 1 |  | G/G | G/G | G/G |
| Control-0858 | 2 | 58 | 0 | 0 | 0 |  | G/G | G/G | G/G |
| Control-0859 | 2 | 62 | 0 | 0 | 0 |  | G/G | G/G | G/G |
| Control-0860 | 2 | 63 | 0 | 0 | 1 |  | G/G | G/G | G/G |
| Control-0861 | 2 | 73 | 0 | 0 | 0 |  | G/G | G/G | G/G |
| Control-0862 | 2 | 59 | 0 | 0 | 0 |  | G/A | G/G | G/G |
| Control-0863 | 2 | 77 | 0 | 0 | 1 |  | G/G | G/G | G/G |
| Control-0864 | 2 | 59 | 0 | 0 | 0 |  | G/G | G/G | G/A |
| Control-0865 | 2 | 59 | 0 | 0 | 0 |  | G/G | G/G | G/G |
| Control-0866 | 2 | 73 | 0 | 0 | 1 |  | G/A | G/A | G/G |
| Control-0867 | 2 | 60 | 0 | 0 | 1 |  | G/G | G/G | G/G |
| Control-0868 | 2 | 73 | 0 | 0 | 0 |  | G/G | G/G | G/G |
| Control-0869 | 2 | 59 | 0 | 0 | 1 |  | G/A | G/A | G/A |
| Control-0870 | 2 | 71 | 0 | 0 | 1 |  | G/A | G/G | G/A |
| Control-0871 | 2 | 59 | 0 | 0 | 1 |  | G/G | G/G | G/G |
| Control-0872 | 2 | 62 | 0 | 0 | 0 |  | G/A | G/A | G/A |
| Control-0873 | 2 | 76 | 0 | 0 | 1 |  | G/A | G/G | G/G |
| Control-0874 | 2 | 56 | 0 | 0 | 0 |  | G/G | G/G | G/G |
| Control-0875 | 2 | 55 | 0 | 0 | 1 |  | G/A | G/A | G/A |
| Control-0876 | 2 | 62 | 0 | 0 | 0 |  | G/G | G/G | G/G |
| Control-0877 | 2 | 62 | 0 | 0 | 1 |  | A/A | A/A | A/A |
| Control-0878 | 1 | 57 | 0 | 0 | 0 |  | G/G | G/G | G/G |
| Control-0879 | 1 | 52 | 0 | 0 | 0 |  | ？ | ？ | ？ |
| Control-0880 | 1 | 54 | 0 | 0 | 1 |  | G/G | G/G | G/G |
| Control-0881 | 1 | 56 | 0 | 0 | 1 |  | G/G | G/G | G/A |
| Control-0882 | 1 | 52 | 0 | 0 | 0 |  | G/G | G/G | G/G |
| Control-0883 | 1 | 50 | 0 | 0 | 0 |  | G/G | G/G | G/G |
| Control-0884 | 1 | 62 | 0 | 0 | 0 |  | G/G | G/G | G/A |
| Control-0885 | 1 | 40 | 0 | 0 | 1 |  | G/G | G/G | G/G |
| Control-0886 | 1 | 49 | 0 | 0 | 1 |  | G/A | G/A | G/A |
| Control-0887 | 1 | 53 | 0 | 0 | 1 |  | G/G | G/G | G/A |
| Control-0888 | 1 | 45 | 0 | 0 | 0 |  | G/A | G/A | G/A |
| Control-0889 | 1 | 48 | 0 | 0 | 0 |  | G/A | G/G | G/G |
| Control-0890 | 1 | 43 | 0 | 0 | 0 |  | G/G | G/G | G/G |
| Control-0891 | 1 | 61 | 0 | 0 | 1 |  | G/G | G/G | G/G |
| Control-0892 | 1 | 51 | 0 | 0 | 1 |  | G/A | G/G | G/G |
| Control-0893 | 1 | 69 | 0 | 0 | 1 |  | G/G | G/G | G/G |
| Control-0894 | 1 | 55 | 0 | 0 | 1 |  | G/A | G/A | G/A |
| Control-0895 | 2 | 50 | 0 | 0 | 1 |  | G/G | G/G | G/A |
| Control-0896 | 2 | 56 | 0 | 0 | 1 |  | G/G | G/G | G/G |
| Control-0897 | 1 | 53 | 1 | 1 | 0 |  | G/A | G/G | G/G |
| Control-0898 | 2 | 55 | 0 | 0 | 0 |  | G/G | G/G | G/G |
| Control-0899 | 1 | 62 | 0 | 0 | 1 |  | G/G | G/G | G/G |
| Control-0900 | 1 | 49 | 0 | 0 | 1 |  | G/A | G/G | G/G |
| Control-0901 | 1 | 63 | 0 | 0 | 0 |  | G/G | G/G | G/G |
| Control-0902 | 2 | 61 | 0 | 0 | 1 |  | A/A | A/A | G/A |
| Control-0903 | 1 | 51 | 0 | 0 | 1 |  | A/A | A/A | G/A |
| Control-0904 | 1 | 47 | 0 | 0 | 0 |  | G/G | G/G | G/G |
| Control-0905 | 1 | 49 | 0 | 0 | 0 |  | G/G | G/G | G/G |
| Control-0906 | 2 | 62 | 0 | 0 | 1 |  | G/G | G/G | G/G |
| Control-0907 | 2 | 68 | 0 | 0 | 1 |  | G/A | G/A | G/A |
| Control-0908 | 1 | 40 | 0 | 0 | 0 |  | G/A | G/A | G/A |
| Control-0909 | 1 | 59 | 0 | 0 | 0 |  | G/A | G/A | G/A |
| Control-0910 | 1 | 49 | 0 | 0 | 0 |  | G/G | G/G | G/G |
| Control-0911 | 1 | 63 | 0 | 0 | 0 |  | G/G | G/G | G/G |
| Control-0912 | 1 | 49 | 0 | 0 | 1 |  | G/G | G/G | G/G |
| Control-0913 | 2 | 67 | 0 | 0 | 1 |  | G/G | G/G | G/G |
| Control-0914 | 1 | 66 | 0 | 0 | 0 |  | G/G | G/G | G/G |
| Control-0915 | 1 | 42 | 1 | 1 | 0 |  | G/G | G/G | G/A |
| Control-0916 | 1 | 41 | 0 | 0 | 0 |  | G/G | G/G | G/A |
| Control-0917 | 2 | 56 | 0 | 0 | 1 |  | G/A | G/A | G/A |
| Control-0918 | 1 | 60 | 1 | 1 | 0 |  | G/G | G/G | G/G |
| Control-0919 | 1 | 65 | 1 | 0 | 0 |  | G/G | G/G | G/G |
| Control-0920 | 1 | 55 | 0 | 0 | 0 |  | G/G | G/G | G/G |
| Control-0921 | 1 | 63 | 1 | 1 | 0 |  | G/G | G/G | G/G |
| Control-0922 | 2 | 40 | 0 | 0 | 0 |  | G/A | G/A | G/A |
| Control-0923 | 1 | 50 | 0 | 1 | 0 |  | G/G | G/G | G/G |
| Control-0924 | 1 | 50 | 1 | 0 | 0 |  | G/G | G/G | G/G |
| Control-0925 | 2 | 53 | 0 | 0 | 1 |  | G/A | G/G | G/G |
| Control-0926 | 2 | 55 | 0 | 0 | 0 |  | G/A | G/A | G/A |
| Control-0927 | 2 | 57 | 0 | 0 | 1 |  | G/G | G/G | G/G |
| Control-0928 | 2 | 56 | 0 | 0 | 0 |  | G/G | G/G | G/G |
| Control-0929 | 2 | 60 | 0 | 0 | 1 |  | G/G | G/G | G/A |
| Control-0930 | 2 | 51 | 0 | 0 | 1 |  | G/G | G/G | G/G |
| Control-0931 | 2 | 61 | 0 | 0 | 1 |  | G/A | G/A | G/A |
| Control-0932 | 2 | 52 | 0 | 0 | 0 |  | G/G | G/G | G/G |
| Control-0933 | 2 | 56 | 0 | 0 | 0 |  | G/G | G/G | G/G |
| Control-0934 | 2 | 60 | 0 | 0 | 0 |  | G/G | G/G | G/G |
| Control-0935 | 2 | 58 | 0 | 0 | 0 |  | A/A | A/A | A/A |
| Control-0936 | 2 | 59 | 0 | 0 | 0 |  | G/G | G/G | G/G |
| Control-0937 | 2 | 60 | 0 | 0 | 1 |  | A/A | G/G | G/G |
| Control-0938 | 2 | 53 | 0 | 0 | 1 |  | G/G | G/G | G/G |
| Control-0939 | 2 | 56 | 0 | 0 | 0 |  | G/G | G/G | G/G |
| Control-0940 | 2 | 61 | 0 | 0 | 1 |  | G/A | G/G | G/G |
| Control-0941 | 2 | 59 | 0 | 0 | 0 |  | G/G | G/G | G/G |
| Control-0942 | 2 | 57 | 0 | 0 | 0 |  | G/G | G/A | G/G |
| Control-0943 | 2 | 60 | 0 | 0 | 0 |  | G/G | G/G | G/A |
| Control-0944 | 2 | 32 | 0 | 0 | 0 |  | G/G | G/G | G/G |
| Control-0945 | 2 | 50 | 0 | 0 | 1 |  | G/G | G/G | G/A |
| Control-0946 | 2 | 56 | 0 | 0 | 0 |  | G/A | G/G | G/G |
| Control-0947 | 2 | 60 | 0 | 0 | 1 |  | G/G | G/G | G/G |
| Control-0948 | 2 | 58 | 0 | 0 | 1 |  | G/G | G/G | G/G |
| Control-0949 | 2 | 50 | 0 | 0 | 1 |  | G/A | G/A | G/A |
| Control-0950 | 2 | 52 | 0 | 0 | 1 |  | G/G | G/G | G/G |
| Control-0951 | 1 | 69 | 0 | 0 | 0 |  | G/G | G/G | G/G |
| Control-0952 | 2 | 79 | 0 | 0 | 1 |  | G/G | G/G | G/G |
| Control-0953 | 1 | 63 | 0 | 0 | 1 |  | G/G | G/A | G/G |
| Control-0954 | 1 | 61 | 0 | 0 | 0 |  | G/G | G/G | G/G |
| Control-0955 | 1 | 55 | 0 | 0 | 1 |  | G/A | G/G | G/A |
| Control-0956 | 1 | 77 | 0 | 0 | 0 |  | G/A | G/A | G/A |
| Control-0957 | 1 | 63 | 0 | 0 | 0 |  | G/A | G/A | G/A |
| Control-0958 | 2 | 61 | 0 | 0 | 1 |  | G/A | G/G | G/G |
| Control-0959 | 2 | 69 | 0 | 0 | 0 |  | G/A | G/A | G/A |
| Control-0960 | 2 | 47 | 0 | 0 | 0 |  | G/G | G/G | G/G |
| Control-0961 | 1 | 46 | 0 | 0 | 0 |  | G/G | G/G | G/G |
| Control-0962 | 1 | 65 | 1 | 1 | 0 |  | G/G | G/G | G/A |
| Control-0963 | 1 | 50 | 0 | 0 | 1 |  | G/A | G/A | G/A |
| Control-0964 | 2 | 76 | 0 | 0 | 1 |  | G/G | G/G | G/G |
| Control-0965 | 2 | 53 | 0 | 0 | 1 |  | G/A | G/G | G/G |
| Control-0966 | 1 | 65 | 0 | 0 | 1 |  | G/G | G/G | G/G |
| Control-0967 | 1 | 38 | 0 | 0 | 1 |  | G/A | G/A | G/A |
| Control-0968 | 2 | 46 | 0 | 0 | 0 |  | G/G | G/G | G/G |
| Control-0969 | 1 | 32 | 1 | 1 | 0 |  | G/G | G/G | G/A |
| Control-0970 | 2 | 69 | 0 | 0 | 0 |  | G/G | G/G | G/G |
| Control-0971 | 1 | 74 | 0 | 0 | 0 |  | G/A | G/A | G/G |
| Control-0972 | 1 | 58 | 1 | 0 | 1 |  | G/G | G/G | G/G |
| Control-0973 | 2 | 45 | 0 | 0 | 0 |  | G/G | G/G | G/G |
| Control-0974 | 1 | 61 | 1 | 0 | 1 |  | G/G | G/G | G/G |
| Control-0975 | 1 | 34 | 0 | 0 | 0 |  | G/A | G/G | G/G |
| Control-0976 | 1 | 68 | 0 | 0 | 0 |  | G/G | G/G | G/G |
| Control-0977 | 2 | 47 | 0 | 0 | 1 |  | G/G | G/G | G/G |
| Control-0978 | 2 | 30 | 0 | 0 | 0 |  | G/G | G/G | G/G |
| Control-0979 | 1 | 57 | 0 | 1 | 0 |  | G/A | G/A | G/A |
| Control-0980 | 2 | 46 | 0 | 0 | 1 |  | G/G | G/G | G/G |
| Control-0981 | 2 | 58 | 0 | 0 | 0 |  | G/A | G/G | G/G |
| Control-0982 | 1 | 44 | 1 | 1 | 1 |  | G/A | G/A | G/A |
| Control-0983 | 1 | 58 | 0 | 1 | 1 |  | ? | ? | ? |
| Control-0984 | 1 | 61 | 1 | 0 | 0 |  | G/A | G/G | G/A |
| Control-0985 | 1 | 57 | 0 | 0 | 0 |  | G/G | G/G | G/G |
| Control-0986 | 2 | 63 | 0 | 0 | 1 |  | G/G | G/G | G/G |
| Control-0987 | 2 | 49 | 1 | 1 | 0 |  | G/G | G/G | G/G |
| Control-0988 | 2 | 64 | 0 | 0 | 0 |  | G/G | G/G | G/G |
| Control-0989 | 2 | 36 | 0 | 0 | 0 |  | G/A | G/A | G/G |
| Control-0990 | 1 | 59 | 0 | 0 | 0 |  | G/G | G/G | G/G |
| Control-0991 | 2 | 44 | 0 | 0 | 0 |  | G/G | G/G | G/G |
| Control-0992 | 2 | 62 | 0 | 0 | 0 |  | G/G | G/G | G/G |
| Control-0993 | 2 | 73 | 0 | 0 | 1 |  | G/A | G/G | G/A |
| Control-0994 | 1 | 60 | 1 | 0 | 0 |  | G/A | G/A | G/G |
| Control-0995 | 2 | 62 | 0 | 0 | 0 |  | G/G | G/G | G/G |
| Control-0996 | 1 | 51 | 1 | 0 | 1 |  | G/A | G/A | G/A |
| Control-0997 | 1 | 73 | 0 | 0 | 1 |  | G/G | G/G | G/G |
| Control-0998 | 1 | 75 | 0 | 0 | 0 |  | G/G | G/G | G/G |
| Control-0999 | 2 | 47 | 0 | 0 | 1 |  | G/G | G/G | G/G |
| Control-1000 | 1 | 44 | 0 | 0 | 0 |  | G/G | G/G | G/G |
| Control-1001 | 1 | 79 | 0 | 0 | 0 |  | G/G | G/G | G/G |
| Control-1002 | 2 | 65 | 0 | 0 | 1 |  | G/G | G/G | G/G |
| Control-1003 | 1 | 68 | 0 | 0 | 0 |  | G/G | G/G | G/G |
| Control-1004 | 2 | 43 | 0 | 0 | 0 |  | G/G | G/G | G/G |
| Control-1005 | 1 | 79 | 0 | 0 | 1 |  | G/G | G/G | G/G |
| Control-1006 | 2 | 51 | 0 | 0 | 0 |  | G/G | G/G | G/G |
| Control-1007 | 1 | 39 | 0 | 0 | 1 |  | G/A | G/A | G/G |
| Control-1008 | 2 | 68 | 0 | 0 | 0 |  | G/A | G/A | G/A |
| Control-1009 | 2 | 75 | 0 | 0 | 0 |  | ? | ? | ? |
| Control-1010 | 2 | 63 | 0 | 0 | 0 |  | G/G | G/G | G/G |
| Control-1011 | 2 | 44 | 0 | 0 | 1 |  | G/A | G/A | G/A |
| Control-1012 | 1 | 60 | 0 | 0 | 0 |  | G/A | G/A | G/G |
| Control-1013 | 1 | 86 | 0 | 0 | 0 |  | G/A | G/A | G/A |
| Control-1014 | 1 | 47 | 0 | 0 | 0 |  | G/G | G/G | G/G |
| Control-1015 | 2 | 41 | 0 | 1 | 1 |  | G/A | G/A | G/A |
| Control-1016 | 2 | 79 | 0 | 0 | 0 |  | G/G | G/G | G/G |
| Control-1017 | 1 | 67 | 1 | 1 | 0 |  | G/G | G/G | G/A |
| Control-1018 | 1 | 46 | 0 | 0 | 0 |  | G/G | G/G | G/G |
| Control-1019 | 2 | 56 | 0 | 0 | 1 |  | G/G | G/A | G/G |
| Control-1020 | 2 | 74 | 0 | 0 | 0 |  | G/G | G/G | G/G |
| Control-1021 | 1 | 76 | 0 | 0 | 0 |  | G/G | G/G | G/G |
| Control-1022 | 1 | 44 | 1 | 0 | 0 |  | G/A | G/G | G/G |
| Control-1023 | 1 | 43 | 0 | 0 | 1 |  | G/G | G/G | G/G |
| Control-1024 | 1 | 74 | 1 | 1 | 1 |  | G/G | G/G | G/G |
| Control-1025 | 2 | 68 | 0 | 0 | 0 |  | G/G | G/G | G/G |
| Control-1026 | 1 | 82 | 0 | 0 | 0 |  | G/A | G/A | G/G |
| Control-1027 | 2 | 78 | 0 | 0 | 1 |  | G/G | G/G | G/G |
| Control-1028 | 1 | 64 | 1 | 0 | 0 |  | G/G | G/A | G/G |
| Control-1029 | 1 | 36 | 1 | 1 | 0 |  | G/G | G/G | G/G |
| Control-1030 | 1 | 35 | 0 | 0 | 1 |  | G/G | G/G | G/G |
| Control-1031 | 1 | 63 | 0 | 0 | 0 |  | G/G | G/G | G/G |
| Control-1032 | 2 | 74 | 0 | 0 | 0 |  | G/G | G/G | G/G |
| Control-1033 | 2 | 63 | 0 | 0 | 0 |  | G/G | G/G | G/G |
| Control-1034 | 2 | 70 | 0 | 0 | 1 |  | G/G | G/G | G/G |
| Control-1035 | 1 | 63 | 0 | 0 | 0 |  | G/G | G/G | G/G |
| Control-1036 | 1 | 65 | 0 | 0 | 0 |  | G/G | G/G | G/G |
| Control-1037 | 1 | 80 | 0 | 0 | 1 |  | G/G | G/G | G/A |
| Control-1038 | 1 | 74 | 1 | 1 | 0 |  | G/G | G/G | G/G |
| Control-1039 | 1 | 55 | 0 | 0 | 1 |  | G/G | G/G | G/G |
| Control-1040 | 1 | 24 | 0 | 0 | 0 |  | G/G | G/G | G/G |
| Control-1041 | 2 | 71 | 0 | 0 | 0 |  | G/G | G/G | G/G |
| Control-1042 | 1 | 58 | 0 | 0 | 1 |  | G/G | G/G | G/G |
| Control-1043 | 1 | 53 | 1 | 1 | 0 |  | G/G | G/G | G/G |
| Control-1044 | 2 | 60 | 0 | 0 | 0 |  | G/G | G/G | G/G |
| Control-1045 | 1 | 78 | 0 | 0 | 1 |  | G/A | G/G | G/A |
| Control-1046 | 1 | 69 | 1 | 1 | 0 |  | G/G | G/G | G/G |
| Control-1047 | 1 | 53 | 0 | 0 | 1 |  | G/G | G/G | G/G |
| Control-1048 | 1 | 49 | 0 | 0 | 0 |  | G/G | G/G | G/G |
| Control-1049 | 2 | 78 | 0 | 0 | 1 |  | G/A | G/A | G/A |
| Control-1050 | 1 | 38 | 0 | 0 | 0 |  | G/A | G/A | G/G |
| Control-1051 | 1 | 47 | 1 | 1 | 0 |  | G/G | G/G | G/A |
| Control-1052 | 1 | 77 | 0 | 0 | 0 |  | G/A | G/G | G/G |
| Control-1053 | 2 | 62 | 0 | 0 | 0 |  | G/G | G/G | G/G |
| Control-1054 | 2 | 67 | 0 | 0 | 0 |  | G/A | G/A | G/A |
| Control-1055 | 1 | 54 | 1 | 0 | 1 |  | G/G | G/G | G/G |
| Control-1056 | 1 | 64 | 0 | 0 | 1 |  | G/G | G/G | G/G |

NSCLC: non-small-cell lung cancer;

SCC: [squamous cell carcinoma](javascript:;);
